# Supplementary material for: Biosynthesis and engineering of the nonribosomal peptides with a C-terminal putrescine
Source: Nat Commun. 2023 Oct 19;14:6619. doi: 10.1038/s41467-023-42387-z (PMC10587159; doi:10.1038/s41467-023-42387-z)
Supplement: Supplementary file 1 — Supplementary Information [file 41467_2023_42387_MOESM1_ESM.pdf]

Supplementary Information

Biosynthesis and Engineering of the Nonribosomal Peptides with a C-terminal Putrescine

Hanna Chen,[†,1,3] Lin Zhong,[†,2] Haibo Zhou,[†,1] Xianping Bai,[1] Tao Sun,[1] Xingyan Wang,[1] Yiming Zhao,[1] Xiaoqi Ji,[1] Qiang Tu,[2] Youming Zhang[1,2] & Xiaoying Bian[1,*]

[1]Helmholtz International Lab for Anti-infectives, Shandong University–Helmholtz Institute of Biotechnology, State Key Laboratory of Microbial Technology, Shandong University, Qingdao, Shandong, 266237, China.

[2]CAS Key Laboratory of Quantitative Engineering Biology, Shenzhen Institute of Synthetic Biology, Shenzhen Institute of Advanced Technology, Chinese Academy of Sciences, Shenzhen 518055, China.

[3]Present address: School of Medicine, Linyi University, Shuangling Road, Linyi, 276000, China.

† Contributed equally to this work.

***Corresponding authors**

Xiaoying Bian (bianxiaoying@sdu.edu.cn), ORCID: 0000-0002-1356-3211

Supplementary Note1: Detailed structure elucidation of 1

Glidonin A (**1**) was obtained as colourless oil with the molecular formula as $C_{65}H_{98}N_{16}O_{14}S$ (HR-ESI-MS, *m/z* 680.3699 [*M* + 2H]$^{2+}$, calcd 1359.7242). Interpretation of its ^{1}H, ^{13}C, and DEPT NMR spectra (Supplementary Table 2, in MeOD-d_4) revealed that **1** possesses 65 carbons, including thirteen carbonyls [δ_C 175.8, 175.0, 175.0, 174.2, 174.2, 174.1 (2C), 173.8, 173.2, 172.6 (2C), 171.5, 169.8], fourteen olefinic carbons [δ_C 138.0, 137.5, 130.6 (2C), 129.6 (2C), 128.6, 128.2, 124.8, 122.6, 119.9, 119.2, 112.5, 110.8] consistent with a monosubstituted phenyl group of a phenylalanine (Phe) and a indole moiety of a tryptophan (Trp), sixteen methylenes (δ_C 62.7, 48.7, 48.4, 40.4, 39.2, 38.9, 37.3, 32.0, 30.7, 30.0, 29.9, 28.0, 27.1, 25.6, 25.6, 24.9) including one *O*-CH_2 group belongs to serine (Ser) and four *N*-CH_2 groups, fourteen methines including 12 α-methines of amino acids derivatives ($\delta_{H/C}$ 4.14/62.3, 4.54/62.0, 4.03/61.0, 4.32/59.6, 4.31/57.7, 4.65/55.8, 4.62/54.9, 4.03/53.7, 4.41/51.1, 4.31/51.0, 4.31/50.9, 5.01/50.1), seven methyl doublets (δ_C 20.0, 19.5, 19.1, 19.0, 17.8, 17.4, 17.3), and one methyl singlet (δ_H/δ_C 2.11/15.2) corresponding to methionine (Met). Further 2D NMR data analyses enabled us to assign the four *N*-CH_2 groups and seven doublet methyls to be part of two prolines (Pro), one putrescine (Put), and two valines (Val) and three alanines (Ala), respectively. The remaining one carbonyl and one methylene were deduced to be asparagine (Asn) according to the observed COSY and HMBC correlations (Supplementary Fig. 3). Thus, compound **1** was confirmed to be a dodecapeptide containing the following amino acid moieties: Met, Asn, two Pro, two Val, Phe, Trp, three Ala, Ser, and one Put residue. The sequence of the amino acids and the location of the putrescine were established by detailed interpretation of the HMBC correlations from amide protons and adjacent carbonyl groups (Supplementary Fig. 3, in DMSO-d_6). The planar structure of **1** was subsequently corroborated by HR-ESI-MS/MS fragmentation analysis (Supplementary Fig. 9a, b).

Supplementary Note2: The substrate promiscuity of acylase GdnD

We investigate the substrate specificity of GdnD. At first, we predicted the structure of GdnD using AlphaFold2[1], which is "V"-shaped structure, and its substrate pocket is open that can accommodate broad substrates (Supplementary Figs. 5b, 21a). The biochemical assays showed the nonacylated products **13a**, **14a** and **15a** were detected from reaction mixing with three glidonin-like shorter lipopeptides **13**, **14** and **15**,

respectively, indicating GdnD can hydrolyse lipopeptides with L-/D- configuration of first amino acids and different lengths of peptide chains from dipeptide to dodecapeptide (Fig. 2c, e, Supplementary Table 14, Supplementary Fig. 22a). Moreover, GdnD was functional for other exogenous lipopeptides except short acyl chains because the GdnD could remove myristoyl of cyclic lipopeptide C14-rhizomide A (**17**) to yield nonacylated product **17a** but not acetyl of rhizomide A (**16**) (Fig. 2f, Supplementary Fig. 22a).

The PvdQ shows a heart-shaped structure (“V”-shaped structure) formed from two interleaved chains, and a hydrophobic pocket that presents the catalytic Serβ1 responsible for the acyl hydrolysis.[2,3] The residues of lining of substrate-binding site are highly conserved among PvdQ homologues.[4] Numerous studies have reported that PvdQ acylases can hydrolyze various substrates, including different N-acyl-homoserine lactones (HSLs)[5], telomycins (C8-C12)[6], and some siderophores crochelins (C10-C12)[7] and marinobactins (C12-C16)[8], whereas the concrete mechanism of PvdQ inclined to broad substrates is not the main focus. But, the substrate promiscuity of acylase GdnD was investigated based on the reported PvdQ structures.

The crystal structures of PvdQ (PDB number 5UBK and 3L91) show that the protein adopts a “V”-shaped structure, with a hydrophobic pocket located at the vertex of the “V” that can accommodate N-terminal different fatty acid chains of substrates (Supplementary Fig. 5b, c)[2,4]. According to these characteristics, the substrates are disfavoured when the N-terminal length is either too short to reach the hydrophobic pocket or too long to fit inside it (Supplementary Fig. 5c)[5]. Residues W400 (W402 in PvdQ), V284 (V286 in PvdQ), F238 (F240 in PvdQ), F246 (F248 in PvdQ), L264 (L266 in PvdQ), W377 (W378 in PvdQ), L267 (L269 in PvdQ), M373 (V374 in PvdQ), V401 (V403 in PvdQ), form a hydrophobic pocket in both GdnD and PvdQ structures (Supplementary Fig. 21c). Thus, we speculated that GdnD might follow a similar principle when it hydrolyzed lipopeptides with varying lengths of peptide chains.

In addition, GdnD can hydrolyse lipopeptides with both L-/D- configuration of the first amino acid and various amino acids in the backbone. This observation could be attributed to the accessibility of the entrance of the “V”-shaped pocket due to its open conformation at C-terminus (Supplementary Fig. 21a, b). The binding pocket for the substrate’s C-terminus, which is located at the entrance of the “V”-shaped pocket, is relatively open and can accommodate larger and different C-terminal groups to enter.

In summary, the binding pocket for the C-terminus shows a wider substrate promiscuity compared to the binding pocket for the N-terminus.

Supplementary Note3: Formation of formyl products mediated by a methionyl-tRNA formyltransferase

N-formylation is a rare event in NRPs, even though it is a common mechanism for initiation of ribosomal protein biosynthesis in prokaryotes.[9] The N-terminal formylation of three NRPs gramicidin[10], anabaenopeptilides[11] and szentiamide[12,13] was catalyzed by N-terminal formylation (F) domain, respectively. However, the lack of F domain in the *gdn* BGC hints that the formation of the *N*-formylation of glidonin is clearly different with the above three NRPs. Genome analysis showed the genome of *Escherichia coli* BL21 and *Schlegelella brevitalea* DSM 7029 both included three common formyltransferases family containing phosphoribosylglycinamide formyltransferase (PurN), phosphoribosylaminoimidazolecarboxamide formyltransferase (PurH), and methionyl-tRNA formyltransferase, suggesting the formyl moiety of **4** might be catalyzed by the formyltransferase(s) (Supplementary Table 13). Then, we successfully constructed two knock-out mutants of *purN* and *purH* with the fragile growths, but failed to obtain the inactivation of methionyl-tRNA formyltransferase. The metabolite analysis showed the two mutants still could produce the glidonins (Supplementary Fig. 22e), suggesting the formylation might be catalyzed by methionyl-tRNA formyltransferase (defined as Fmt protein). Subsequently, we purified this protein, and carried out *in vitro* assay of protein Fmt with different substrates **1**, **1a**, and **13a**. LC-MS profiles showed that the yield of formyl product (**4**) of **1** was higher than control (**1** was mixture containing a trace mass of **4**), and the other formyl products **1b** and **13b** also could be detected in reaction mixture, respectively (Supplementary Fig. 22b, c, d). Thus, the formation of formyl moiety was catalyzed by methionyl-tRNA formyltransferase.

Supplementary Note4: Screening the bioactivity of glidonins and derivatives

The bioactivity of glidonins was screened by several assays. The cytotoxicity assay showed lauroyl products **7** and **9** and myristoyl product **10** exhibited not only broad-spectrum cytotoxicity, but also strong activities against several tumour lines (Supplementary Table 11). Particularly, **10** exhibited potent cytotoxicity against human acute lymphoblastic leukemia cell line Kasumi and human hepatoma cell line HepG2

with IC_{50} value of 1.39 μM and 6.68 μM, while **9** showed strong bioactivities against human lung adenocarcinoma cell line A549 and human breast adenocarcinoma cell line MDA-MB-231 with IC_{50} value of 3.26 μM and 4.26 μM, respectively. Moreover, nonacylated compound **1** only showed moderate activities against two cell lines Kasumi and A549. In addition, they had no antimicrobial activities against four tested bacteria including *E. coli*, *Pseudomonas aeruginosa*, *Staphylococcus aureus* and *Bacillus subtilis*, and three tested fungi including *Candida albicans*, *Rhizoctonia solani* and *Aspergillus fumigatus*.

To determine whether the difference in bioactivity of products (with or without Put moiety) is due to variations in cellular uptake, we treated hepatoma cell line HepG2 with compounds **7** (C12 acyl), **10** (C14 acyl), and **10a** (C14 acyl and without Put) (Supplementary Fig. 33c). The cells were treated for 3 h, and then culture medium and cells were extracted separately. LC-MS was used to detect and quantify the compounds. At lower concentrations (5000 ng/mL, and 10,000 ng in total for each group), and shorter periods of treatment, none of the three products showed inhibition of cells (Supplementary Fig. 33a). The results suggest that the compounds with strong activity (**7** and **10**) have lower total quantities (intracellular plus extracellular) compared to **10a**, indicating that differences in cell metabolism between the two compounds (**10** vs **10a**) may be contributing to the differences in antitumor activity (Supplementary Fig. 33b). This could be due to differences in the speed of cellular uptake or differences in drug metabolism rate, requiring further experimental investigation.

Supplementary Table 1. Genes of glidonin biosynthetic gene cluster in *S. brevitalea* DSM 7029 and their closest protein homologs based on BlastP analysis

Locus Tag	Accession	Annotation	Homolog Accession	Organism	Identities (% ID)
gdnA	AKJ30063.1	NRPS	-	-	-
gdnB	AKJ30062.1	NRPS	-	-	-
gdnC	AKJ30060.1	ABC transporter ATP-binding protein/permease	KAF1006891.1	*Luteibacter* sp.	68%
+1	AKJ30059.1	Hypothetical protein	TET96141.1	*Dehalococcoidia* bacterium	35%
-1	AKJ30064.1	Hypothetical protein	-	-	-
-2	AKJ30065.1	ABC transporter substrate-binding protein	WP_047584935.1	*Methylibium* SP.YR605	61%
-3	AKJ30066.1	Methyl-accepting chemotaxis protein	WP_16046440.1	*Nocardioides* sp. SLBN-35	61%

Supplementary Table 2. The ^{1}H (500 MHz) and ^{13}C NMR (125 MHz) Data of 1 in MeOD-d_4 and DMSO-d_6

	no	**1**[b]		**1**[c]	
		δ_C, Type	δ_H (J in Hz)	δ_C, Type	δ_H (J in Hz)
Met	1	169.8, C		167.7, C	
	2	53.7, CH	4.03, m[a]	51.5, CH	3.82, m[a]
	3a	32.0, CH_2	2.24, m[a]	31.0, CH_2	1.96, m[a]
	3b		2.16, m[a]		1.91, m[a]
	4	30.0, CH_2	2.63, t (7.8)	28.2, CH_2	2.46, dd (8.0, 16.8)
	5	15.2, CH_3	2.11, s	14.4, CH_3	2.03, s
	2-NH				8.19, brs
Asn	1	174.2, C		172.0, C	
	2	50.1, CH	5.01, t (7.5)	47.2, CH	4.98, m
	3a	37.3, CH_2	2.81, dd (7.5, 15.5)	37.1, CH_2	2.67, dd (7.8, 15.5)
	3b		2.63, m[a]		2.29, dd (6.0, 15.5)
	4	171.5, C		168.9, C	
	2-NH				8.81, d (8.2)
Pro1	1	174.2, C		171.1, C	
	2	62.0, CH	4.54, dd (4.4, 7.0)	59.3, CH	4.35, m[a]
	3a	30.7, CH_2	2.14, m[a]	29.1, CH_2	1.82, m[a]
	3b				1.70, m[a]
	4	25.6, CH_2	1.98, m	24.2, CH_2	1.48, m[a]
	5a	48.7, CH_2	3.85, m[a]	46.9, CH_2	3.64, m[a]
	5b		3.78, m[a]		
Val1	1	173.2, C		170.8, C	
	2	59.6, CH	4.32, d (7.2)	57.0, CH	4.35, m[a]
	3	31.9, CH	2.14, m[a]	30.9, CH	1.70, m[a]
	4	20.0, CH_3	0.95, d (6.7)	19.2, CH_3	0.76, d (6.8)
	5	19.5, CH_3	0.94, d (6.7)	19.0, CH_3	0.75, d (6.8)
	2-NH				7.78, d (9.1)
Phe	1	172.6, C		169.7, C	
	2	54.9, CH	4.62, dd (6.7, 8.9)	52.5, CH	4.73, m
	3a	38.9, CH_2	3.05, dd (6.7, 13.0)	37.5, CH_2	2.90, d (6.8)
	3b		2.95, dd (8.9, 13.0)		
	4	137.5, C		136.9, C	
	5/9	130.6, CH	7.22, m[a]	129.3, CH	7.20, m[a]
	6/8	129.6, CH	7.27, m[a]	128.2, CH	7.25, t (6.8)
	7	128.2, CH	7.22, m[a]	126.6, CH	7.20, m[a]
	2-NH				8.34, d (7.0)
Pro2	1	173.8, C		171.2, C	
	2	62.3, CH	4.14, dd (4.0, 8.0)	59.8, CH	4.19, m[a]
	3a	30.0, CH_2	1.63, m[a]	28.8, CH_2	1.91, m[a]
	3b		1.57, m[a]		1.70, m[a]
	4a	24.9, CH_2	1.39, m[a]	23.8, CH_2	1.91, m[a]
	4b				1.82, m[a]
	5a	48.4, CH_2	3.50, m	46.7, CH_2	3.47, m
	5b		3.32, m[a]		3.39, m
Trp	1	174.1, C		171.6, C	
	2	55.8, CH	4.65, m[a]	53.8, CH	4.54, m
	3a	28.0, CH_2	3.32, m[a]	27.2, CH_2	3.14, dd (5.0, 13.1)
	3b		3.22, m[a]		2.98, dd (6.1, 13.1)
	4	110.8, C		110.0, C	
	5	128.6, C		127.2, C	
	6	119.2, CH	7.59, d (7.9)	118.2, CH	7.56, d (7.9)
	7	119.9, CH	6.99, t (7.9)	118.3, CH	6.95, t (7.9)
	8	122.6, CH	7.07, t (7.9)	120.8, CH	7.05, t (7.9)
	9	112.5, CH	7.31, t (7.9)	111.3, CH	7.30, d (7.9)
	10	138.0, C		136.1, C	
	11	124.8, CH	7.11, s	123.7, CH	7.15, s
	2-NH				7.95, d (6.8)
	11-NH				10.77, s
Val2	1	174.1, C		171.1, C	
	2	61.0, CH	4.03, m[a]	58.1, CH	4.06, dd (8.1, 15.4)
	3	31.2, CH	2.12, m[a]	29.8, CH	1.96, m[a]

	4	19.1, CH_3	0.83, d (7.5)	18.8, CH_3	0.69, d (6.7)
	5	19.0, CH_3	0.85, d (7.5)	18.8, CH_3	0.69, d (6.7)
	2-NH				7.81, d (8.1)
Ala1	1	175.0, C		172.3, C	
	2	50.9, CH	4.31, m[a]	48.4, CH	4.35, m[a]
	3	17.4, CH_3	1.40, d (7.4)	18.1, CH_3	1.22, d (6.4)
	2-NH				8.23, d (6.7)
Ala2	1	175.8, C		172.7, C	
	2	51.0, CH	4.31, m[a]	48.5, CH	4.27, m
	3	17.3, CH_3	1.42, d (7.1)	17.9, CH_3	1.24, d (7.7)
	2-NH				8.05, d (7.4)
Ser	1	172.6, C		169.7, C	
	2	57.7, CH	4.31, m[a]	55.0, CH	4.25, m
	3a	62.7, CH_2	3.91, dd (5.0, 12.0)	61.6, CH_2	3.63, dd (5.3, 11.0)
	3b		3.79, dd (5.0, 12.0)		3.54, dd (5.9, 11.0)
	2-NH				8.10, d (6.4)
Ala3	1	175.0, C		172.0, C	
	2	51.1, CH	4.41, q (7.2)	48.5, CH	4.19, m[a]
	3	17.8, CH_3	1.38, d (7.2)	17.7, CH_3	1.19, d (7.6)
	2-NH				8.06, d (8.2)
putrescine	1a	39.3, CH_2	3.27, m[a]	37.9, CH_2	3.08, m
	1b		3.16, m[a]		2.97, m[a]
	2	27.1, CH_2	1.57, m[a]	25.9, CH_2	1.41, m
	3	25.6, CH_2	1.63, m[a]	24.3, CH_2	1.48, m[a]
	4	40.4, CH_2	2.91, t (7.5)	38.5, CH_2	2.76, dd (6.2, 12.7)
	1-NH				7.68, t (5.3)
	4-NH				7.73, brs

[a] overlapped

[b] signals were recorded in MeOD-d_4 solvent on 500M NMR spectrometer

[c] signals were recorded in DMSO-d_6 solvent on 600M NMR spectrometer

Supplementary Table 3. The ^{1}H (600 MHz) and ^{13}C NMR (150 MHz) Data of 2 in MeOD-d_4

	no	2a		2b	
		δ_C, Type	δ_H (J in Hz)	δ_C, Type	δ_H (J in Hz)
Met(O)	1	169.0, C		169.0, C	
	2	53.1, CH	4.14, t (6.9)	53.1, CH	4.11, t (6.5)
	3	26.6, CH_2	2.39, m[a]	25.4, CH_2	2.39, m[a]
	4	49.1, CH_2	3.06, t (7.4)	48.6, CH_2	3.06, t (7.4)
	5	38.4, CH_3	2.71, s	37.8, CH_3	2.69, s
Asn	1	174.2, C		174.1, C	
	2	50.0, CH	5.09, t (6.8)	49.8, CH	5.07, t (6.8)
	3a	37.3, CH_2	2.86, dd (2.8, 6.8)	37.3, CH_2	2.82, dd (2.8, 6.8)
	3b		2.63, dd (2.8, 6.8)		2.61, dd (2.8, 6.8)
	4	171.4, C		171.3, C	
Pro1	1	174.2, C		174.2, C	
	2	61.9, CH	4.54, dd (3.8, 6.7)	61.9, CH	4.53, dd (3.8, 6.7)
	3	30.9, CH_2	2.06, m[a]	30.8, CH_2	2.03, m[a]
	4	25.6, CH_2	1.98, m	25.4, CH_2	1.98, m
	5a	48.7, CH_2	3.89, m[a]	48.6, CH_2	3.89, m[a]
	5b		3.79, m[a]		3.79, m[a]
Val1	1	173.3, C		173.2, C	
	2	59.7, CH	4.38, d (5.8)	59.7, CH	4.36, d (4.9)
	3	31.9, CH	2.13, m[a]	31.9, CH	2.13, m[a]
	4	20.0, CH_3	0.96, d (4.0)	20.0, CH_3	0.95, d (4.0)
	5	19.5, CH_3	0.95, d (4.0)	19.5, CH_3	0.94, d (4.0)
Phe	1	172.6, C		172.6, C	
	2	54.9, CH	4.70, dd (6.8, 8.8)	54.8, CH	4.69, dd (6.8, 7.6)
	3a	38.9, CH_2	3.06, m[a]	38.8, CH_2	3.06, m[a]
	3b		2.99, dd (5.6, 8.8)		2.97, dd (4.7, 7.6)
	4	137.6, C		137.5, C	
	5/9	130.6, CH	7.24, m[a]	130.6, CH	7.24, m[a]
	6/8	129.6, CH	7.29, t (7.0)	129.6, CH	7.29, t (7.0)
	7	128.3, CH	7.24, m[a]	128.3, CH	7.24, m[a]
Pro2	1	173.9, C		173.9, C	
	2	62.2, CH	4.20, t (3.3)	62.2, CH	4.18, t (3.2)
	3	30.0, CH_2	1.65, m[a]	30.0, CH_2	1.65, m[a]
	4	24.9, CH_2	1.32, m[a]	24.9, CH_2	1.32, m[a]
	5a	48.4, CH_2	3.53, m	48.4, CH_2	3.53, m
	5b		3.31, m[a]		3.31, m[a]
Trp	1	174.2, C		174.2, C	
	2	55.9, CH	4.67, m[a]	55.8, CH	4.67, m[a]
	3a	28.2, CH_2	3.32, m[a]	28.1, CH_2	3.32, m[a]
	3b		3.24, m[a]		3.24, m[a]
	4	111.0, C		111.0, C	
	5	128.7, C		128.6, C	
	6	119.3, CH	7.62, d (7.9)	119.3, CH	7.61, d (7.9)
	7	119.9, CH	7.01, t (7.9)	119.9, CH	7.01, t (7.9)
	8	122.5, CH	7.09, t (7.9)	122.5, CH	7.09, t (7.9)
	9	112.5, CH	7.33, d (7.9)	112.5, CH	7.33, d (7.9)
	10	138.0, C		138.0, C	
	11	124.8, CH	7.15, s	124.8, CH	7.14, s
Val2	1	174.2, C		174.2, C	
	2	61.0, CH	4.04, d (7.6)	61.0, CH	4.04, d (7.6)
	3	31.2, CH	2.13, m[a]	31.2, CH	2.13, m[a]
	4	19.1, CH_3	0.85, d (6.7)	19.0, CH_3	0.85, d (6.7)
	5	19.0, CH_3	0.83, d (6.7)	18.9, CH_3	0.83, d (6.7)
Ala1	1	175.1, C		175.1, C	
	2	51.0, CH	4.33, m[a]	51.0, CH	4.33, m[a]
	3	17.5, CH_3	1.42, d (7.3)	17.5, CH_3	1.42, d (7.3)
Ala2	1	175.8, C		175.8, C	
	2	51.0, CH	4.33, m[a]	51.0, CH	4.33, m[a]

	3	17.3, CH_3	1.44, d (7.1)	17.3, CH_3	1.44, d (7.1)
Ser	1	172.6, C		172.6, C	
	2	57.7, CH	4.33, m[a]	57.7, CH	4.33, m[a]
	3a	62.7, CH_2	3.93, dd (4.8, 11.4)	62.7, CH_2	3.93, dd (4.8, 11.4)
	3b		3.81, dd (4.8, 11.4)		3.81, dd (4.8, 11.4)
Ala3	1	175.1, C		175.1, C	
	2	51.1, CH	4.37, m[a]	51.1, CH	4.37, m[a]
	3	17.8, CH_3	1.40, d (7.2)	17.8, CH_3	1.40, d (7.2)
putrescine	1a	39.3, CH_2	3.32, m[a]	39.3, CH_2	3.32, m[a]
	1b		3.18, t (6.4)		3.16, t (6.2)
	2	27.1, CH_2	1.59, m[a]	27.1, CH_2	1.59, m[a]
	3	25.6, CH_2	1.65, m[a]	25.6, CH_2	1.65, m[a]
	4	40.4, CH_2	2.93, t (6.3)	40.4, CH_2	2.93, t (6.3)

[a] overlapped

Supplementary Table 4. The ^{1}H and ^{13}C NMR Data of 4/6 in MeOD-d_4 and DMSO-d_6

	no	**4**[b]		**6**[c]	
		δ_C, Type	δ_H (J in Hz)	δ_C, Type	δ_H (J in Hz)
Formic acid/	1	164.0, CH	8.15, s	172.3, C	
Decanoic	2			35.2, CH_2	2.10, t (7.3)
acid	3			25.2, CH_2	1.48, m[a]
	4			28.7, CH_2	1.22, m[a]
	5			28.7, CH_2	1.22, m[a]
	6			28.8, CH_2	1.22, m[a]
	7			28.9, CH_2	1.22, m[a]
	8			31.3, CH_2	1.22, m[a]
	9			22.1, CH_2	1.22, m[a]
	10			14.0, CH_3	0.84, t (7.1)
Met	1	172.8, C		171.1, C	
	2	52.5, CH	4.61, dd (5.0, 9.0)	51.4, CH	4.42, m[a]
	3a	32.7, CH_2	2.09, m[a]		1.88, m[a]
	3b		1.99, m[a]	31.9, CH_2	1.73, m[a]
	4	31.1, CH_2	2.58, m[a]	29.7, CH_2	2.39, m
	5	15.4, CH_3	2.09, s	14.7, CH_3	1.99, s
	2-NH				7.91, d (8.1)
Asn	1	174.3, C		172.0, C	
	2	50.1, CH	4.97, t (7.0)	47.5, CH	4.80, dd (5.6, 8.7)
	3a	37.8, CH_2	2.87, dd (8.1, 15.3)	37.1, CH_2	2.63, dd (8.7, 15.4)
	3b		2.58, m[a]		2.27, dd (5.6, 15.4)
	4	171.7, C		170.0, C	
	2-NH				8.16, d (7.7)
Pro1	1	174.3, C		171.1, C	
	2	62.1, CH	4.54, dd (3.9, 7.8)	59.6, CH	4.36, m[a]
	3a	30.7, CH_2	2.15, m[a]	28.7, CH_2	1.83, m[a]
	3b		2.05, m[a]		
	4	25.9, CH_2	1.99, m[a]	24.2, CH_2	1.72, m[a]
	5a	48.7, CH_2	3.84, m[a]	46.8, CH_2	3.67, m[a]
	5b		3.76, m		3.61, m[a]
Val1	1	173.4, C		170.6, C	
	2	59.8, CH	4.39, d (7.3)	57.1, CH	4.42, m[a]
	3	32.0, CH	2.15, m[a]	31.0, CH	1.88, m[a]
	4	20.1, CH_3	0.99, d (7.1)	19.1, CH_3	0.76, d (6.6)
	5	19.5, CH_3	0.97, d (7.0)	18.2, CH_3	0.72, d (6.6)
	2-NH				7.68, d (8.7)
Phe	1	172.6, C		169.8, C	
	2	55.0, CH	4.69, dd (6.6, 8.9)	52.7, CH	4.72, m
	3a	38.9, CH_2	3.12, dd (6.6, 13.1)	37.9, CH_2	2.97, m[a]
	3b		3.02, dd (8.9, 13.1)		2.88, m[a]
	4	137.6, C		136.9, C	
	5/9	130.6, CH	7.26, m[a]	129.3, CH	7.20, m[a]
	6/8	129.6, CH	7.31, m[a]	128.2, CH	7.25, m[a]
	7	128.2, CH	7.26, m[a]	126.6, CH	7.20, m[a]
	2-NH				8.37, d (6.8)
Pro2	1	173.9, C		171.2, C	
	2	62.3, CH	4.20, dd (4.0, 7.8)	60.1, CH	4.12, dd (3.7, 7.7)
	3	30.0, CH_2	1.67, m[a]	28.7, CH_2	1.70, m[a]
	4	24.9, CH_2	1.39, m[a]	23.8, CH_2	1.42, m[a]
	5a	48.4, CH_2	3.55, m	46.8, CH_2	3.31, m[a]
	5b		3.38, m[a]		2.86, m[a]

Trp	1	174.2, C		171.5, C	
	2	56.0, CH	4.65, dd (7.1, 8.1)	53.7, CH	4.53, dd (5.3, 8.4)
	3a	28.1, CH_2	3.34, m[a]	27.0, CH_2	3.18, dd (5.3, 14.7)
	3b		3.26, m[a]		2.97, m[a]
	4	110.9, C		110.2, C	
	5	128.6, C		127.2, C	
	6	119.2, CH	7.62, d (7.8)	118.2, CH	7.55, d (7.8)
	7	119.9, CH	7.03, t (7.8)	118.3, CH	6.95, t (7.8)
	8	122.6, CH	7.11, t (7.8)	120.8, CH	7.04, t (7.8)
	9	112.5, CH	7.35, t (7.8)	111.3, CH	7.29, d (7.8)
	10	138.1, C		136.1, C	
	11	124.9, CH	7.16, s	123.5, CH	7.12, s
	2-NH				7.90, d (6.7)
	11-NH				10.7, s
Val2	1	174.2, C		171.3, C	
	2	61.1, CH	4.04, d (7.7)	58.2, CH	4.09, m
	3	31.1, CH	2.10, m[a]	29.8, CH	1.99, m[a]
	4	19.1, CH_3	0.85, d (5.7)	18.8, CH_3	0.76, d (6.6)
	5	19.0, CH_3	0.86, d (5.9)	18.1, CH_3	0.73, d (6.6)
	2-NH				7.74, d (8.3)
Ala1	1	175.1, C		172.5, C	
	2	51.0, CH	4.34, m[a]	48.3, CH	4.42, m[a]
	3	17.4, CH_3	1.44, d (7.4)	17.8, CH_3	1.20, d[a] (7.3)
	2-NH				8.29, d (7.1)
Ala2	1	175.9, C		172.7, C	
	2	51.1, CH	4.34, m[a]	48.5, CH	4.29, m
	3	17.2, CH_3	1.46, d (7.3)	17.7, CH_3	1.23, d (7.3)
	2-NH				8.04, d (6.8)
Ser	1	172.6, C		169.7, C	
	2	57.8, CH	4.34, m[a]	55.0, CH	4.25, m
	3a	62.7, CH_2	3.96, dd (5.2, 11.4)	61.6, CH_2	3.63, dd (5.7, 10.6)
	3b		3.84, dd (4.5, 11.4)		3.54, m[a]
	2-NH				8.10, d (7.3)
Ala3	1	175.1, C		172.0, C	
	2	51.1, CH	4.40, m[a]	48.5, CH	4.20, m
	3	17.8, CH_3	1.42, d (7.4)	17.7, CH_3	1.20, d[a] (7.3)
	2-NH				8.05, d (6.8)
putrescine	1a	39.3, CH_2	3.34, m[a]	37.9, CH_2	3.08, m
	1b		3.21, m[a]		2.97, m[a]
	2	27.1, CH_2	1.62, m[a]	25.9, CH_2	1.42, m[a]
	3a	25.6, CH_2	1.67, m[a]	24.3, CH_2	1.48, m[a]
	3b		1.62, m[a]		
	4	40.4, CH_2	2.95, t (6.9)	38.6, CH_2	2.76, m
	1-NH				7.66, t (4.6)
	4-NH				7.64, brs

[a] overlapped
[b] signals were recorded in MeOD-d_4 solvent on 500M NMR spectrometer
[c] signals were recorded in DMSO-d_6 solvent on 600M NMR spectrometer

Supplementary Table 5. The ^{1}H and ^{13}C NMR Data of 7/8 in MeOD-d_4 and DMSO-d_6

	no	**7**[b]		**8**[c]	
		δ_C, Type	δ_H (J in Hz)	δ_C, Type	δ_H (J in Hz)
Lauric acid	1	176.4, C		172.2, C	
	2	37.0, CH_2	2.25, m[a]	35.2, CH_2	2.10, t (7.1)
	3	26.9, CH_2	1.64, m[a]	25.2, CH_2	1.48, m[a]
	4	30.4, CH_2	1.29, m[a]	28.7, CH_2	1.22, m[a]
	5	30.4, CH_2	1.29, m[a]	28.7, CH_2	1.22, m[a]
	6	30.7, CH_2	1.29, m[a]	28.8, CH_2	1.22, m[a]
	7	30.7, CH_2	1.29, m[a]	29.0, CH_2	1.22, m[a]
	8	30.6, CH_2	1.29, m[a]	29.0, CH_2	1.22, m[a]
	9	30.5, CH_2	1.29, m[a]	29.0, CH_2	1.22, m[a]
	10	33.0, CH_2	1.29, m[a]	31.3, CH_2	1.22, m[a]
	11	23.7, CH_2	1.29, m[a]	22.1, CH_2	1.22, m[a]
	12	14.4, CH_3	0.90, t (7.0)	14.0, CH_3	0.85, t (6.9)
Met/ Met(O)	1	173.9, C		170.5, C	
	2	53.7, CH	4.53, dd (4.4, 8.8)	50.9, CH	4.44, m[a]
	3a	32.7, CH_2	2.11, m[a]	25.1, CH_2	1.91, m[a]
	3b		1.96, m[a]		
	4	31.3, CH_2	2.56, m[a]	49.2, CH_2	2.71, m
			2.50, m		2.63, m[a]
	5	15.4, CH_3	2.07, s	38.0, CH_3	2.51, s[a]
	2-NH				7.95, d (8.1)
Asn	1	174.8, C		172.0, C	
	2	50.0, CH	4.95, dd (6.1, 8.4)	47.4, CH	4.84, m
	3a	37.8, CH_2	2.85, dd (8.4, 15.2)	37.1, CH_2	2.63, m[a]
	3b		2.56, m[a]		2.28, m
	4	171.7, C		159.9, C	
	2-NH				8.35, d (7.0)
Pro1	1	174.3, C		171.1, C	
	2	62.2, CH	4.53, dd (4.4, 8.8)	59.5, CH	4.34, m[a]
	3a	30.7, CH_2	2.15, m[a]	28.7, CH_2	1.85, m[a]
	3b		2.11, m[a]		
	4a	25.8, CH_2	2.03, m[a]	24.2, CH_2	1.74, m[a]
	4b		1.96, m		
	5	48.7, CH_2	3.82, m[a]	46.8, CH_2	3.73, m[a]
			3.74, t (7.2)		3.63, m[a]
Val1	1	173.2, C		170.6, C	
	2	59.8, CH	4.41, d (7.1)	57.2, CH	4.34, m[a]
	3	32.1, CH	2.17, m[a]	30.8, CH	1.92, m[a]
	4	20.1, CH_3	0.97, d (5.0)	19.1, CH_3	0.75, d (6.5)
	5	19.5, CH_3	0.96, d (5.0)	18.2, CH_3	0.71, d (6.5)
	2-NH				7.69, d (8.9)
Phe	1	172.7, C		169.7, C	
	2	54.9, CH	4.66, m[a]	52.5, CH	4.73, m
	3a	39.0, CH_2	3.10, dd (6.2, 13.0)	37.7, CH_2	2.98, m[a]
	3b		2.98, dd (9.0, 13.0)		2.90, m[a]
	4	137.6, C		136.9, C	
	5/9	130.6, CH	7.24, m[a]	129.3, CH	7.21, m[a]
	6/8	129.6, CH	7.28, m[a]	128.2, CH	7.25, m[a]
	7	128.2, CH	7.24, m[a]	126.6, CH	7.21, m[a]
	2-NH				8.34, d (6.9)
Pro2	1	173.8, C		171.3, C	
	2	62.2, CH	4.17, dd (4.0, 8.3)	60.0, CH	4.15, m
	3	30.0, CH_2	1.64, m[a]	28.7, CH_2	1.73, m[a]
	4a	24.9, CH_2	1.38, m[a]	23.8, CH_2	1.42, m[a]
	4b		1.30, m[a]		
	5a	48.4, CH_2	3.53, m	46.8, CH_2	3.33, m[a]
	5b		3.32, m[a]		2.91, m[a]

Trp	1	174.2, C		171.6, C	
	2	55.9, CH	4.65, m[a]	53.7, CH	4.55, m
	3a	28.0, CH_2	3.32, m[a]	27.1, CH_2	3.17, m
	3b		3.24, dd (6.0, 14.8)		2.96, m[a]
	4	110.8, C		110.1, C	
	5	128.6, C		127.2, C	
	6	119.2, CH	7.60, d (7.9)	118.2, CH	7.56, d (7.9)
	7	119.9, CH	7.01, t (7.9)	118.3, CH	6.95, t (7.9)
	8	122.6, CH	7.09, t (7.9)	120.9, CH	7.03, t (7.8)
	9	112.5, CH	7.33, d (8.0)	111.3, CH	7.30, d (7.8)
	10	138.0, C		136.1, C	
	11	124.8, CH	7.13, s	123.6, CH	7.14, s
	2-NH				7.92, d (7.4)
	11-NH				10.7, s
Val2	1	174.1, C		171.1, C	
	2	61.0, CH	4.04, d (7.8)	58.2, CH	4.07, m
	3	31.1, CH	2.14, m[a]	29.8, CH	1.99, m
	4	19.2, CH_3	0.85, d (7.1)	18.8, CH_3	0.75, d (6.5)
	5	19.0, CH_3	0.83, d (7.1)	18.0, CH_3	0.71, d (6.5)
	2-NH				7.78, d (7.7)
Ala1	1	175.0, C		172.3, C	
	2	50.9, CH	4.32, m[a]	48.4, CH	4.38, m
	3	17.4, CH_3	1.42, d (7.1)	17.8, CH_3	1.20, d[a] (7.0)
	2-NH				8.27, d (7.2)
Ala2	1	175.9, C		172.8, C	
	2	51.1, CH	4.32, m[a]	48.5, CH	4.28, m
	3	17.3, CH_3	1.44, d (7.2)	17.7, CH_3	1.21, d (7.0)
	2-NH				8.04, d[a] (7.4)
Ser	1	172.6, C		169.7, C	
	2	57.8, CH	4.32, m[a]	55.0, CH	4.24, m
	3a	62.7, CH_2	3.93, dd (4.6, 11.0)	61.6, CH_2	3.63, m
	3b		3.82, dd (4.6, 11.0)		3.54, m[a]
	2-NH				8.09, d (7.1)
Ala3	1	175.0, C		172.0, C	
	2	51.1, CH	4.41, m[a]	48.5, CH	4.21, m
	3	17.9, CH_3	1.40, d (7.3)	17.7, CH_3	1.20, d[a] (7.0)
	2-NH				8.04, d[a] (7.4)
putrescine	1a	39.3, CH_2	3.32, m[a]	37.9, CH_2	3.09, m
	1b		3.18, m[a]		2.98, m[a]
	2	27.1, CH_2	1.64, m[a]	25.9, CH_2	1.42, m[a]
	3	25.5, CH_2	1.64, m[a]	24.4, CH_2	1.48, m[a]
	4	40.4, CH_2	2.93, t (7.1)	38.6, CH_2	2.77, m
	2-NH				7.67, t (5.5)
	4-NH				7.64, brs

[a] overlapped
[b] signals were recorded in MeOD-d_4 solvent on 600M NMR spectrometer
[c] signals were recorded in DMSO-d_6 solvent on 600M NMR spectrometer

Supplementary Table 6. The ^{1}H (600 MHz) and ^{13}C (150 MHz) NMR Data of 9/10 in DMSO-d_6

	no	9		10	
		δ_C, Type	δ_H (J in Hz)	δ_C, Type	δ_H (J in Hz)
Dodecanoic	1	176.4, C		176.4, C	
Acid (DA)	2	37.0, CH_2	2.25, t (7.4)	37.0, CH_2	2.25, m[a]
	3a	27.0, CH_2	1.66, m[a]	26.9, CH_2	1.64, m[a]
	3b		1.60, m[a]		
Formic acid/	4	30.3, CH_2	1.29, m[a]	30.4, CH_2	1.28, m[a]
Lauric acid	5	30.4, CH_2	1.29, m[a]	30.5, CH_2	1.28, m[a]
	6	30.7, CH_2	1.29, m[a]	30.7, CH_2	1.28, m[a]
	7	30.7, CH_2	1.29, m[a]	30.7, CH_2	1.28, m[a]
	8	30.7, CH_2	1.29, m[a]	30.7, CH_2	1.28, m[a]
	9	30.4, CH_2	1.29, m[a]	30.7, CH_2	1.28, m[a]
	10	33.0, CH_2	1.29, m[a]	30.6, CH_2	1.28, m[a]
	11	23.7, CH_2	1.29, m[a]	30.5, CH_2	1.28, m[a]
	12	14.4, CH_3	0.92, t (7.2)	33.1, CH_2	1.28, m[a]
	13			23.7, CH_2	1.28, m[a]
	14			14.4, CH_3	0.90, t (6.8)
Leu /Met	1	174.9, C		173.9, C	
	2	52.9, CH	4.46, dd (4.7, 10.7)	53.7, CH	4.53, dd (4.4, 8.7)
	3a	41.7, CH_2	1.66, m[a]	32.7, CH_2	2.12, m[a]
	3b		1.60, m[a]		1.97, m[a]
	4	26.0, CH	1.66, m[a]	31.4, CH_2	2.56, m[a]
					2.50, m
	5	23.7, CH_3	0.96, d (6.5)	15.5, CH_3	2.08, s
	6	21.8, CH_3	0.93, d (6.5)		
Asn	1	174.8, C		174.8, C	
	2	50.0, CH	4.94, dd (6.0, 8.0)	50.0, CH	4.95, dd (6.1, 8.4)
	3a	37.9, CH_2	2.86, dd (8.0, 15.2)	37.9, CH_2	2.85, dd (8.4, 15.3)
	3b		2.54, dd (6.0, 15.2)		2.56, m[a]
	4	171.6, C		171.7, C	
Pro1	1	174.4, C		174.3, C	
	2	62.3, CH	4.53, dd (4.1, 8.0)	62.3, CH	4.53, dd (4.3, 8.7)
	3a	30.7, CH_2	2.15, m[a]	30.8, CH_2	2.16, m[a]
	3b		2.03, m[a]		2.11, m[a]
	4a	25.9, CH_2	2.03, m[a]	25.8, CH_2	2.03, m[a]
	4b		1.99, m		1.96, m[a]
	5a	48.9, CH_2	3.75, t (5.6)	48.7, CH_2	3.82, m[a]
	5b				3.75, m
Val1	1	173.3, C		173.2, C	
	2	59.8, CH	4.41, d (7.4)	59.8, CH	4.41, m[a]
	3	32.2, CH	2.17, m[a]	32.1, CH	2.16, m[a]
	4	20.1, CH_3	0.98, d (5.2)	20.1, CH_3	0.97, d (4.6)
	5	19.5, CH_3	0.97, d (5.2)	19.5, CH_3	0.96, d (4.6)
Phe	1	172.7, C		172.7, C	
	2	55.0, CH	4.65, t (6.1)	55.0, CH	4.66, m[a]
	3a	38.9, CH_2	3.18, dd (6.1, 12.8)	39.0, CH_2	3.10, dd (6.2, 13.0)
	3b		3.10, dd (6.1, 12.8)		2.98, dd (9.2, 13.0)
	4	137.6, C		137.6, C	
	5/9	130.6, CH	7.24, m[a]	130.6, CH	7.24, m[a]
	6/8	129.6, CH	7.29, m[a]	129.6, CH	7.28, m[a]
	7	128.2, CH	7.24, m[a]	128.2, CH	7.24, m[a]
Pro2	1	173.9, C		173.8, C	
	2	62.1, CH	4.18, dd (4.0, 8.0)	62.2, CH	4.17, dd (4.2, 8.8)
	3	30.0, CH_2	1.66, m[a]	30.0, CH_2	1.64, m[a]
	4a	24.9, CH_2	1.38, m[a]	25.0, CH_2	1.38, m[a]
	4b		1.31, m[a]		1.30, m[a]
	5a	48.3, CH_2	3.53, m	48.4, CH_2	3.53, m
	5b		3.34, m[a]		3.32, m[a]
Trp	1	174.3, C		174.2, C	
	2	55.9, CH	4.65, dd (6.5, 8.7)	55.9, CH	4.65, m[a]
	3a	28.1, CH_2	3.31, m[a]	28.0, CH_2	3.32, m[a]
	3b		3.25, dd (8.7, 14.0)		3.24, dd (5.8, 14.3)
	4	110.8, C		110.8, C	

	5	128.6, C		128.6, C	
	6	119.2, CH	7.60, d (7.9)	119.2, CH	7.60, d (7.9)
	7	119.9, CH	7.01, t (7.9)	119.9, CH	7.01, t (7.9)
	8	122.5, CH	7.09, t (7.9)	122.6, CH	7.09, t (7.9)
	9	112.5, CH	7.34, d (7.9)	112.5, CH	7.33, d (7.9)
	10	138.1, C		138.1, C	
	11	124.9, CH	7.14, s	124.8, CH	7.13, s
Val2	1	174.1, C		174.1, C	
	2	61.0, CH	4.04, d (7.7)	61.0, CH	4.04, d (7.8)
	3	31.1, CH	2.15, m[a]	31.1, CH	2.16, m[a]
	4	19.2, CH_3	0.83, d (7.1)	19.2, CH_3	0.85, d (6.8)
	5	19.0, CH_3	0.85, d (7.1)	19.0, CH_3	0.84, d (6.8)
Ala1	1	175.1, C		175.0, C	
	2	51.0, CH	4.32, m[a]	60.0, CH	4.32, m[a]
	3	17.4, CH_3	1.42, d (7.4)	17.4, CH_3	1.42, d (7.3)
Ala2	1	175.9, C		175.9, C	
	2	51.1, CH	4.32, m[a]	51.1, CH	4.32, m[a]
	3	17.2, CH_3	1.44, d (7.1)	17.3, CH_3	1.44, d (7.1)
Ser	1	172.6, C		172.6, C	
	2	57.8, CH	4.32, m[a]	57.8, CH	4.32, m[a]
	3a	62.7, CH_2	3.94, dd (5.2, 11.4)	62.7, CH_2	3.93, dd (5.2, 11.0)
	3b		3.82, dd (4.6, 11.4)		3.82, dd (4.6, 11.0)
Ala3	1	175.1, C		175.0, C	
	2	51.1, CH	4.40, m[a]	51.1, CH	4.41, m[a]
	3	17.8, CH_3	1.40, d (7.1)	17.9, CH_3	1.40, d (7.2)
putrescine	1a	39.3, CH_2	3.32, m[a]	39.3, CH_2	3.32, m[a]
	1b		3.18, dd (7.1, 13.6)		3.18, m[a]
	2	27.1, CH_2	1.60, m[a]	27.1, CH_2	1.64, m[a]
	3a	25.5, CH_2	1.66, m[a]	25.5, CH_2	1.64, m[a]
	3b		1.60, m[a]		
	4	40.4, CH_2	2.93, t (7.1)	40.4, CH_2	2.93, t (7.6)

[a] overlapped

Supplementary Table 7. The ^{1}H (600 MHz) and ^{13}C NMR (150 MHz) Data of 29 in DMSO-d_6

	no	δ_C, Type	δ_H	Mult. (J in Hz)
Octanoic acid	1	172.5, C		
	2	35.1, CH_2	2.10	m
	3	25.3, CH_2	1.47	m[a]
	4	28.5, CH_2	1.23	m[a]
	5	28.4, CH_2	1.23	m[a]
	6	31.2, CH_2	1.23	m[a]
	7	22.0, CH_2	1.23	m[a]
	8	13.9, CH_3	0.85	t (7.1)
Leu	1	172.3, C		
	2	50.8, CH	4.39	m
	3	39.9, CH_2	1.47	m[a]
	4	24.1, CH	1.60	m
	5	23.2, CH_3	0.90	d (6.7)
	6	21.4, CH_3	0.86	d (6.7)
	NH		8.02	d (8.2)
Thr	1	170.7, C		
	2	58.5, CH	4.03	dd (3.9, 8.2)
	3	66.8, CH	3.91	m
	4	19.4, CH_3	0.86	d (6.7)
	NH		7.71	d (8.2)
Tyr	1	171.2, C		
	2	54.8, CH	4.34	m[a]
	3a	36.0, CH_2	2.98	dd (3.3, 14.0),
	3b		2.66	dd (10.6, 14.0)
	4	127.0, C		
	5/9	130.0, CH	7.02	d (8.5)
	6/8	115.0, CH	6.60	d (8.5)
	7	155.9, C		
	NH		7.99	d (7.0)
	OH		9.17	brs
Ala1	1	171.9, C		
	2	48.9, CH	4.22	m[a]
	3	17.7, CH_3	1.23	d (7.0)
	NH		8.08	d (6.8)
Ala2	1	172.0, C		
	2	48.3, CH	4.22	m[a]
	3	18.1, CH_3	1.20	d (7.1)
	NH		7.92	d (7.4)
Ala3	1	172.1, C		
	2	48.4, CH	4.34	m[a]
	3	18.5, CH_3	1.21	d (7.1)
	NH		7.98	d (6.8)
Val	1	170.7, C		
	2	57.7, CH	4.08	dd (7.0, 8.9)
	3	30.4, CH	1.97	m
	4	19.2, CH_3	0.82	d (6.8)
	5	18.0, CH_3	0.81	d (6.8)
	NH		7.88	d (9.0)
putrescine	1a	37.9, CH_2	3.11	m
	1b		3.03	m
	2	26.0, CH_2	1.44	m[a]
	3	24.5, CH_2	1.50	m
	4	38.6, CH_2	2.77	m
	1-NH		8.00	t (4.6)
	4-NH		7.68	brs

[a]overlapped

Supplementary Table 8. The ^{1}H (600 MHz) and ^{13}C NMR (150 MHz) Data of 31 in DMSO-d_6

	no	δ_C, Type	δ_H	Mult. (J in Hz)
Octanoic acid	1	172.3, C		
	2a	35.2, CH_2	2.13	m
	2b		2.07	m
	3	25.4, CH_2	1.43	m^a
	4	28.6, CH_2	1.22	m^a
	5	28.4, CH_2	1.22	m^a
	6	31.2, CH_2	1.22	m^a
	7	22.1, CH_2	1.22	m^a
	8	13.9, CH_3	0.85	t (7.0)
Val	1	171.0, C		
	2	57.7, CH	4.09	dd (7.2, 8.8)
	3	30.3, CH	1.88	m
	4	19.2, CH_3	0.74	d (6.4)
	5	18.1, CH_3	0.73	d (6.4)
	2-NH		7.71	d (8.9)
Phe	1	171.0, C		
	2	53.5, CH	4.56	m
	3a	37.4, CH_2	3.03	dd (4.3, 14.5)
	3b		2.79	dd (9.8, 14.5)
	4	137.7, C		
	5/9	129.2, CH	7.23	m^a
	6/8	128.0, CH	7.23	m^a
	7	126.2, CH	7.16	t (6.9)
	2-NH		7.92	d (8.3)
Glu	1	171.6, C		
	2	51.6, CH	4.20	m^a
	3a	27.3, CH_2	1.96	m
	3b		1.80	m
	4a	31.5, CH_2	2.26	m
	4b		2.19	m
	5	173.1, C		
	2-NH		8.17	d (7.7)
	5-COOH		12.66	brs
Ile	1	170.7, C		
	2	56.9, CH	4.15	t (8.0)
	3	36.5, CH	1.70	m
	4a	24.4, CH_2	1.39	m
	4b		1.09	m
	5	11.1, CH_3	0.80	t (7.5)
	6	15.3, CH_3	0.82	d (6.8)
	2-NH		7.87	d (8.4)
Ala	1	172.0, C		
	2	48.2, CH	4.20	m^a
	3	18.3, CH_3	1.19	d (7.1)
	2-NH		7.96	d (7.3)
putrescine	1a	37.8, CH_2	3.09	m
	1b		3.01	m
	2	26.0, CH_2	1.43	m^a
	3	24.3, CH_2	1.50	m
	4	38.5, CH_2	2.77	m
	1-NH		7.80	t (5.7)
	4-NH		7.69	brs

^{a}overlapped

Supplementary Table 9. The specificity-conferring code of A domains

Domain	Position										Predicted amino acid	Found
	235	236	239	278	299	301	322	330	331	517		
A1	D	A	F	W	L	G	G	T	F	K	Phe	Leu/Met/ Met(O)
A2	D	A	T	H	V	G	E	V	C	K	Asn	Asn
A3	D	V	Q	F	P	L	H	V	V	K	Pro	Pro
A4	D	A	F	W	L	G	G	T	F	K	Val	Val
A5	D	A	W	T	L	V	A	V	C	K	Phe	Phe
A6	D	V	Q	F	P	L	K	V	T	K	Pro	Pro
A7	D	V	Q	H	E	M	Q	V	S	K	X	Trp
A8	D	A	F	W	L	G	G	T	F	K	Val	Val
A9	D	L	F	A	G	A	N	T	T	K	Ala	Ala
A10	D	L	F	A	G	A	N	T	T	K	Ala	Ala
A11	D	V	W	H	M	S	L	V	D	K	Ser	Ser
A12	D	L	F	A	G	A	N	T	T	K	Ala	Ala
A*	-	-	-	-	-	-	-	-	-	-	-	-

“X” indicates unknown amino acid.

Supplementary Table 10. Retention times of amino acids derivatized with Marfey's reagent (L-FDAA)

Amino acid	configuration	Retention time of hydrolysed compounds						
		standard amino acid	1	2	4	7	9	10
Ala	D	24.2	24.2	24.2	24.2	24.2	24.2	24.2
	L	21.6	21.6	21.6	21.6	21.6	21.6	21.6
Ser	D	18.3						
	L	18.0	18.0	18.0	18.0	18.0	18.0	18.0
Val	D	30.1	30.1	30.1	30.1	30.1	30.1	30.1
	L	26.8	26.8	26.8	26.8	26.8	26.8	26.8
Phe	D	32.8	32.8	32.8	32.8	32.8	32.8	32.8
	L	30.0						
Pro	D	23.7						
	L	22.5	22.5	22.5	22.5	22.5	22.5	22.5
Asp	D	20.2	20.2	20.2	20.2	20.2	20.2	20.2
	L	19.0						
Trp	D	31.4						
	L	29.4	29.4	29.4	29.4	29.4	29.4	29.4
Met	D	29.2	29.2		29.2	29.2		29.2
	L	26.0						
Leu	D	33.4					33.4	
	L	30.2						
Met(O)	D	18.0/18.5		18.0/18.5				
	L	18.3						

Supplementary Table 11. IC_{50} values (μM) for glidonins in eight tumor cells and one normal cell

Compound	Kasumi	K562	MDA-MB-231	MCF-7	A549	HepG2	HCT116	SGC-7901	2B
**1**	18.30±1.22	>20	>20	>20	7.20±0.79	>20	>20	>20	>20
**2**	>20	>20	>20	>20	>20	>20	>20	>20	>20
**4**	>20	>20	>20	>20	>20	>20	>20	>20	>20
**6**	-	-	>20	-	>20	>20	-	-	>20
**7**	7.93±0.35	13.51±4.44	4.76±0.79	>20	7.64±1.11	8.30±0.57	>20	>20	11.73±1.50
**8**	-	-	>20	-	>20	>20	-	-	>20
**9**	9.65±0.4	>20	4.26±1.09	>20	3.26±0.45	>20	>20	>20	10.43±0.54
**10**	1.39±6.36	7.20±0.79	7.2±0.79	>20	7.15±0.47	6.68±0.26	>20	>20	11.76±0.15
**10a**	-	-	-	-	>20	>20	-	-	>20
positive control (doxorubicin)	0.42±0.10	0.79±0.34	0.63±0.07	2.6±0.34	0.82±0.25	3.21±0.67	0.59±0.04	0.14±0.63	1.32±0.11

Supplementary Table 12. IC_{50} values (μM) for compounds in eight tumor cells and one normal cell

Compound	MDA-MB-231	A549	SKOV3	HepG-2	2B
**29**	110.4±2.60	39.92±0.15	81.4±1.02	265.5±2.43	102.38±0.25
**29a**	n.s.	82.2±2.58	160.7±3.67	267.5±0.01	269.40±1.23
**31**	168.9±1.05	198.3±1.25	241.4±0.07	136.2±0.15	42.56±1.25
**31a**	200.5±0.07	453.4±0.68	n.s.	94.29±0.19	92.26±0.03
Adriamycin	0.91±0.05	0.73±0.05	1.29±0.01	0.23±0.02	1.34±0.09

n.s. indicates negative significance.

Supplementary Table 13. Three formyltransferases on the genome of DSM 7029

	Accession No.	ORFs	Size
1	AKJ26698.1	Methionyl-tRNA formyltransferase	966 bp
2	AKJ27886.1	Phosphoribosylaminoimidazolecarboxamide formyltransferase (PurH)	1569 bp
3	AKJ28062.1	Phosphoribosylglycinamide formyltransferase (PurN)	627 bp

Supplementary Table 14. The constitutes of the synthesized peptides.

Compounds	$[M + H]^+$	Constitutes
**1a**	1289.6347	D-Met^1-D-Asn^2-L-Pro^3-L-Val^4-D-Phe^5-L-Pro^6-L-Trp^7-D-Val^8-L-Ala^9-D-Ala^{10}-L-Ser^{11}-L-Ala^{12}
**13a**	607.2908	D-Met^1-D-Asn^2-L-Pro^3-L-Val^4-D-Phe^5
**13**	789.4579	lauroyl-D-Met^1-D-Asn^2-L-Pro^3-L-Val^4-D-Phe^5
**14**	789.4579	lauroyl-L-Met^1-D-Asn^2-L-Pro^3-L-Val^4-D-Phe^5
**15**	446.2683	lauroyl-D-Met-D-Asn
**10a**	1499.8331	myristoyl-D-Met^1-D-Asn^2-L-Pro^3-L-Val^4-D-Phe^5-L-Pro^6-L-Trp^7-D-Val^8-L-Ala^9-D-Ala^{10}-L-Ser^{11}-L-Ala^{12}
**29a**	834.4971	octanoyl-L-Leu-L-Thr-L-Tyr-D-Ala-L-Ala-D-Ala-L-Val
**30a**	704.4229	octanoyl-L-Val-L-Phe-L-Glu-L-Ile-L-Ala

Supplementary Table 15. The identity between GdnB-A* and other A domains of NRPSs in *Schlegelella brevitalea* DSM 7029 by the BlastP analysis

Domain	Coverage (aa)	Identities (aa)	Substrate
GdnB-A*/GdnB-A6	190/409	50/111 (45%)	Pro
GdnB-A*/GdnB-A8	190/398	48/106 (45%)	Val
GdnB-A*/GdnB-A7	190/404	49/111 (44%)	Trp
GdnB-A*/GdnB-A12	190/394	47/106 (44%)	Ala
GdnB-A*/GdnB-A4	190/397	47/106 (44%)	Val
GdnB-A*/GdnB-A9	190/394	46/106 (43%)	Ala
GdnB-A*/GdnB-A10	190/394	48/106 (45%)	Ala
GdnB-A*/GdnB-A11	190/413	47/111(42%)	Ser
GdnB-A*/GdnB-A5	190/400	46/111(41%)	Phe

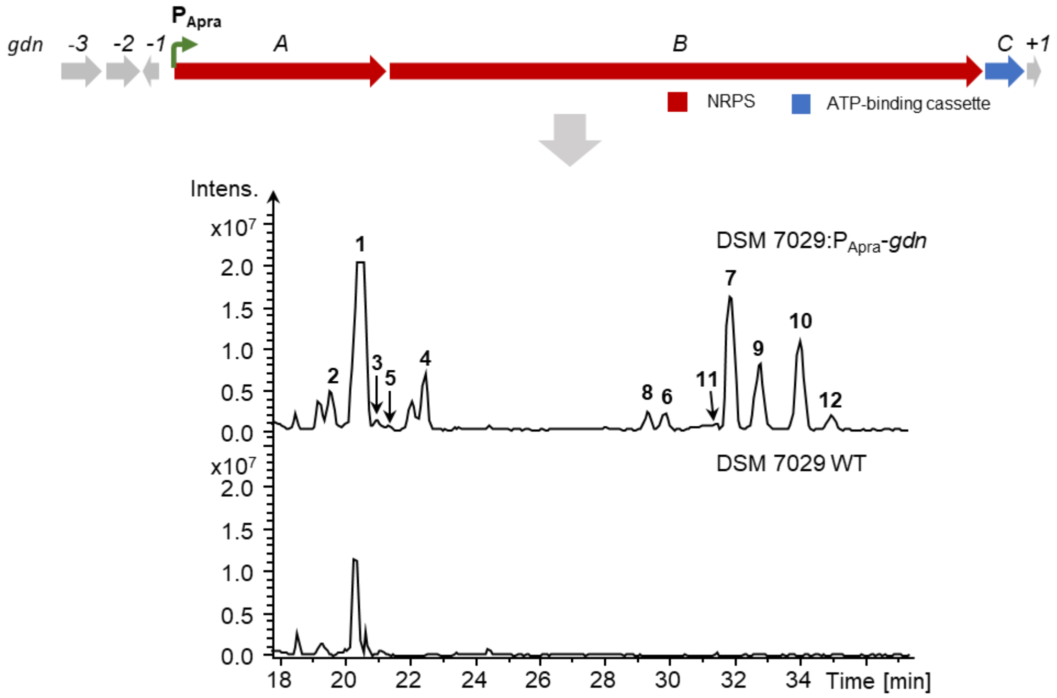

Supplementary Fig. 1. The HPLC-MS profile of the products of glidonin gene cluster in the activated mutant DSM 7029:P_{Apra}-*gdn* and DSM 7029 wild type.

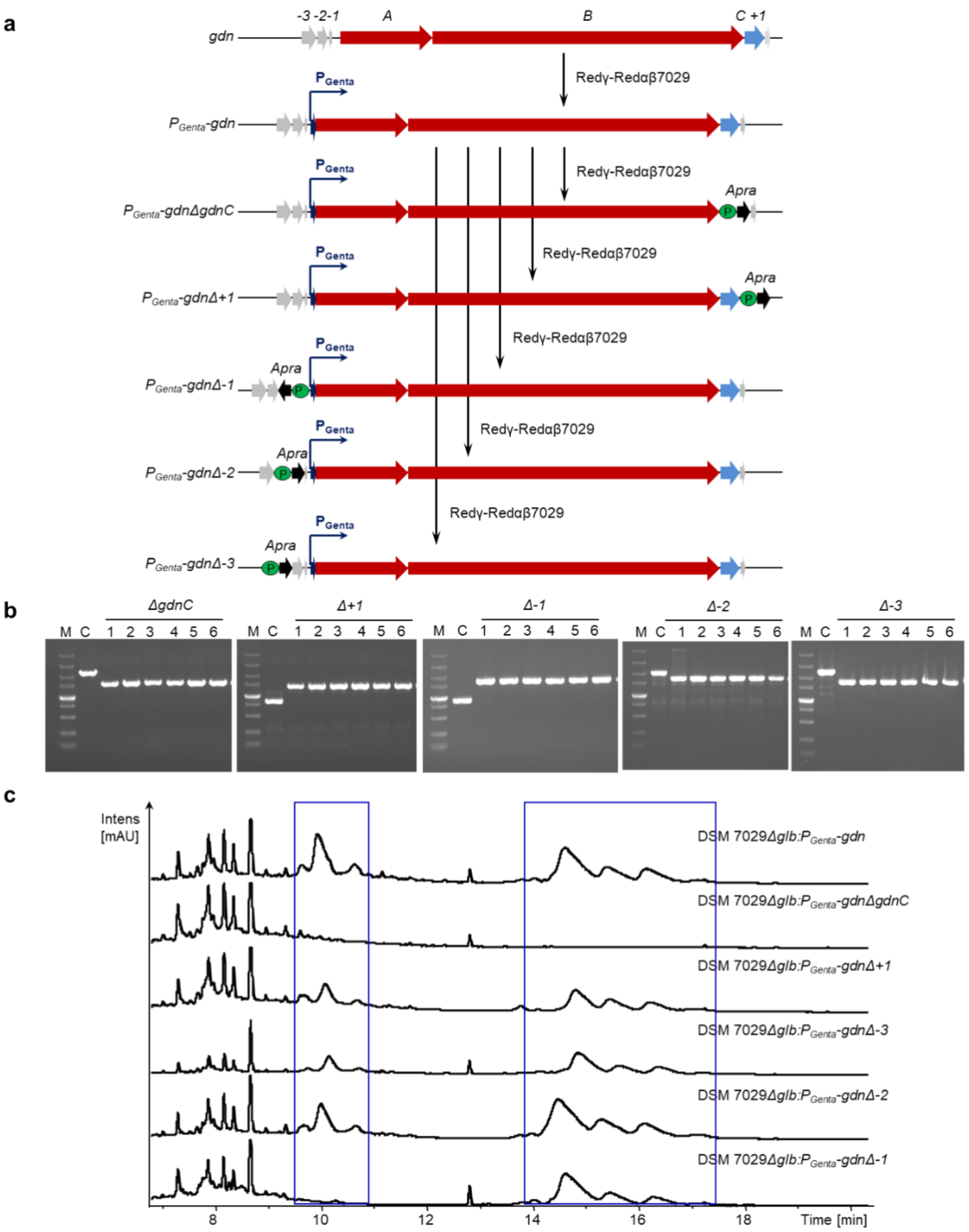

Supplementary Fig. 2. Diagram for construction and verification of deletion of single gene *gdnC*, *+1*, *-1*, *-2*, and *-3* of the glidonin BGC in *S. brevitalea* DSM 7029. **a.** Diagram for construction of deletion of genes using Redγ-Redαβ7029 recombinases. **b.** PCR verification of deletion of genes. The size of PCR product is 2216 bp for negative control, and 1460 bp for correct mutant DSM 7029*ΔgdnC* (Del-gdnC-check-S/Del-gdnC-check-A); The size of PCR product is 831 bp for negative control, and 1285 bp for correct mutant DSM 7029*Δ+1* (Del-(+1)-check-S/Del-(+1)-check-A); The size of PCR product is 894 bp for negative control, and 1591 bp for correct mutant DSM7029*Δ-1* (Del-(-1)-check-S/Del-(-1)-check-A); The size of PCR product is 1953 bp for negative control, and 1573 bp for correct mutant DSM 7029*Δ-2* (Del-(-2)-check-S/Del-(-2)-check-A); The size of PCR product is 2306 bp for negative control, and 1634 bp for correct mutant DSM 7029*Δ-3* (Del-(-3)-check-S/Del-(-3)-check-A). M: DL 5000 DNA ladder, C: DSM 7029P_{Genta}-*gdn*. **c.** Metabolic profiles between DSM 7029P_{Genta}-*gdn* and different mutants through LC-MS analysis. The blue frame indicates the products produced by *gdn* gene cluster.

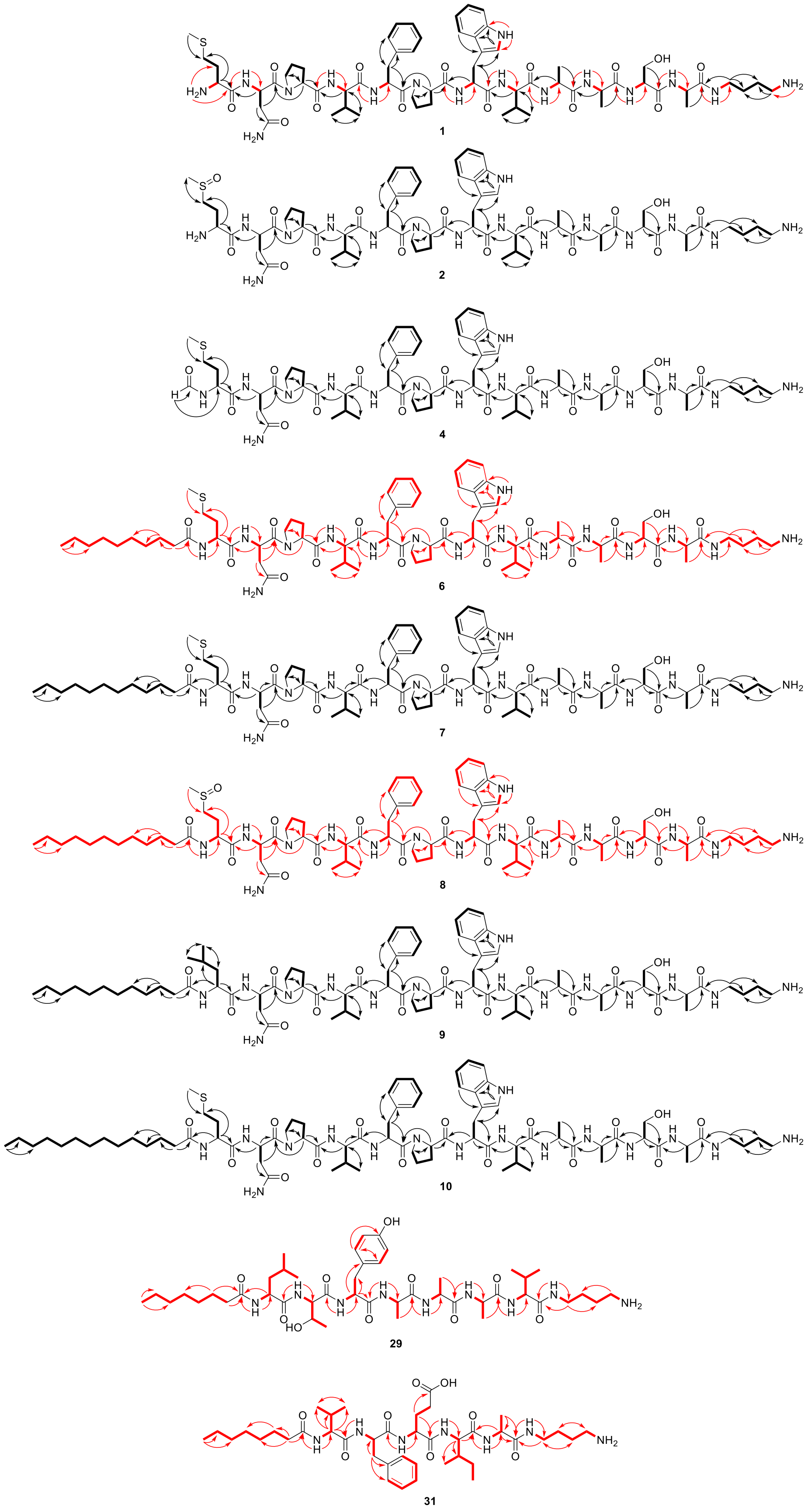

Supplementary Fig. 3. Key COSY and HMBC correlations of **1, 2, 4, 6-10, 29,** and **31** (Black: Signals were recorded in MeOD-d_4, Red: Signals were recorded in DMSO-d_6).

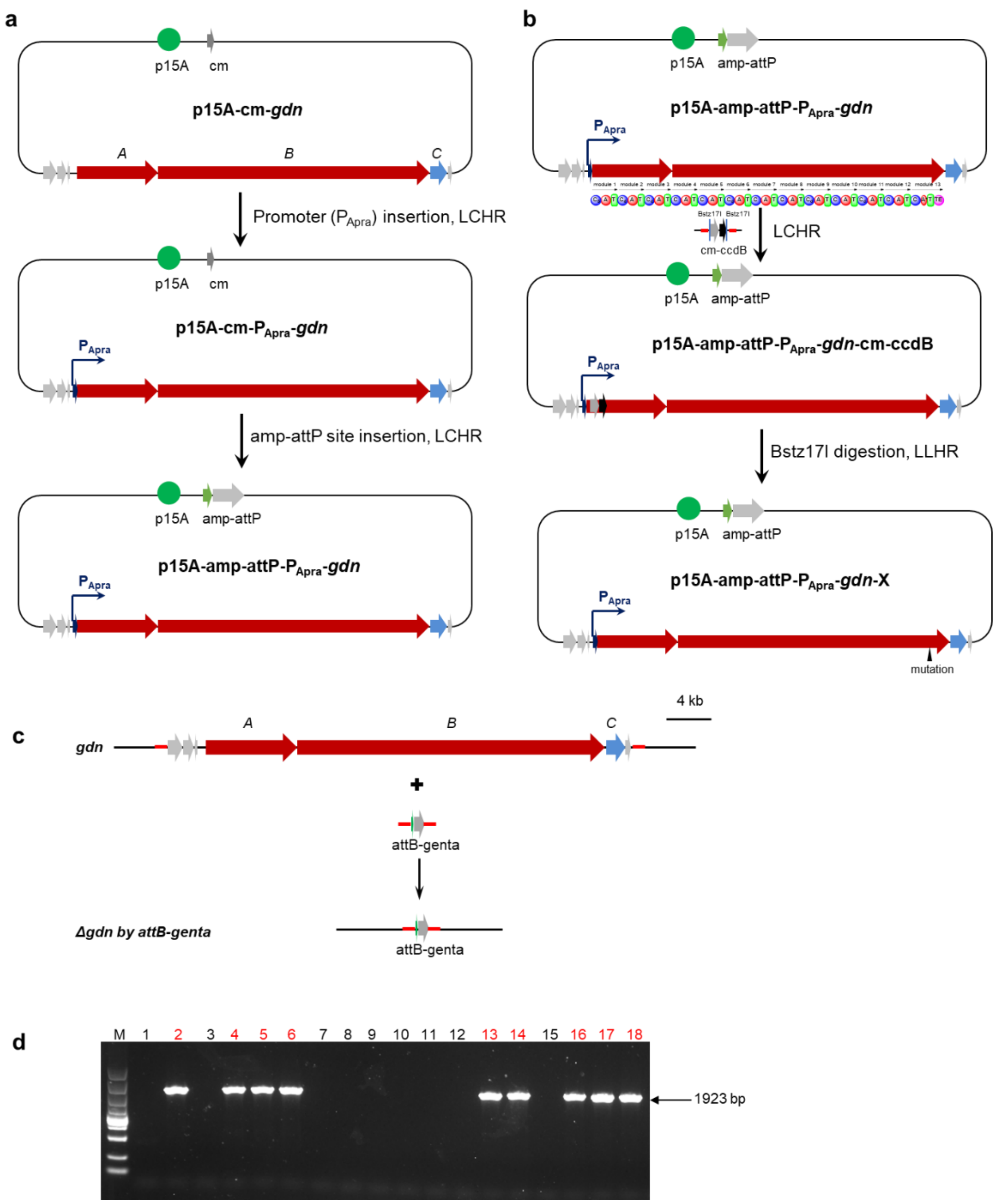

Supplementary Fig. 4. Diagram for the construction of the direct cloning plasmid p15A-cm-*gdn* and modification plasmids, and inactivation of *gdn* BGC. **a.** Diagram for the constructions of the direct cloning plasmid p15A-cm-*gdn* and two modification plasmids p15A-cm-P_{Apra}-*gdn* and p15A-amp-attP-P_{Apra}-*gdn* by LCHR in *E. coli* GB08Red. **b.** Diagram for the constructions of Cs mutants and module 13 mutants of the plasmid p15A-amp-attP-P_{Apra}-*gdn* by RedEx. The correct plasmids were verified by enzyme restriction digestion and DNA sequencing. **c.** Diagram for construction of deletion of the glidonin gene cluster using Redγ-Redαβ7029 recombinases. **d.** PCR verification of deletion of *gdn* by attB-genta. The size of PCR product is 1923 bp for for correct mutant DSM7029*Δgdn*. M: DL 5000 DNA ladder.

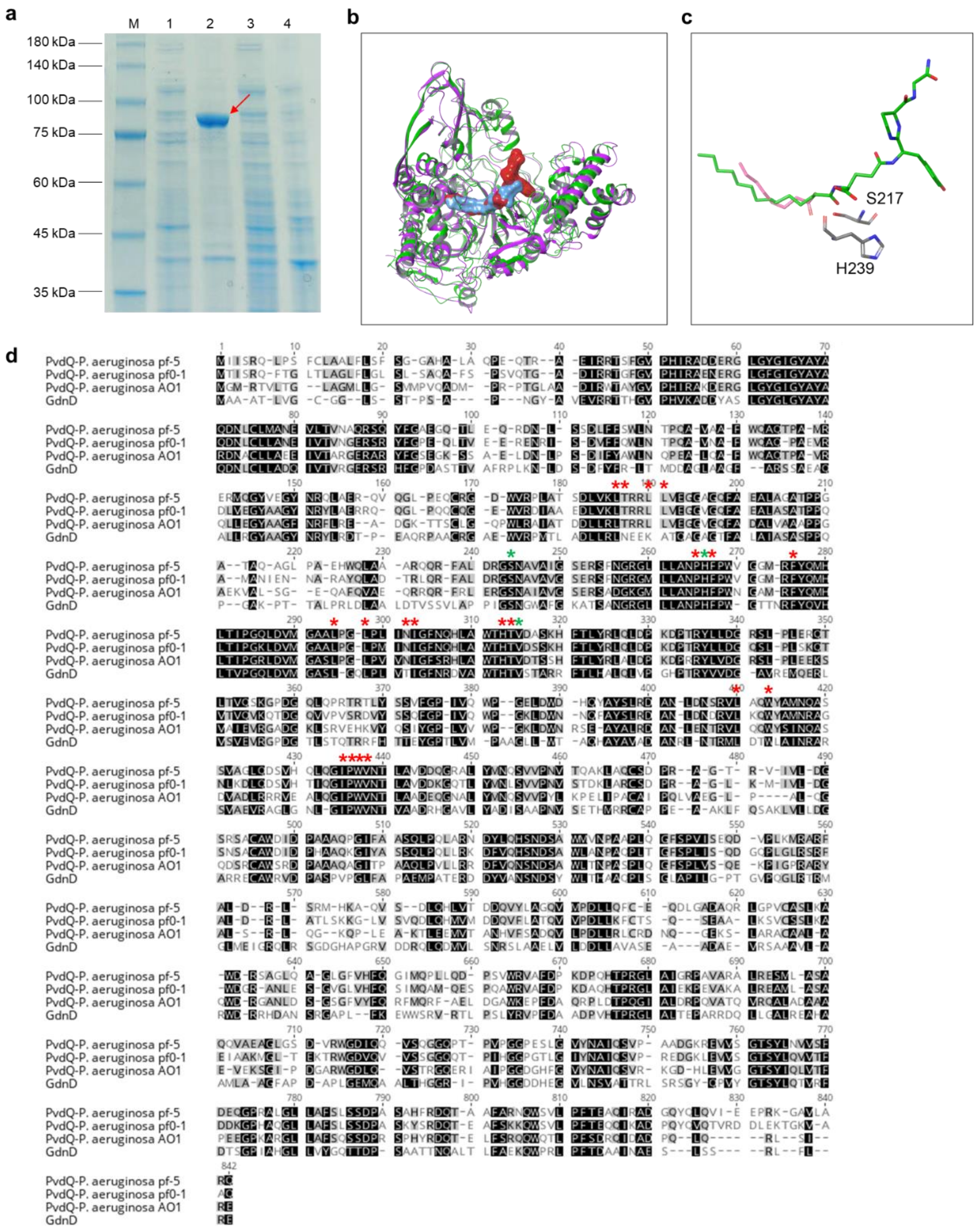

Supplementary Fig. 5. Analysis of acylase GdnD. **a.** SDS-PAGE analysis of whole protein containing GdnD (85 kDa). 1: supernatant of cell debris of *E. coli* BL21/pET28b-GdnD with addition of IPTG, 2: precipitation of cell debris of *E. coli* BL21/pET28b-GdnD with addition of IPTG. 3: supernatant of cell debris of *E. coli* BL21/pET28b-GdnD without addition of IPTG, 4: precipitation of cell debris of *E. coli* BL21/pET28b-GdnD without addition of IPTG. **b.** Structure alignment of GdnD (green) predicted by AlphaFold2 with PvdQ (PDB number: 5UBK, purple), docking substrate C14-D-Met(O) labeled in azure and the substrate of PvdQ labeled in red. **c.** Structure alignment of reported cocrystal structure of PvdQ (PDB number: 5UBK and 3L91), S217 and H239 were the active center of PvdQ. **d.** The sequence similarity between acylases PvdQ and GdnD. PvdQ-P. aeruginosa pf-5: AAY92174.1, PvdQ-P. aeruginosa pf0-1: ABA74304.1, PvdQ-P. aeruginosa AO1: NP_251075.1. The red asterisk indicates the substrate binding residues; the green asterisk indicates the catalytic residues.

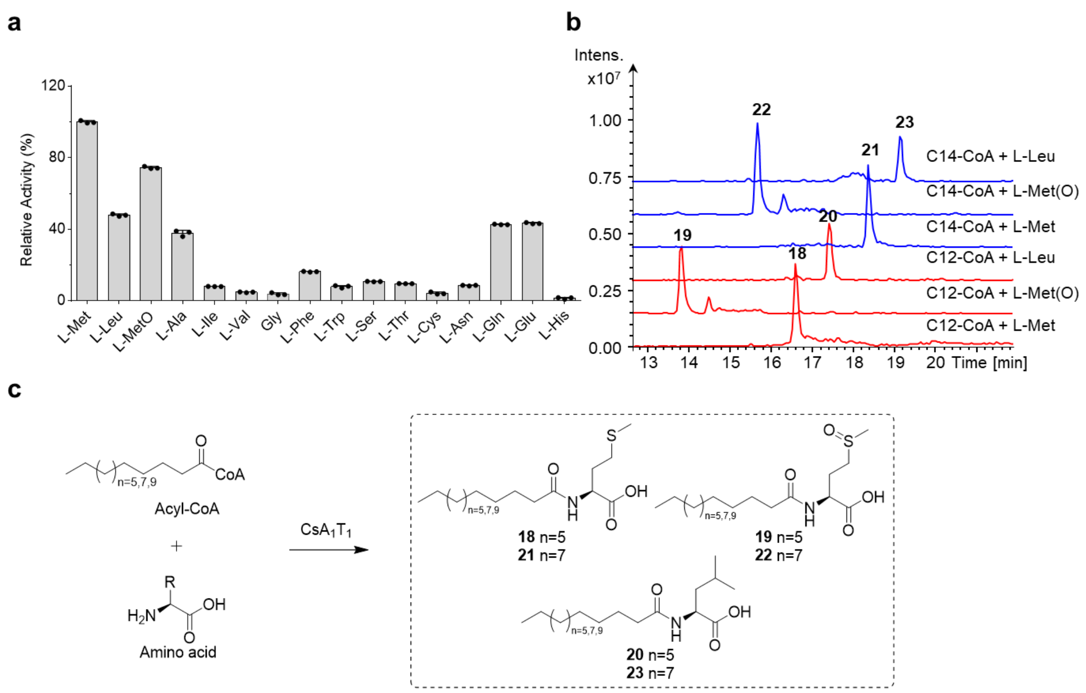

Supplementary Fig. 6. Substrate selectivity of A_1 domain and Cs domain. **a**. The relative activity of A domain of module 1 for the activation of various amino acids. The activity of CsA_1T_1 for activation of L-Met was quantified as a reference (100%). Data are presented as mean values ±SD, n=3 biologically independent samples.. **b**, **c**. *In vitro* assay of CsA_1T_1 domains specificity for donor substrates and acceptor substrates at EICs. Source data are provided as a Source Data file.

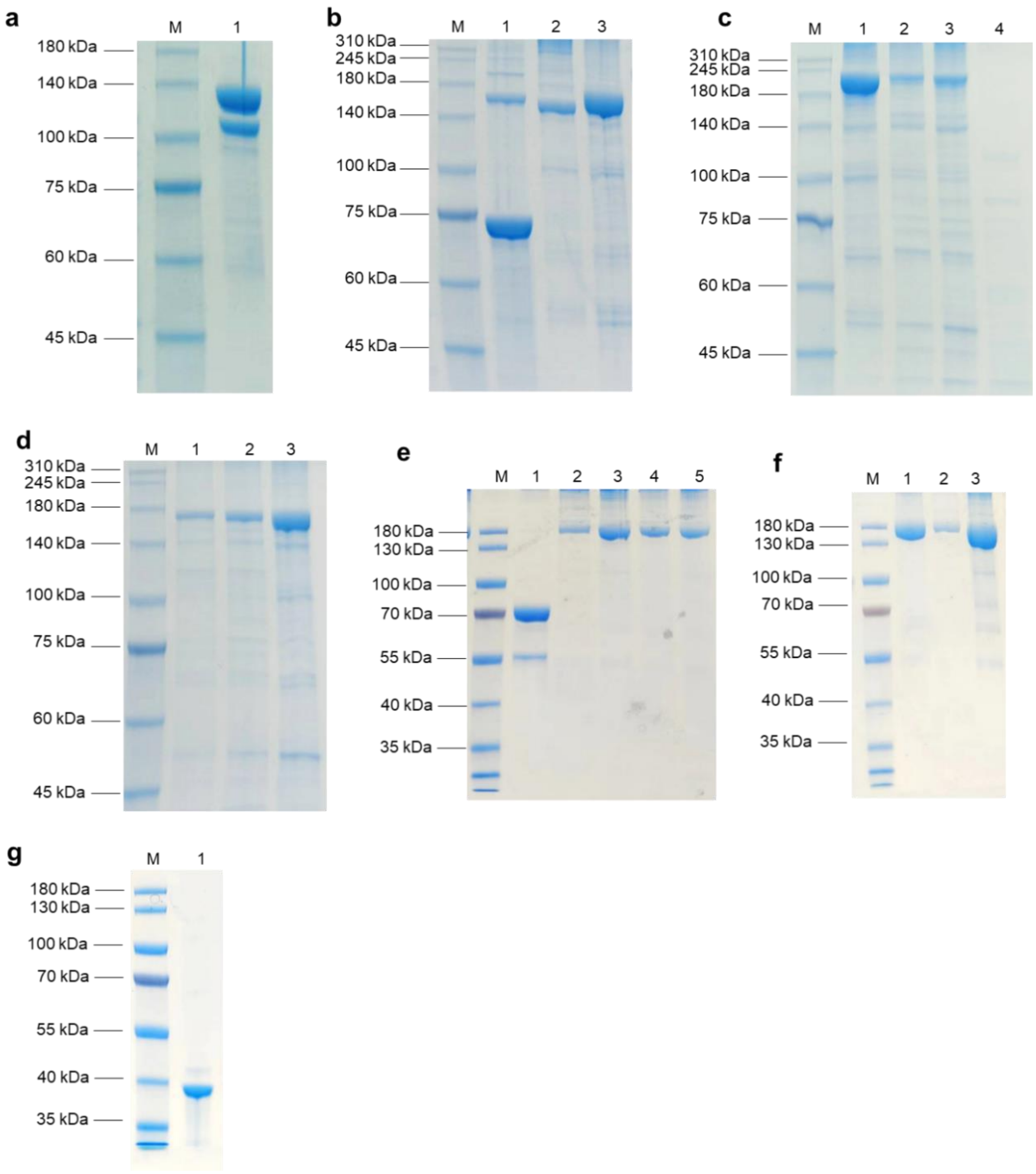

Supplementary Fig. 7. SDS-PAGE analysis of proteins. **a.** SDS-PAGE analysis of purified protein CsA_1T_1 (121 kDa). **b.** SDS-PAGE analysis of module 12 and module 13 proteins. 1: $A_{12}T_{12}$ (70 kDa), 2: $A_{12}T_{12}C_{13}A*$ (140 kDa), and 3: $A_{12}T_{12}C_{13}A*T_{13}$ (153 kDa). **c.** SDS-PAGE analysis of $A_{12}T_{12}C_{13}A^{*}T_{13}TE$ and mutant proteins. 1: $A_{12}T_{12}C_{13}A*T_{13}TE$ (190 kDa), 2: $A_{12}T_{12}C_{13}A*T_{13}TE_{Mut1}$(S10391A), 3: $A_{12}T_{12}C_{13}A*T_{13}TE_{Mut2}$(S10624A), 4: $A_{12}T_{12}C_{13}A*T_{13}TE_{Mut1/2}$(S10391A/S10624A). **d.** SDS-PAGE analysis of three mutants of $A_{12}T_{12}C_{13}A*T_{13}$. 1: $A_{12}T_{12}C_{13}A*T_{13}$(D9965A), 2: $A_{12}T_{12}C_{13}A*T_{13}$(S9963A), 3: $A_{12}T_{12}C_{13}A*T_{13}$(E9984A). **e.** SDS-PAGE analysis of mutants of $A_{12}T_{12}C_{13}A*T_{13}$ and A_1T_1. 1: $A_{12}T_{12}$, 2: $A_{12}T_{12}C_{13}A*T_{13}$(S9963Q), 3: $A_{12}T_{12}C_{13}A*T_{13}$(S9963V), 4: $A_{12}T_{12}C_{13}A*T_{13}$(E9984M), 5: $A_{12}T_{12}C_{13}A*T_{13}$(E9984D). **f.** SDS-PAGE analysis of mutants of $A_{12}T_{12}C_{13}A*T_{13}$. 1: $A_{12}T_{12}C_{13}A*T_{13}$(D9965L), 2: $A_{12}T_{12}C_{13}A*T_{13}$(D9965E), 3: $A_{12}T_{12}C_{13}A^{*}T_{13}$(H9721A). **g.** SDS-PAGE analysis of purified protein Fmt (35 kDa).

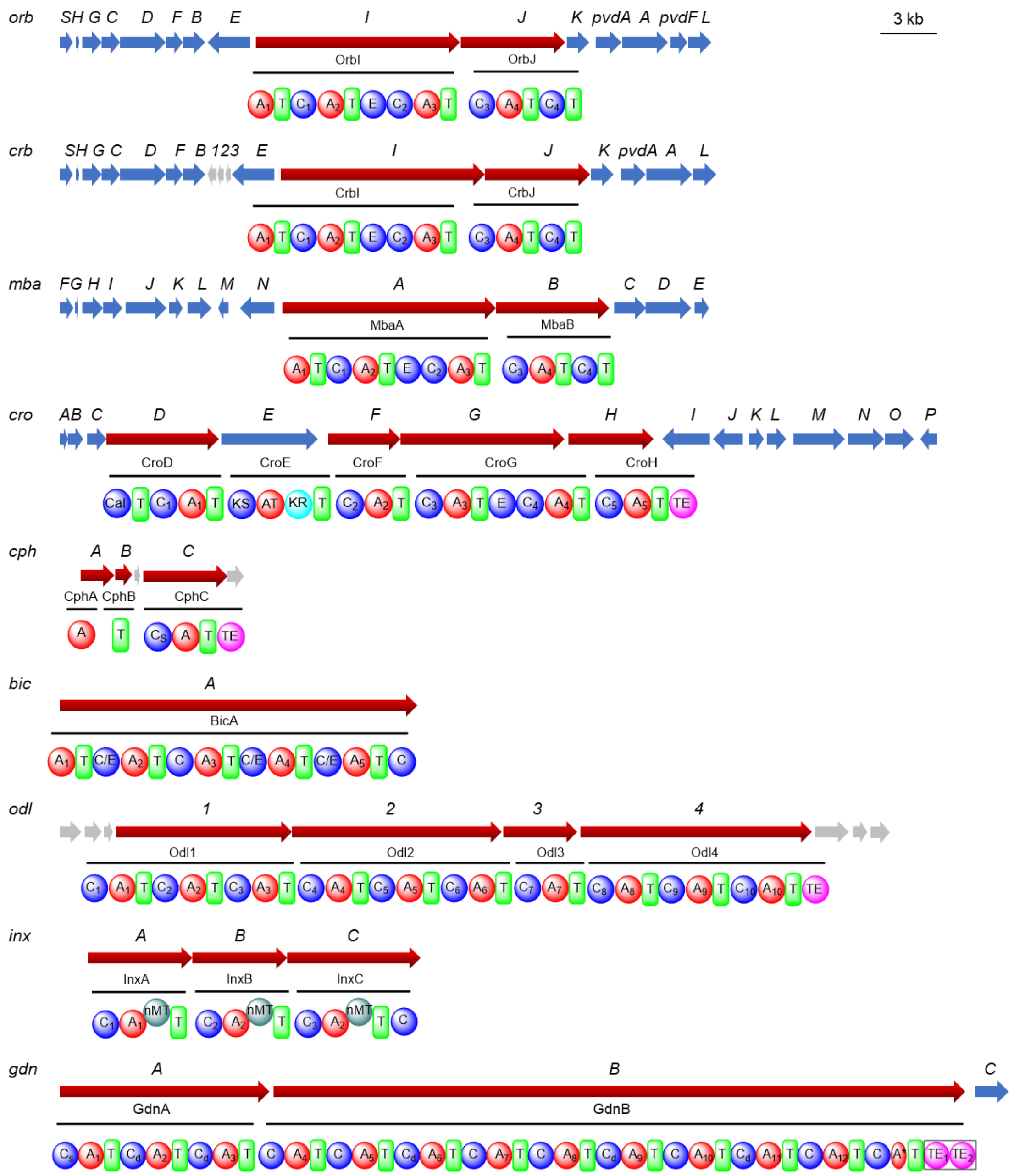

Supplementary Fig. 8. Gene organization of eight nonribosomal peptide synthetase (NRPS) gene clusters. orb: ornibactin, crb: caribactin, mba: malleobactin, cro: crochelin, cph: cepaciachelin, bic: bicornutin A1, odl: odilorhabdin (NOSO-95), inx: Rhabdopeptide/xenortide-like peptides (RXPs), gdn: glidonin. Red indicates NRPS core genes, blue indicates additional genes, grey indicates non-related genes. C: condensation domain, A: adenylation domain, T: thiolation domain, E: epimerization domain, TE: thioesterase domain, MT: methyltransferase domain, Cal: acyl-CoA ligase domain, KR: ketoreductase domain, KS: ketoacylsynthase domain, AT: acyltransferase domain.

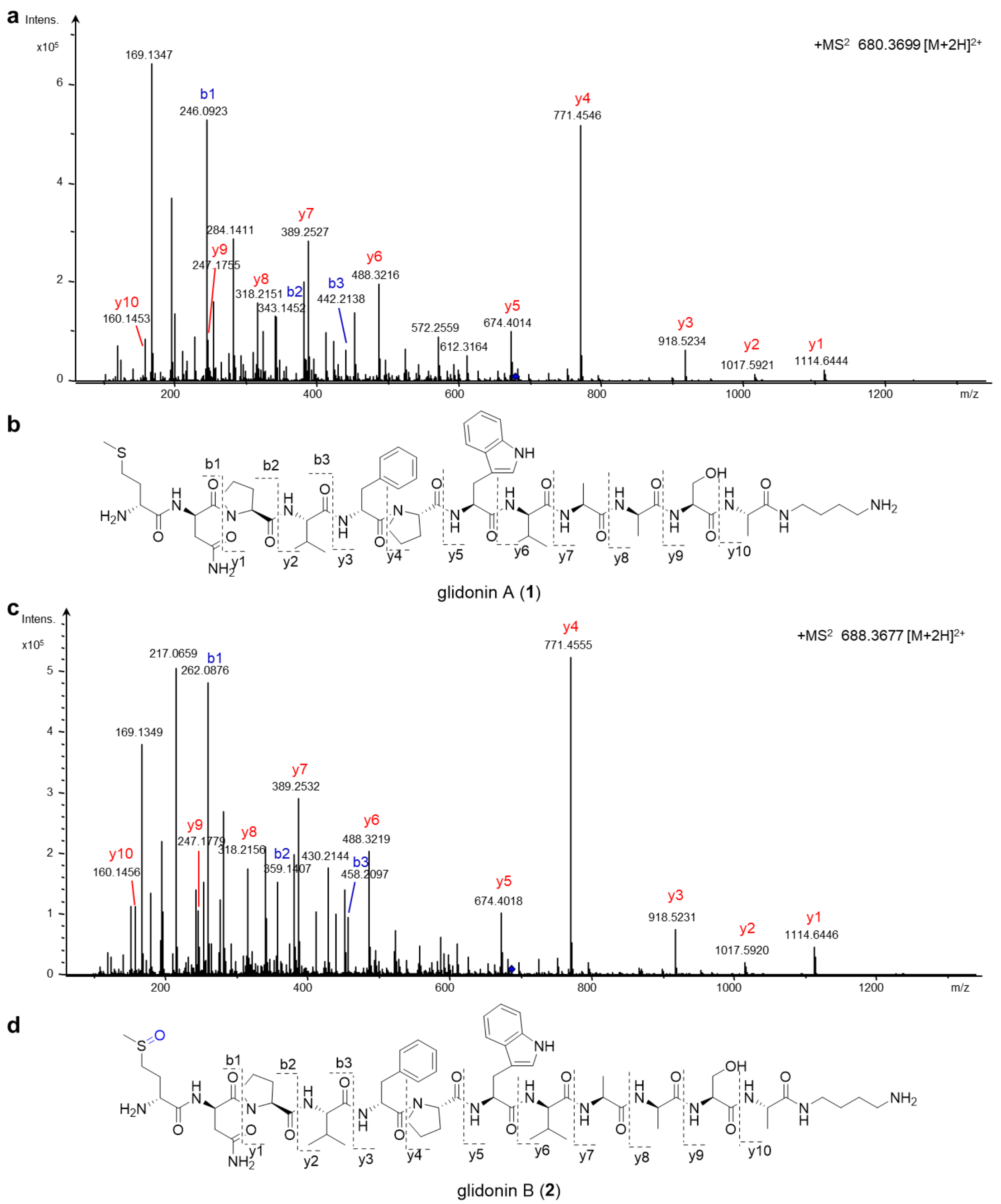

Supplementary Fig. 9. HR-ESI-MS spectra and MS/MS fragmentation of **1** and **2**. **a.** MS/MS fragmentation of **1**; **b.** Structure of **1**; **c.** MS/MS fragmentation of **2**; **d.** Structure of **2**.

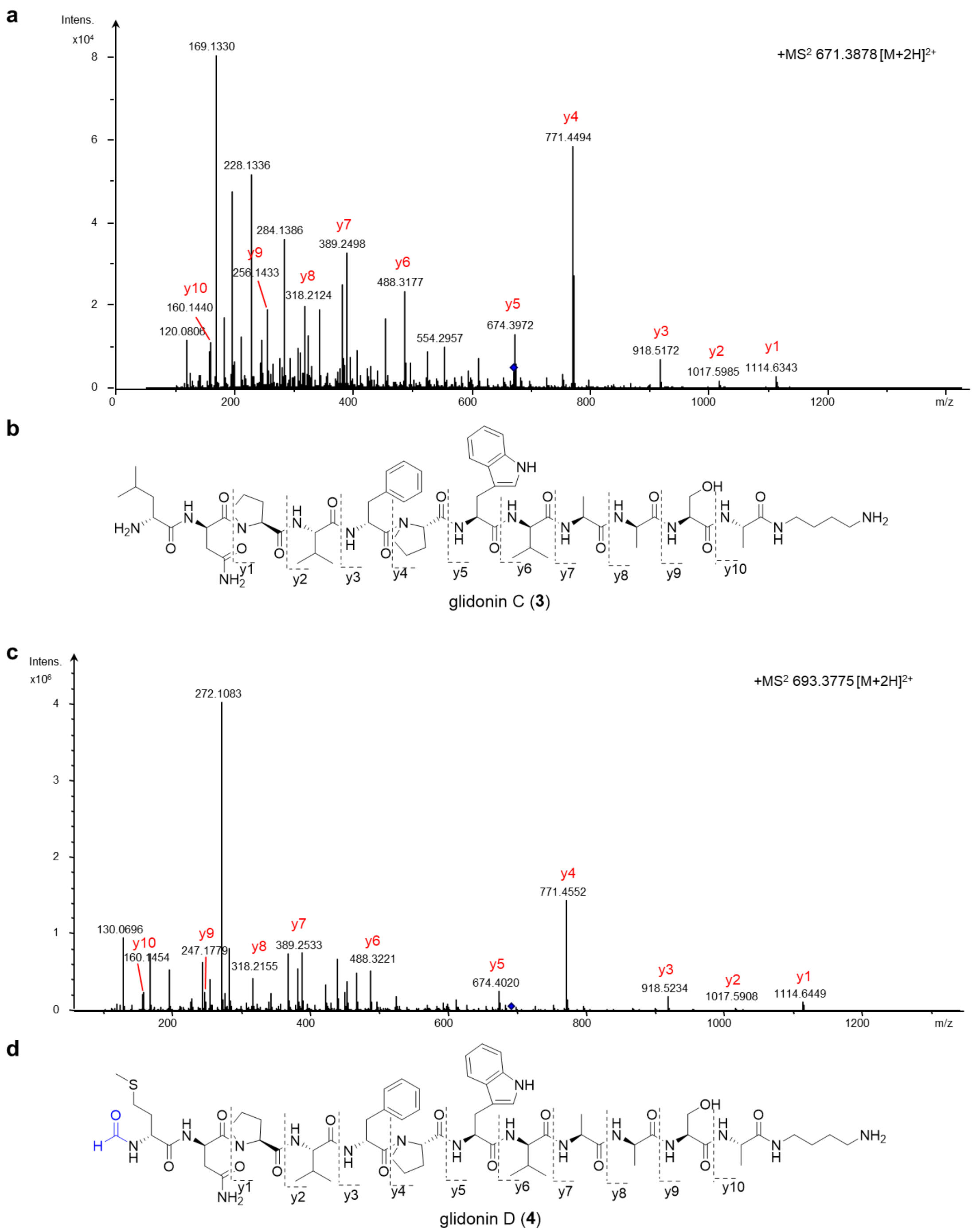

Supplementary Fig. 10. HR-ESI-MS spectra and MS/MS fragmentation of **3** and **4**. **a.** MS/MS fragmentation of **3**; **b.** Structure of **3**; **c.** MS/MS fragmentation of **4**; **d.** Structure of **4**.

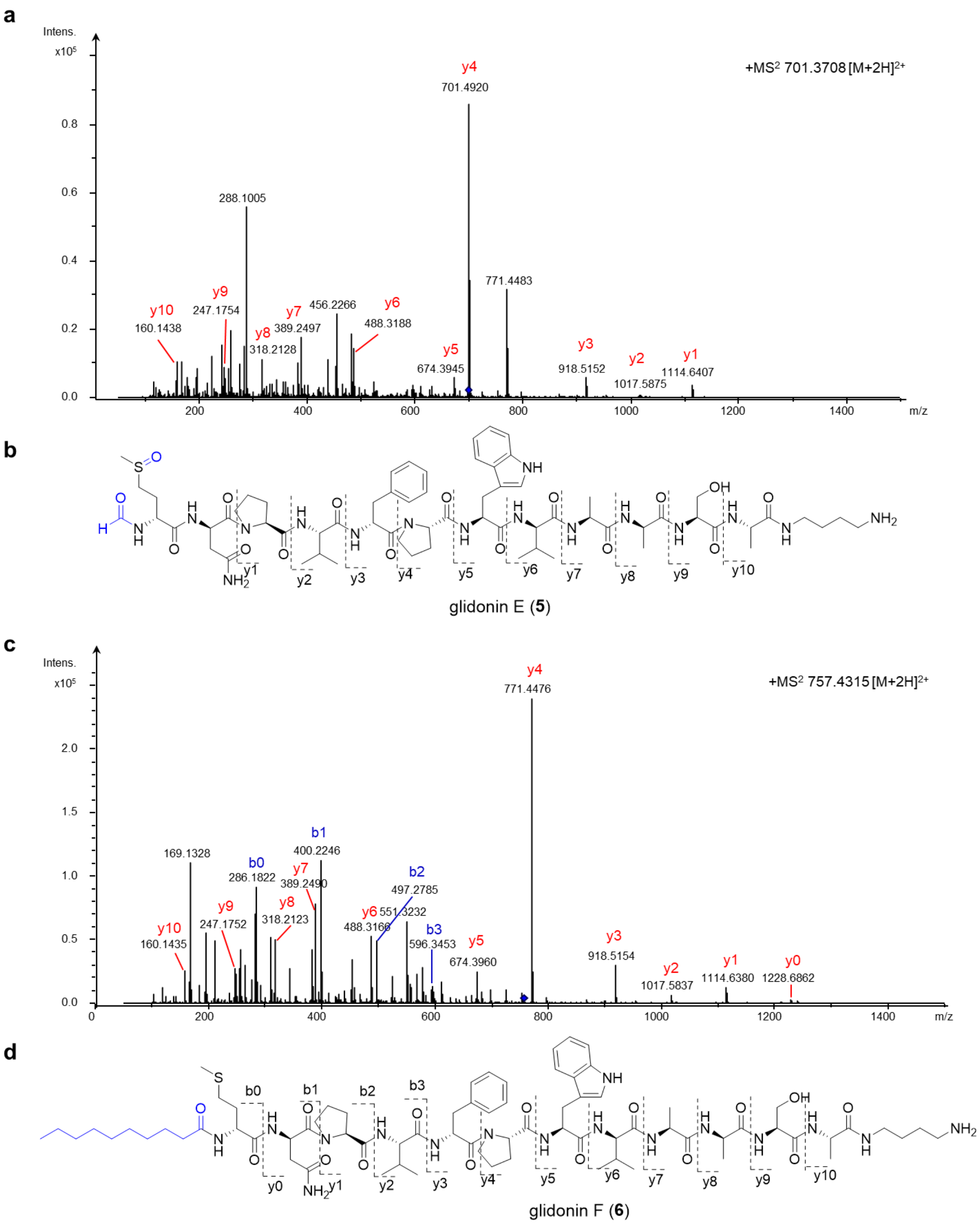

Supplementary Fig. 11. HR-ESI-MS spectra and MS/MS fragmentation of **5** and **6**. **a.** MS/MS fragmentation of **5**; **b.** Structure of **5**; **c.** MS/MS fragmentation of **6**; **d.** Structure of **6**.

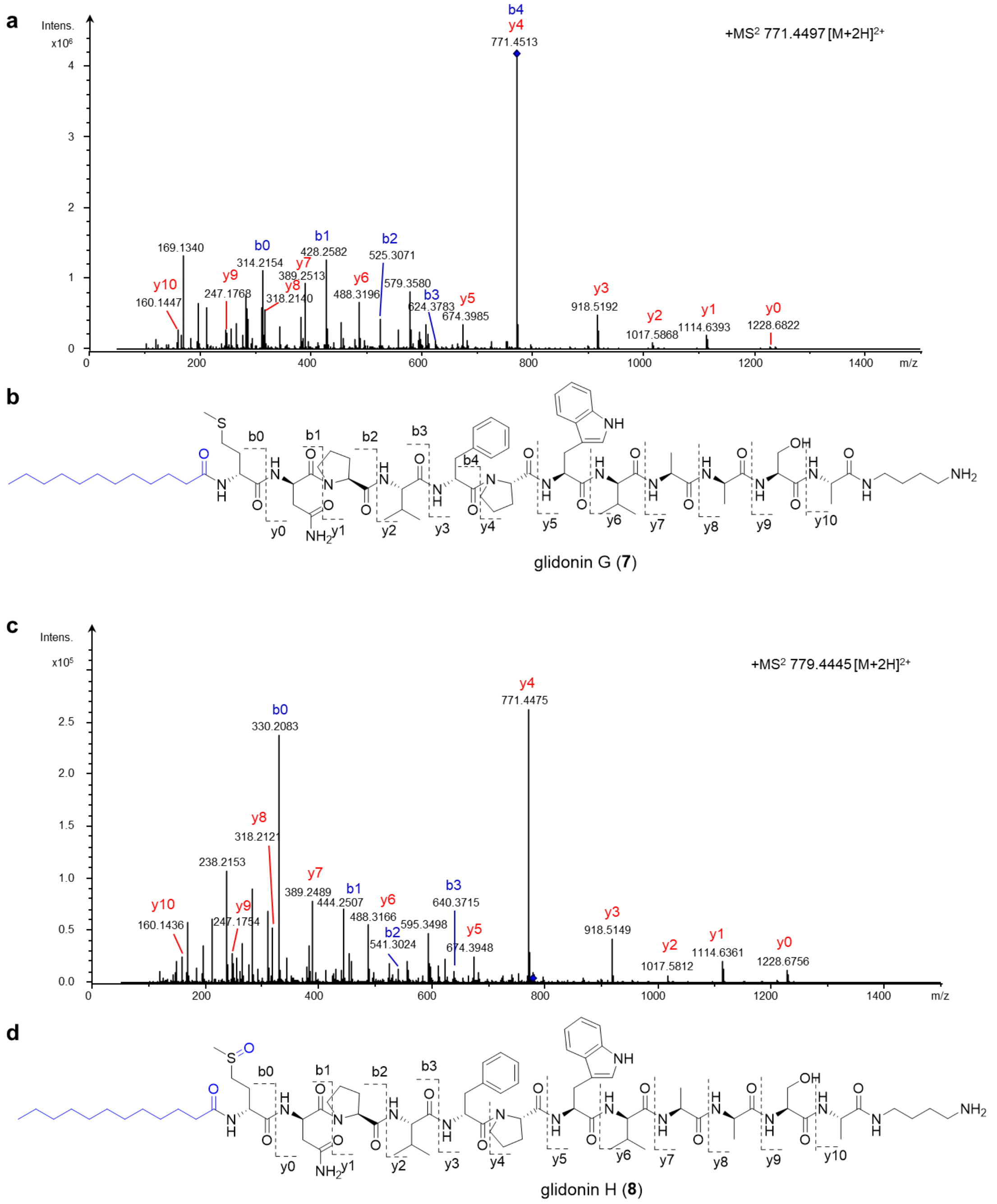

Supplementary Fig. 12. HR-ESI-MS spectra and MS/MS fragmentation of **7** and **8**. **a.** MS/MS fragmentation of **7**; **b.** Structure of **7**; **c.** MS/MS fragmentation of **8**; **d.** Structure of **8**.

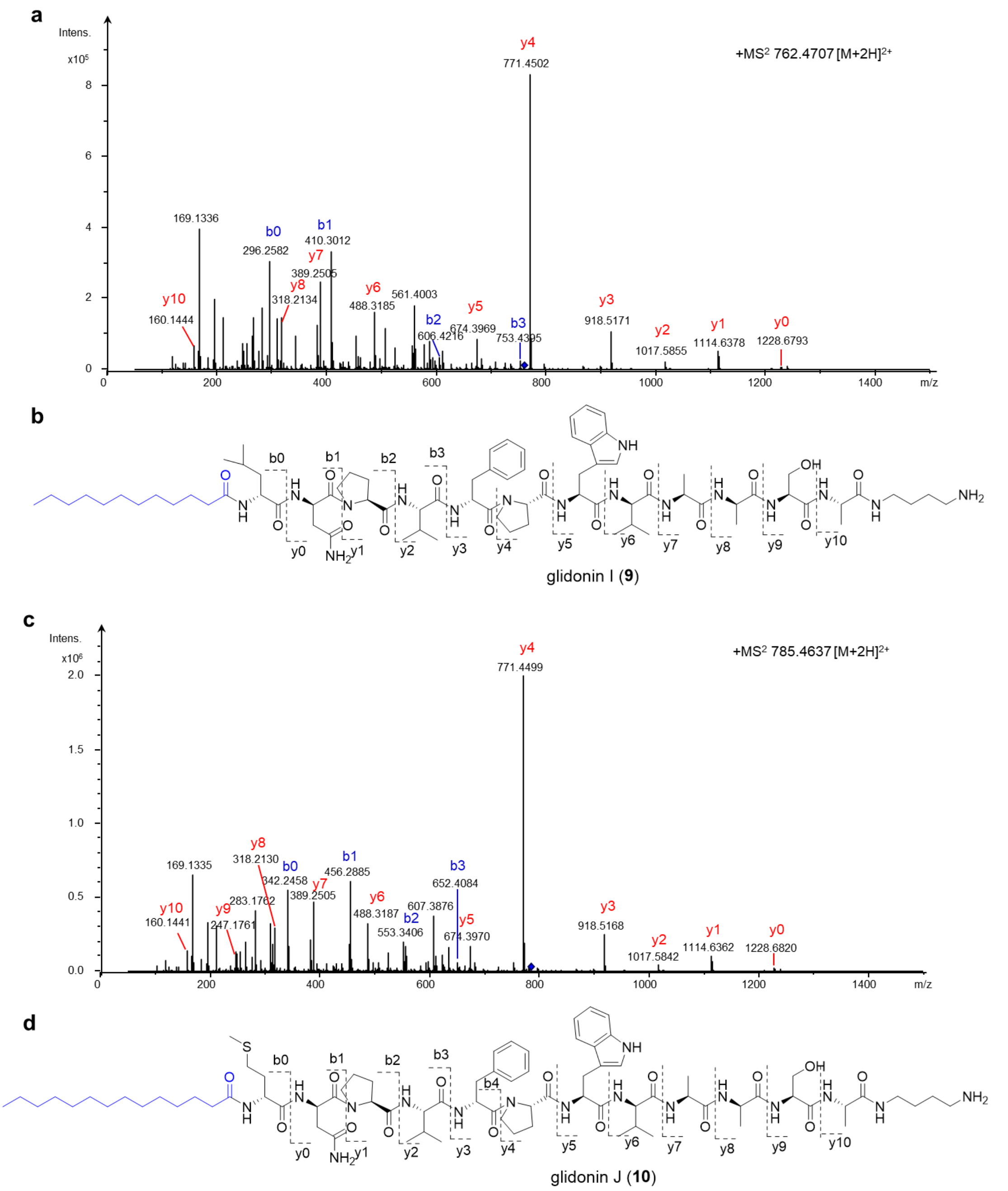

Supplementary Fig. 13. HR-ESI-MS spectra and MS/MS fragmentation of **9** and **10**. **a.** MS/MS fragmentation of **9**; **b.** Structure of **9**; **c.** MS/MS fragmentation of **10**; **d.** Structure of **10**.

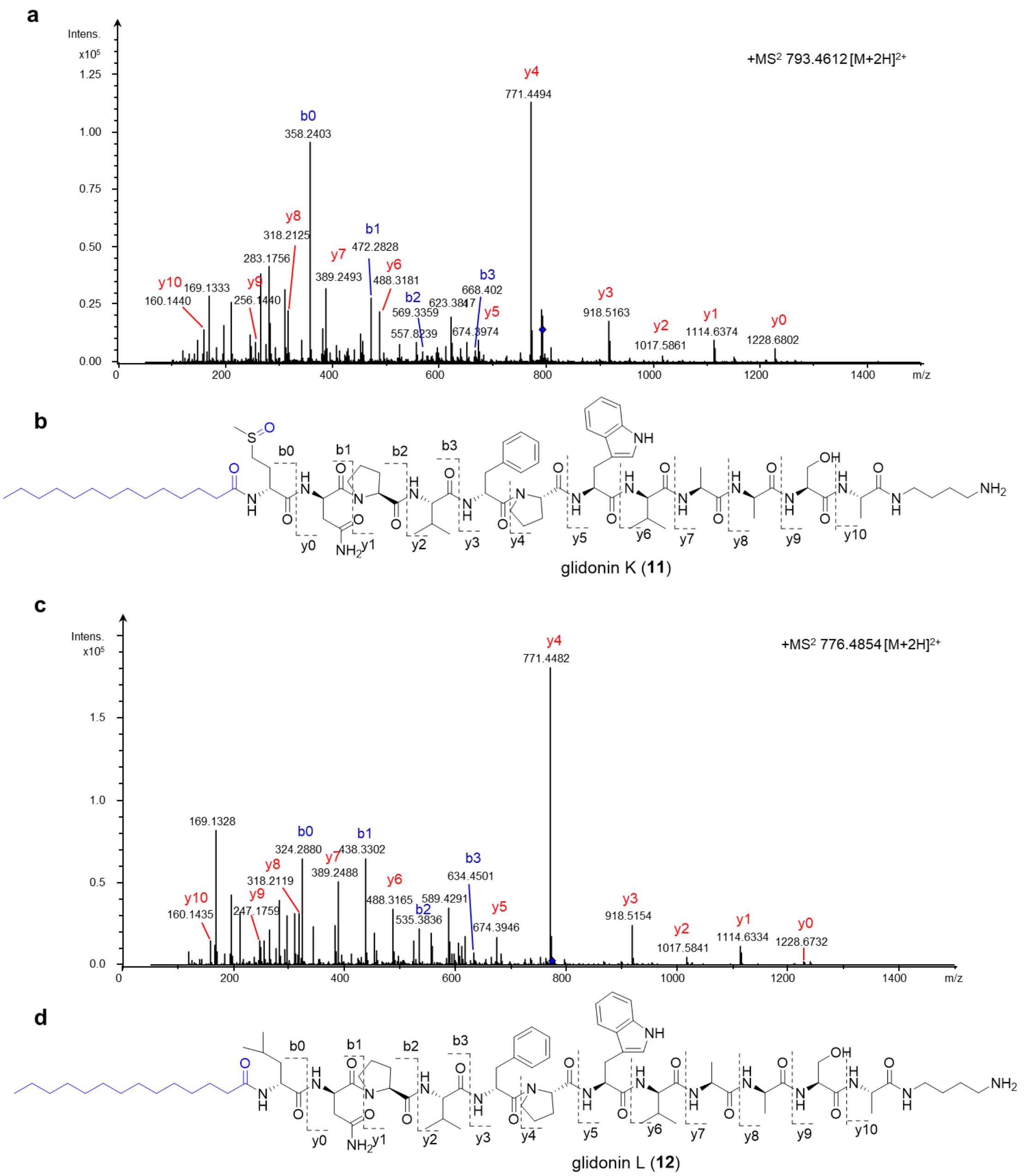

Supplementary Fig. 14. HR-ESI-MS spectra and MS/MS fragmentation of **11** and **12**. **a.** MS/MS fragmentation of **11**; **b.** Structure of **11**. **c.** MS/MS fragmentation of **12**; **d.** Structure of **12**.

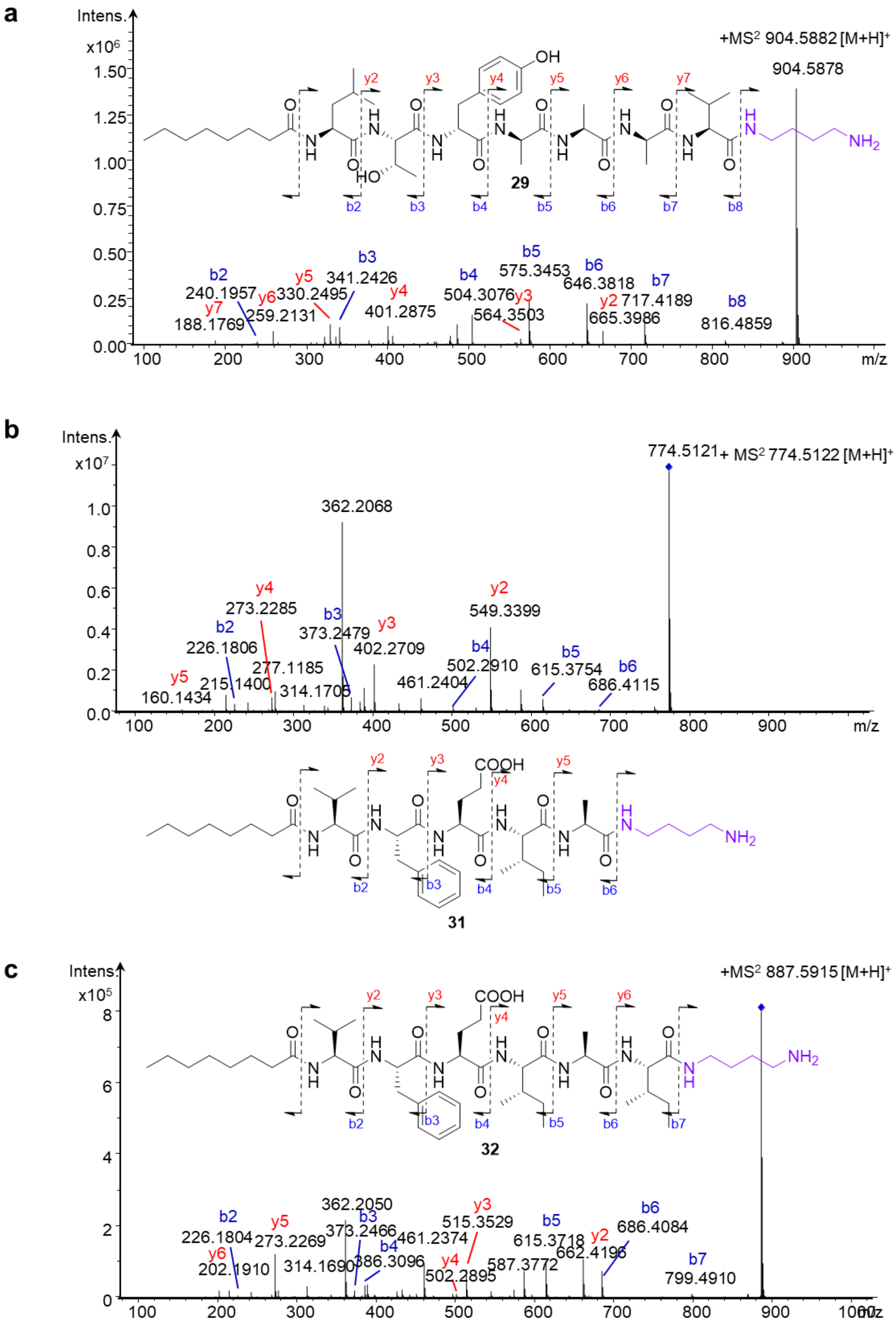

Supplementary Fig. 15. HR-ESI-MS spectra and MS/MS fragmentation of **29**, **31** and **32**.

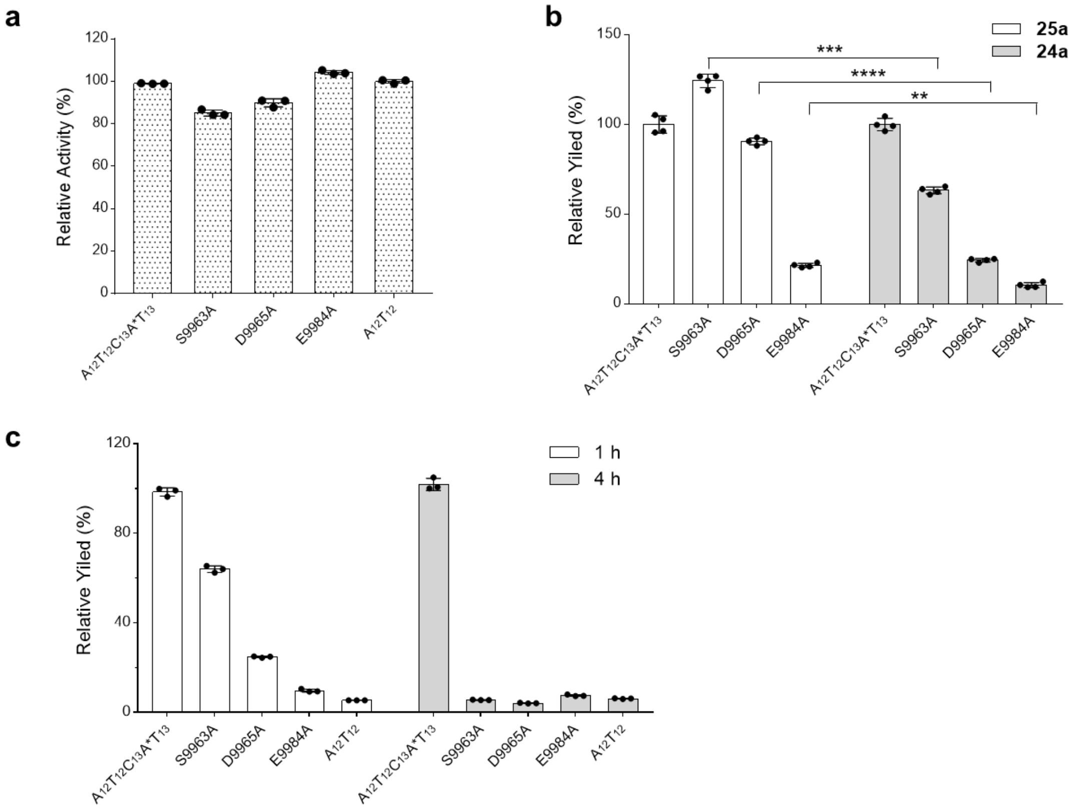

Supplementary Fig. 16. Function analysis of $A_{12}T_{12}C_{13}A*T_{13}$ and its mutants. **a.** The relative activity of A_{12} domain from proteins $A_{12}T_{12}C_{13}A*T_{13}$, its three mutants and $A_{12}T_{12}$ for the substrate L-Ala. The activity of $A_{12}T_{12}C_{13}A*T_{13}$ for activation of L-Ala was quantified as a reference (100%). Data are presented as mean values ±SD, n=3 biologically independent samples.. **b.** Yield comparison of the target products **24a** and **25a** obtained from $A_{12}T_{12}C_{13}A*T_{13}$ and its three mutants for testing the condensation activity to substrates putrescine and 1,5-diaminopentane. The yield of **24a** and **25a** generated from the wild-type $A_{12}T_{12}C_{13}A*T_{13}$ was quantified as a reference (100%), respectively. Data are presented as mean values ±SD, n=4 biologically independent samples. *P* values were determined by two-tailed unpaired *t* test. $^{**}p<0.01$, $^{***}p<0.001$, $^{****}p<0.0001$. **c.** Yield comparison of the target product **24a** in two timepoint (1 h and 4 h), which were obtained from $A_{12}T_{12}C_{13}A*T_{13}$ and its three mutants. The yield of **24a** generated from the $A_{12}T_{12}C_{13}A*T_{13}$ was quantified as a reference (100%), respectively. Data are presented as mean values ±SD, n=4 biologically independent samples. Source data are provided as a Source Data file.

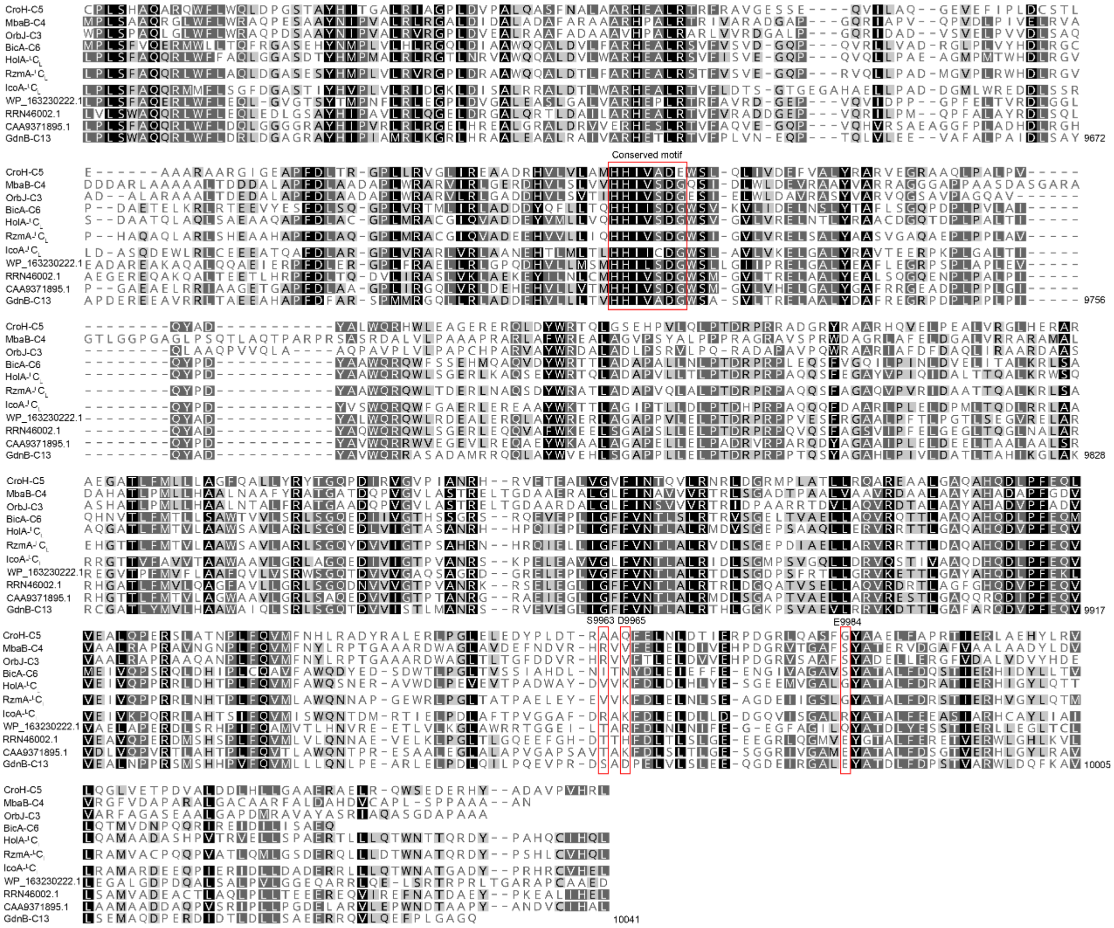

Supplementary Fig. 17. Sequence alignment of different condensation domains. CroH-C5 (AJE23274.1), MbaB-C4 (WP_011402368.1), OrbJ-C3 (AUD11993.1), BicA-C6 (AFP87549.1), HolA-$^{L}C_{L}$ (CBW76913.1), RzmA-$^{L}C_{L}$ (CBW76463.1), IcoA-$^{L}C_{L}$ (WP_080752264.1), and three condensation domains gained from the BlastP of GdnB-C_{13}-A*, including condensation domains from *Caulobacter rhizosphaerae* (WP_163230222.1), *Lautropia dentalis* (RRN46002.1) and uncultured *Gemmatimonadetes bacterium* (CAA9371895.1) . S9963, D9965 and E9984 were marked with red rectangle, HHXXDG is the conserved motif.

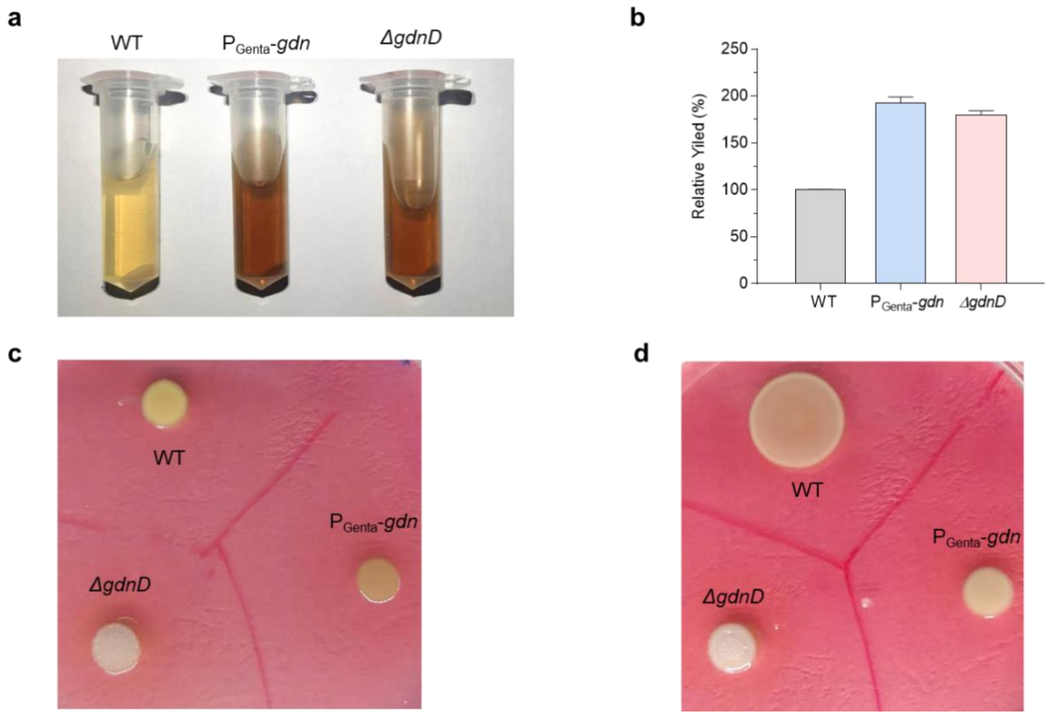

Supplementary Fig. 18. The comparsion of the characteristics between wild type (WT) and mutants. **a, b.** The comparsion of the supernatant of the fermentation cultures. The OD_{600} of the supernatant of the fermentation culture of the wild type was quantified as a reference (100%). Data are presented as mean values ±SD, n=3 biologically independent samples. Source data are provided as a Source Data file. **c.** The comparsion of colony morphology on the CYMG plate (1.2% agar). **d.** The comparsion of colony morphology on the CYMG plate (0.5% agar).

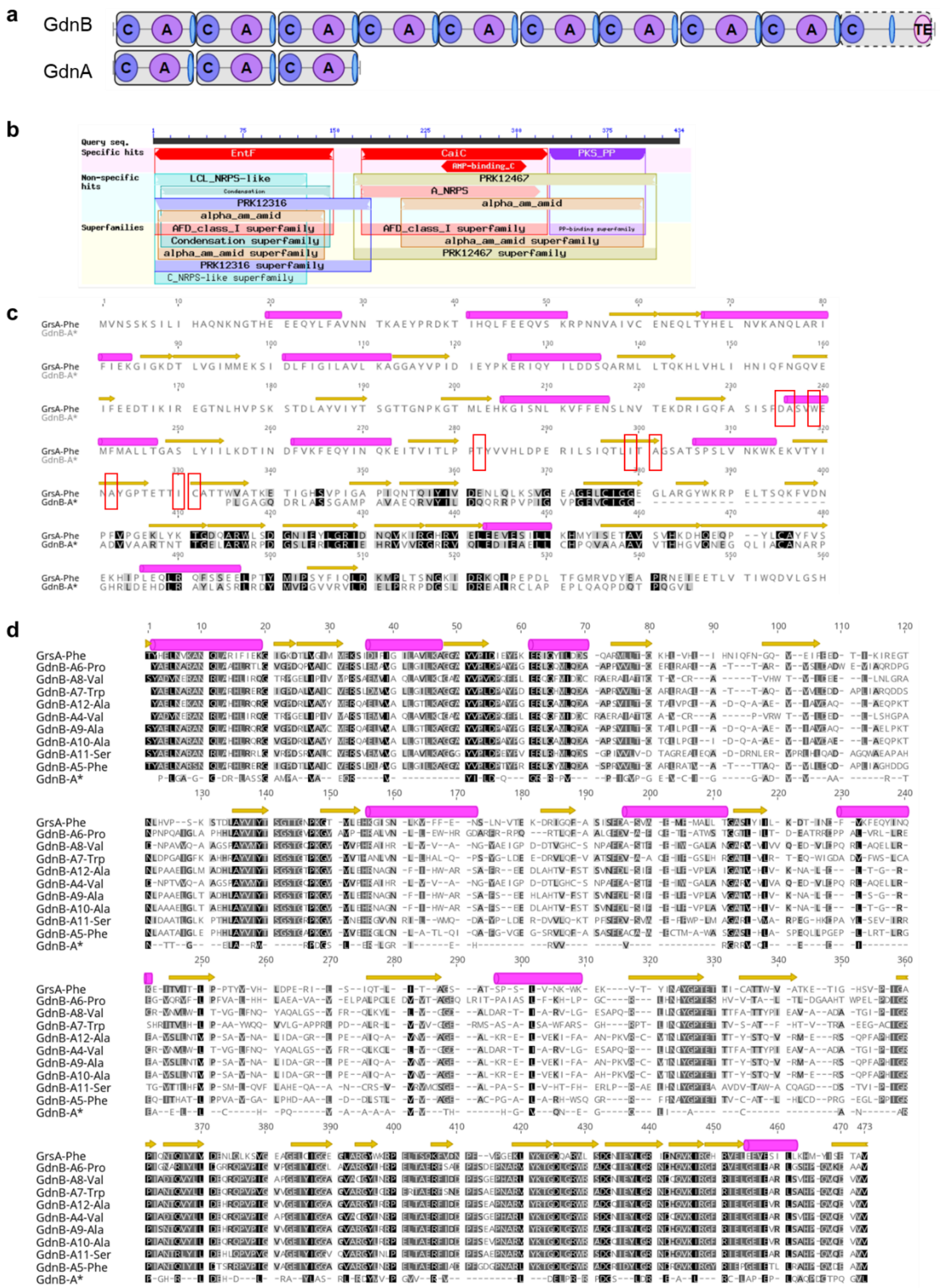

Supplementary Fig. 19. Bioinformatic analysis of Gdn NRPS gene cluster. **a.** AntiSMASH prediction of Gdn NRPS gene cluster. **b.** BlastP analysis of the $C_{13}A^{*}T_{13}$ domains of the GdnB. **c.** Sequence alignment of GdnB-A* and GrsA(1AMU_A). The red frame indicates Stachelhaus codes. **d.** Sequence alignment of GdnB-A* and A domains of GdnB (WP_053013656.1) of the producer *Caldimonas brevitalea* (previously identified as *Schlegelella brevitalea* DSM 7029) based on blastP analysis. Alpha helix in magenta and β-strands in yellow.

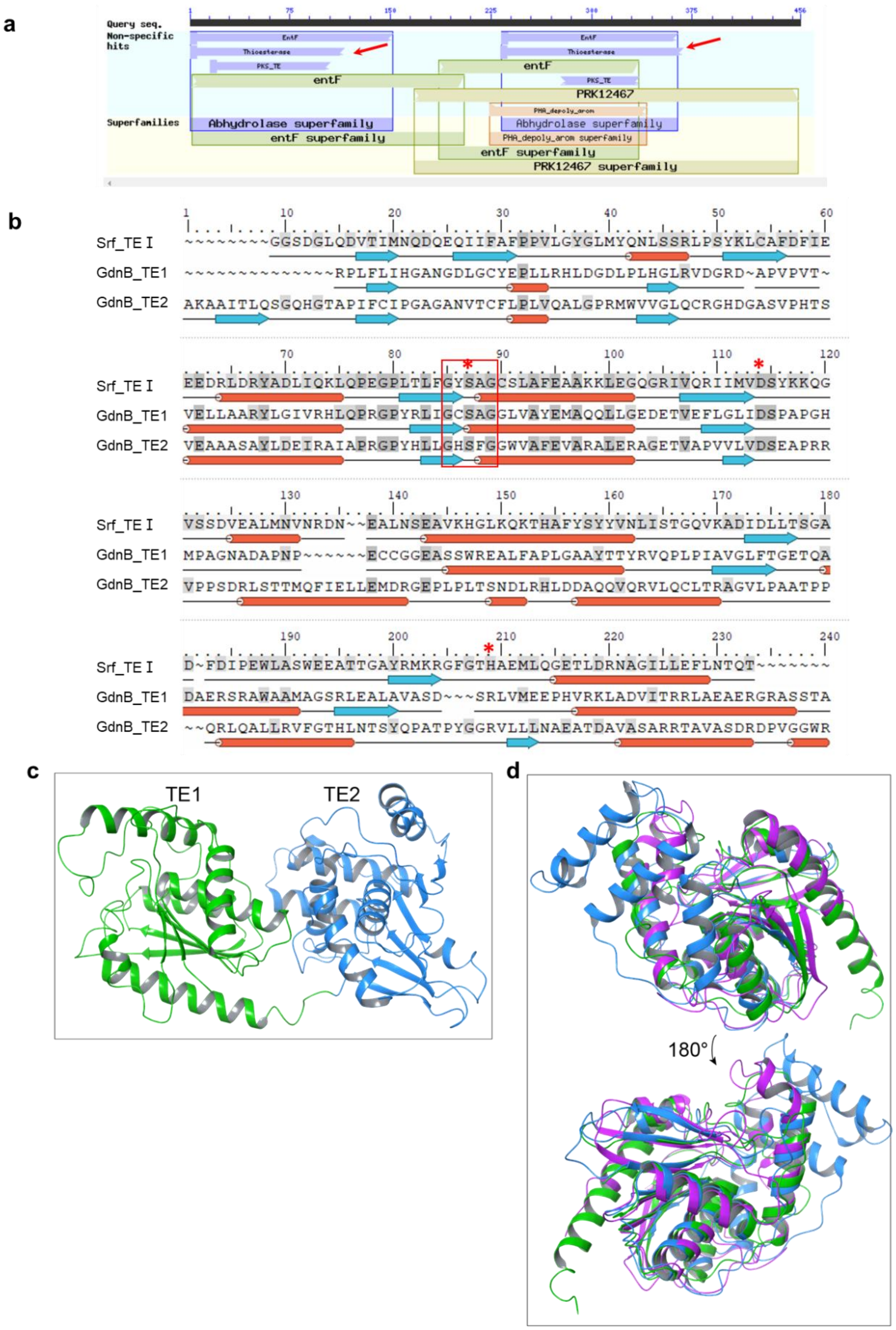

Supplementary Fig. 20. Bioinformatic analysis of GdnB-TE domain and structural prediction. **a.** BlastP analysis of the GdnB-TE domain. The red arrow indicates two TE domains. **b.** Sequence alignment of GdnB-TE$_1$, GdnB-TE$_2$ and Srf_TE Ⅰ (surfactin). The red frame indicates conserved site residues GXSXG. The red asterisk indicates three catalytic residues Ser, Asp, and His, helices in red, β-strands in blue. **c.** Overall structure of GdnB-TE domain predicted by AlphaFold2, which contains two TE domains. **d.** Structure alignment of TE$_1$ (green), TE$_2$ (azure) and the SrfTE Ⅰ (PDB number: 1JMK) labeled in violet.

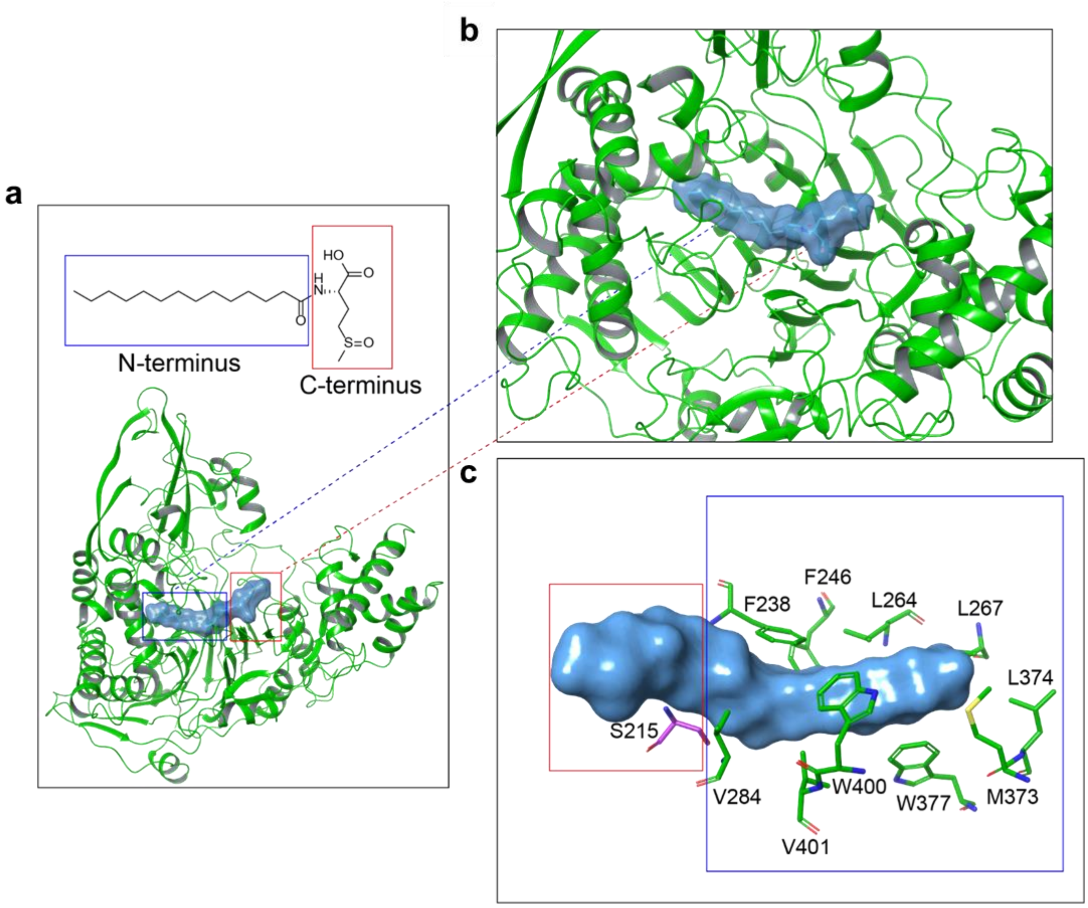

Supplementary Fig. 21. Analysis of GdnD docking with substrate C14-D-Met(O). **a.** GdnD docking with C14-D-Met(O), rectangles in bule and red represent the N-terminus and C-terminus of substrate. **b.** Zoom view of the binding pocket of GdnD. **c.** The hydrophobic pocket in N-terminus, S215 was the active site of GdnD which located in C-terminus.

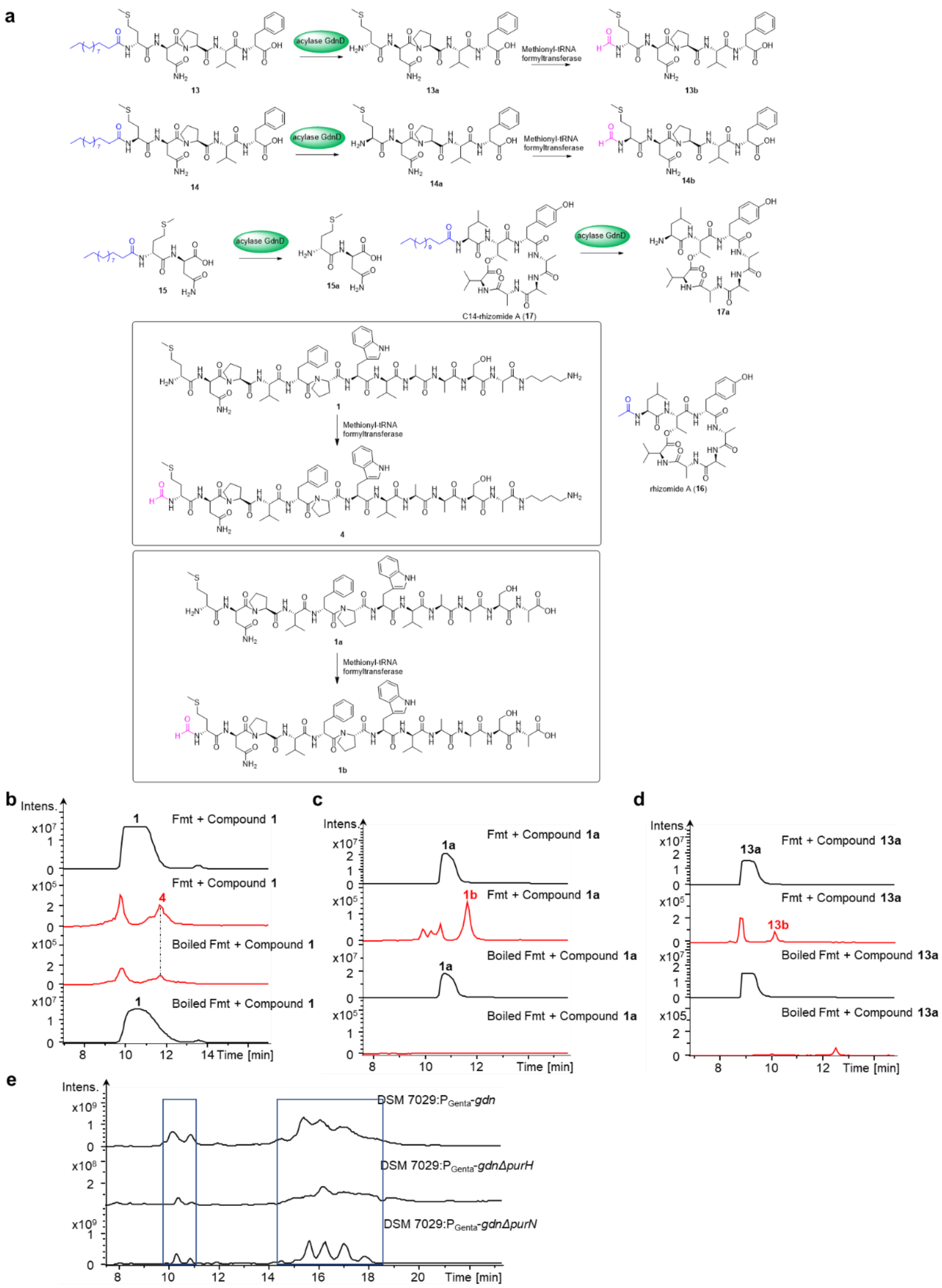

Supplementary Fig. 22. The structures of lipopeptides and NRPs, and *in vitro* assay of Methionyl-tRNA formyltransferase (Fmt). **a.** The structures of synthesized lipopeptides, and corresponding nonacylated compounds and formylated compounds. **b.-d.** HPLC-MS analysis of reaction mixture of protein Fmt with different substrates **1**, **1a** and **13a**. **4** was formyl product of **1**, **1b** was formyl product of **1a**, **13b** was formyl product of **13a**. **e.** LC-MS analysis of crude extracts of the activated mutant DSM 7029:P_{Genta}-*gdn* and two deleted mutants of formyltransferases PurH and PurN.

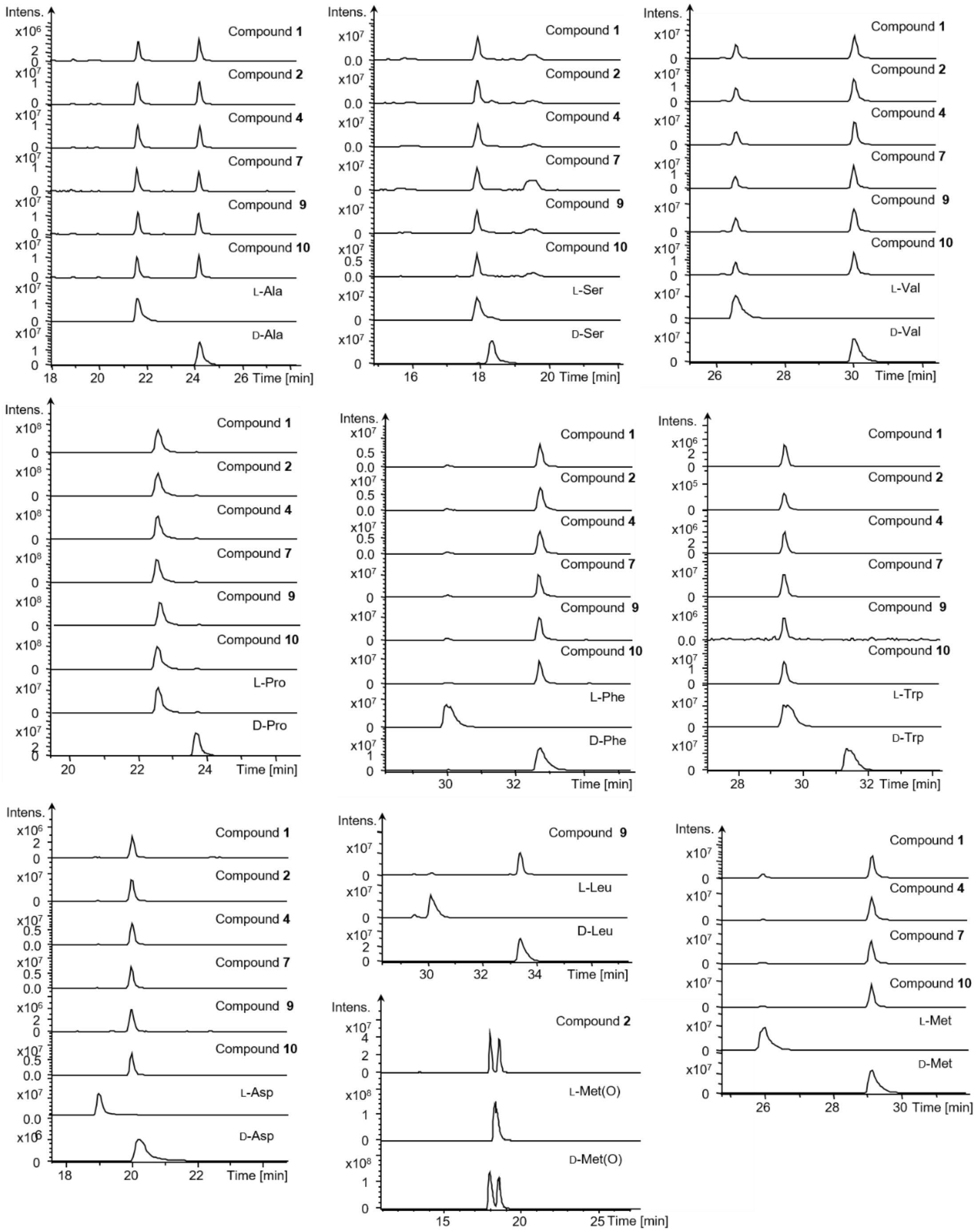

Supplementary Fig. 23. Marfey’s analysis of the amino acid constituents of glidonins.

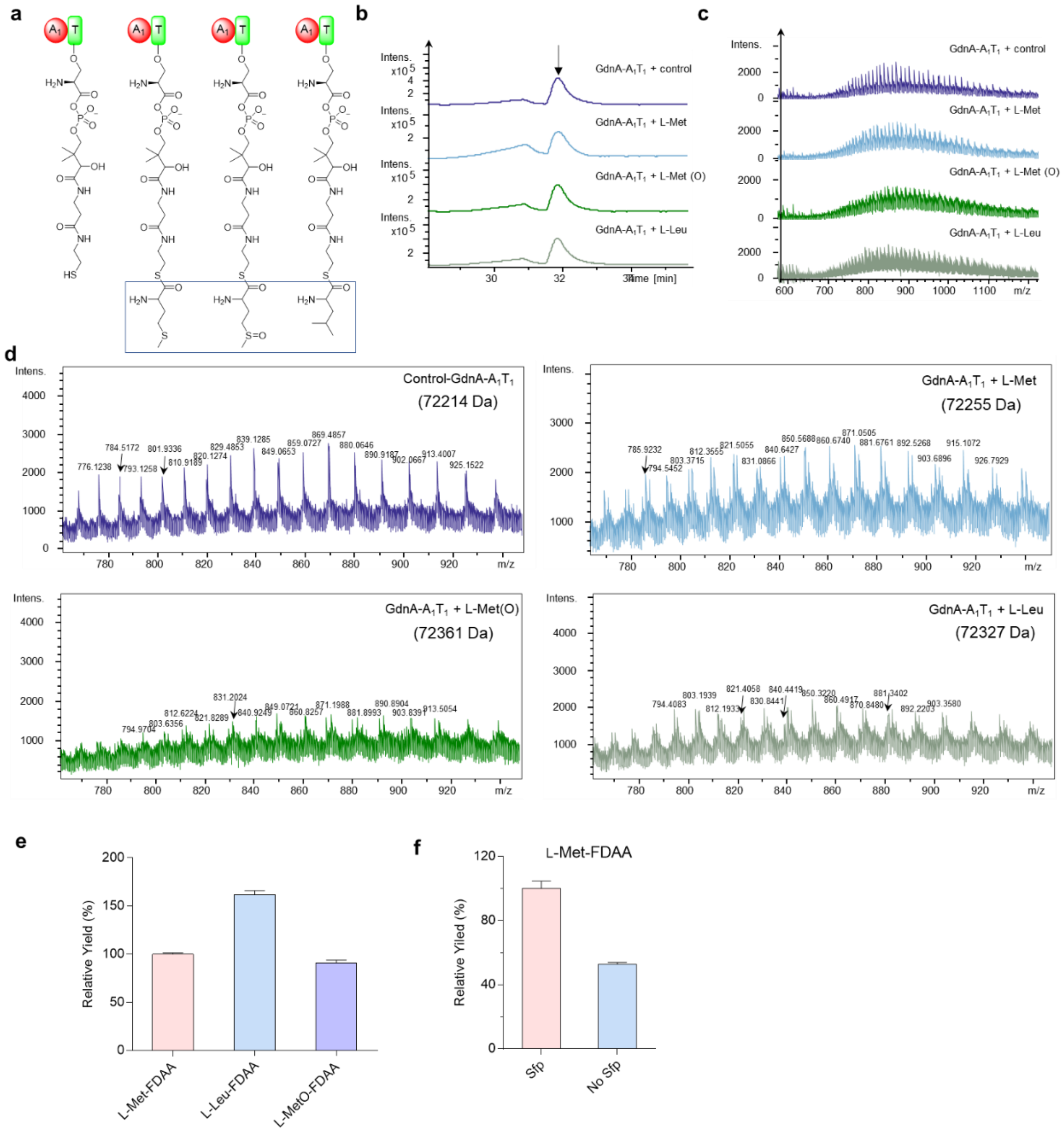

Supplementary Fig. 24. HPLC-MS analysis of proteins A_1T_1 or CsA_1T_1 reacting with substrates. **a.** Structures of A_1T_1 connected to substrates L-Met, L-Met(O) and L-Leu. **b.** HPLC-MS analysis of protein A_1T_1 reacting with substrates. **c, d.** MS/MS profiles of protein A_1T_1 reacting with substrates. **e.** The yield comparison of three substrates linked T_1 domain of CsA_1T_1 protein purified *E. coli* BAP1 through comparing the yield of hydrolyzed target products derivated with FDAA (L-Met-FDAA, L-Leu-FDAA, L-Met(O)-FDAA). The T_1 linked L-Met was quantified as a reference (100%). Data are presented as mean values ±SD, n=3 biologically independent samples. **f.** The yield comparison of the substrate L-Met linked T_1 domain of CsA_1T_1 protein purified *E. coli* BAP1 through comparing the yield of L-Met-FDAA which was supplied with or without Sfp protein. Data are presented as mean values ±SD, n=3 biologically independent samples. Source data are provided as a Source Data file.

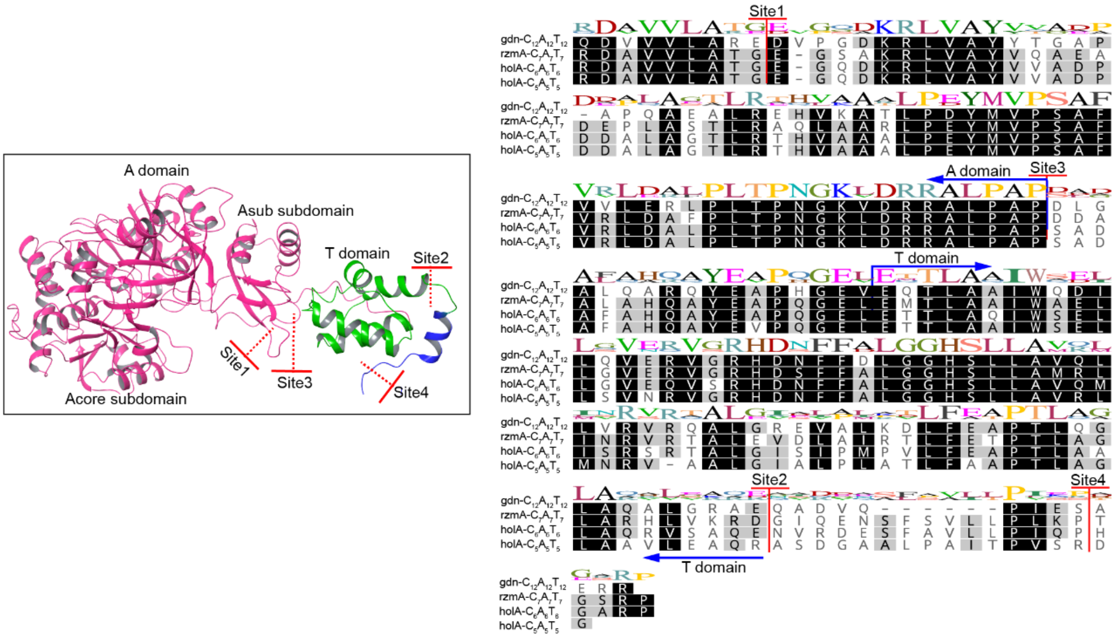

Supplementary Fig. 25. Swapping site of Put unit with *holA* and *rzmA** gene clusters. RzmA*-M1, HolA-M1, and HolA-M6 are swapped in site1, RzmA*-M2, HolA-M2, and HolA-M7 are swapped in site2, HolA-M3 and HolA-M9 are swapped in site1 and site3, HolA-M4 is swapped in site2 and site3, HolA-M8 is swapped in site1 and site4.

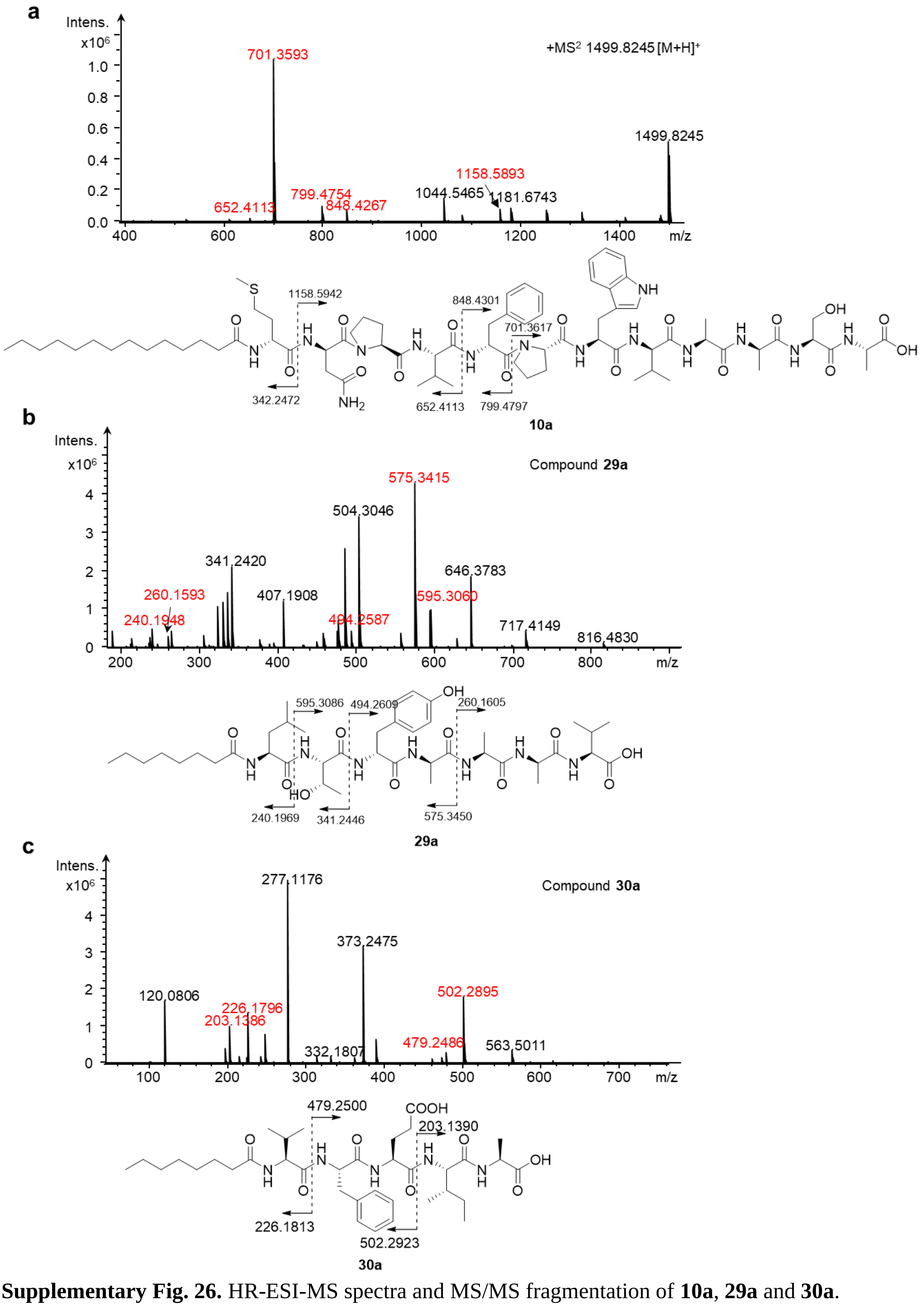

Supplementary Fig. 26. HR-ESI-MS spectra and MS/MS fragmentation of **10a**, **29a** and **30a**.

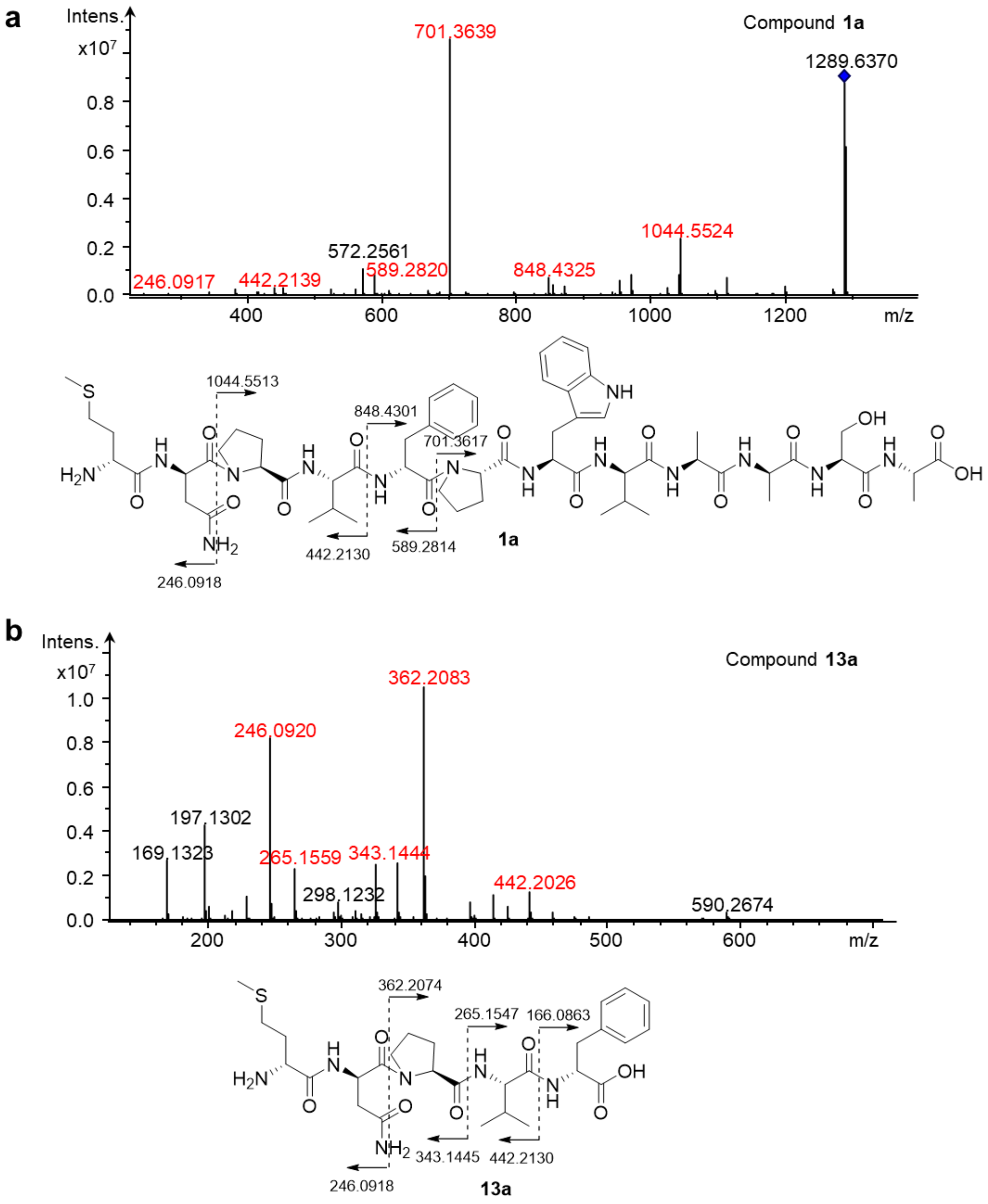

Supplementary Fig. 27. HR-ESI-MS spectra and MS/MS fragmentation of **1a** and **13a**.

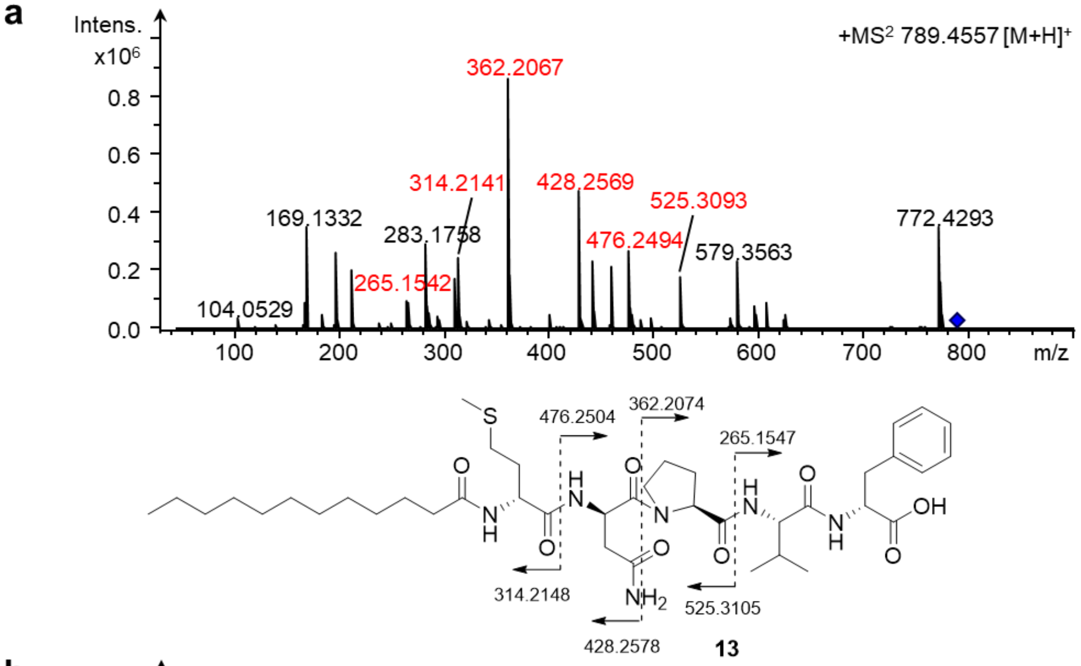

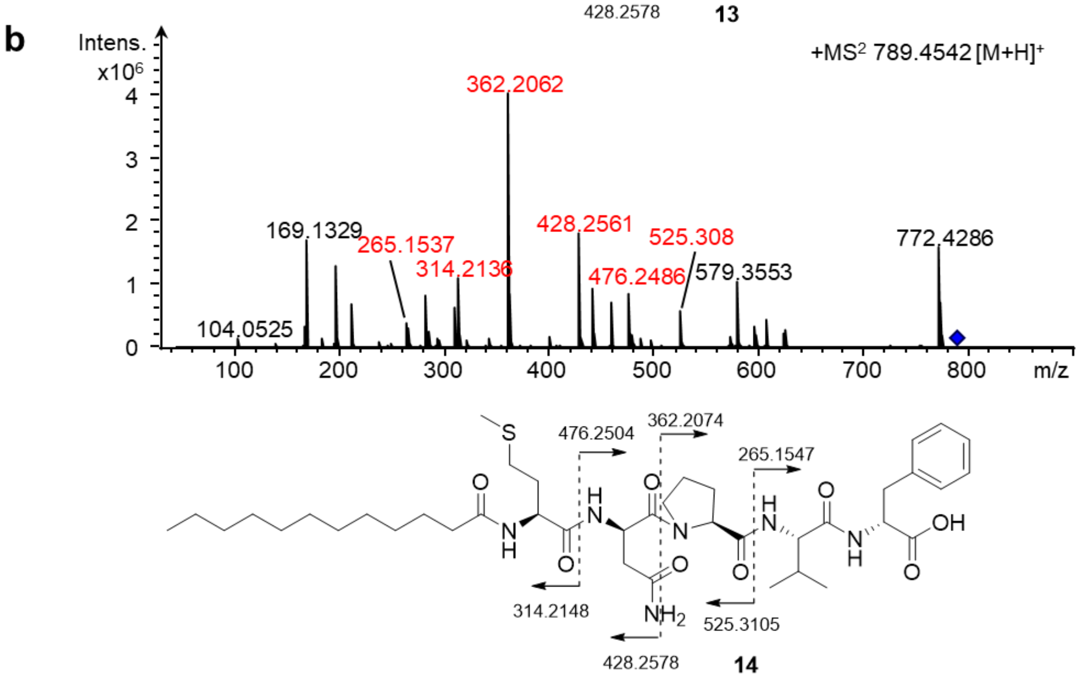

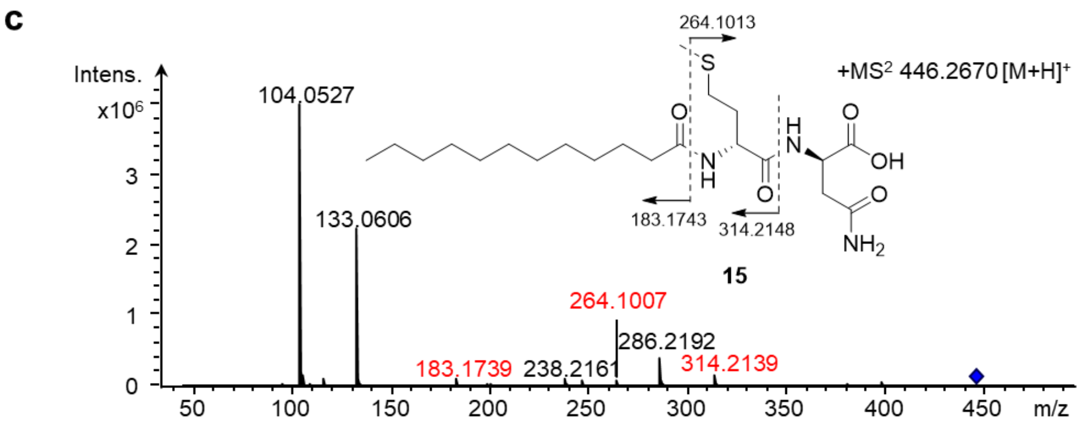

Supplementary Fig. 28. HR-ESI-MS spectra and MS/MS fragmentation of **13**, **14** and **15**.

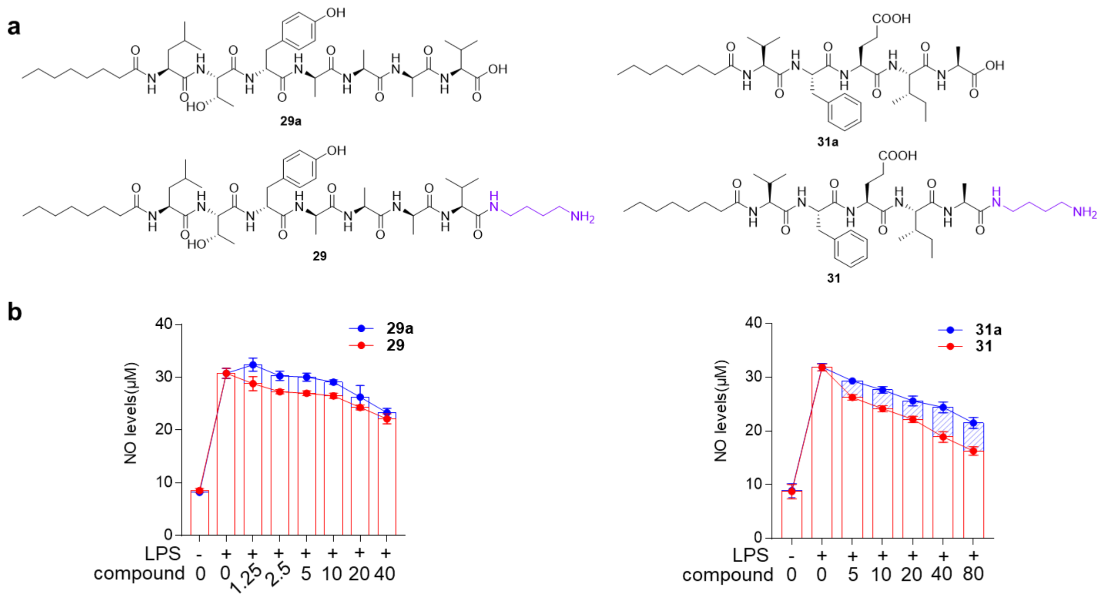

Supplementary Fig. 29. The structures and anti-inflammatory bioactivity of lipopeptides. **a.** Structures of lipopeptides **29a**, **29**, **31a**, and **31**. **b.** The effects of compounds on NO production in LPS-induced RAW264.7 cells between **29**, **31** and each Put-lacking derivatives **29a**, **31a**. NO production in the absence of LPS was used as negative control. LPS-stimulated NO production in the absence of compounds was used as the positive control. Data are presented as mean values ±SD, n=3 biologically independent samples. Source data are provided as a Source Data file.

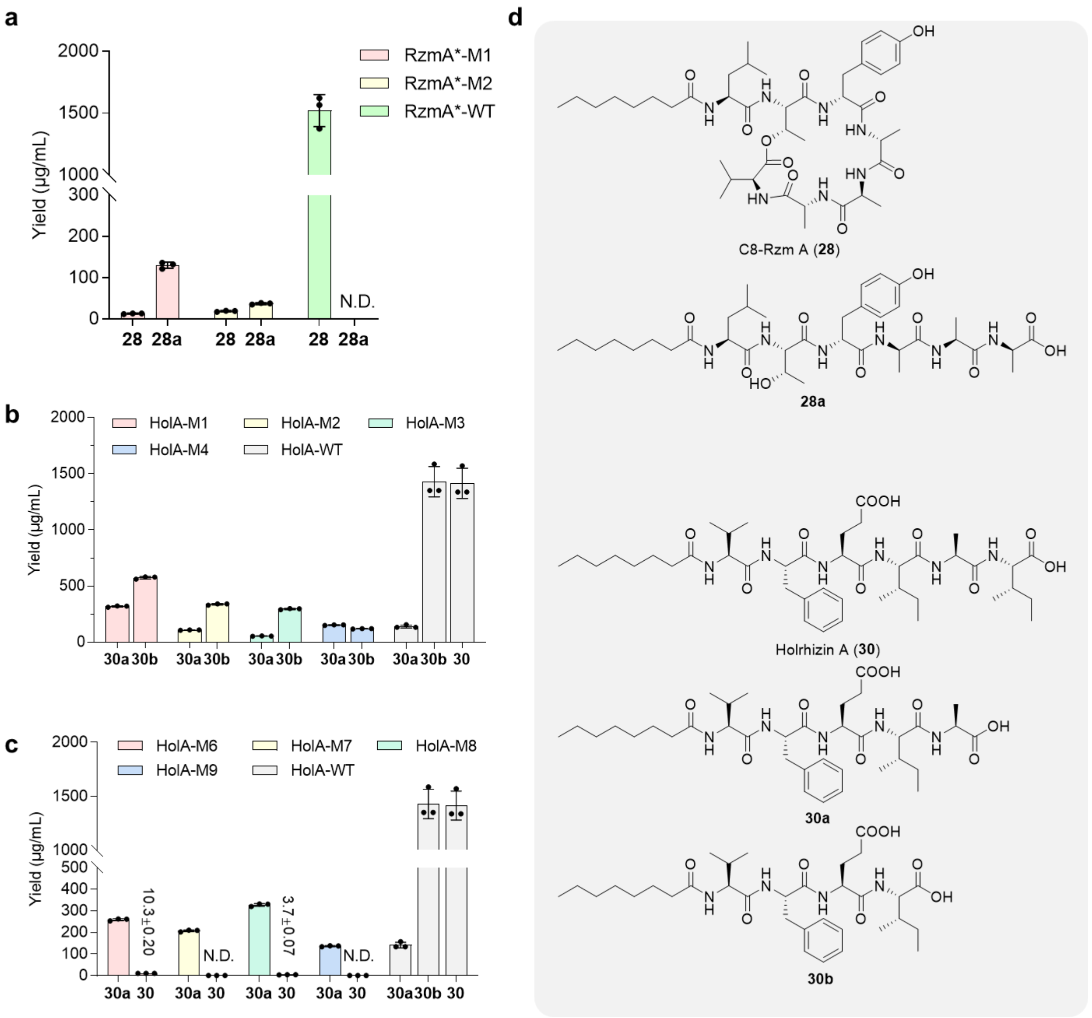

Supplementary Fig. 30. Yield comparison of the other intermediates from engineering *rzmA** and *holA* BGCs between wild type and mutants. **a.** Yield comparsion of **28** and the intermediate **28a** between wild type and two mutants (RzmA*-M1 and RzmA*-M2). The absolute yield of related compounds was determined using the standard curve of **28** and **29a**, respectively. **b**, **c.** Yield comparsion of **30** and the intermediates **30a** and **30b** between wild type and eight mutants. HolA-M5 did not produce any products. The absolute yield of related compounds was determined using the standard curve of **30a**. **d.** Structures of C8-Rzm A, Holrhizin A and their corresponding intermediates. Data are presented as mean values ±SD, n=3 biologically independent samples. N.D. indicates no products. Source data are provided as a Source Data file.

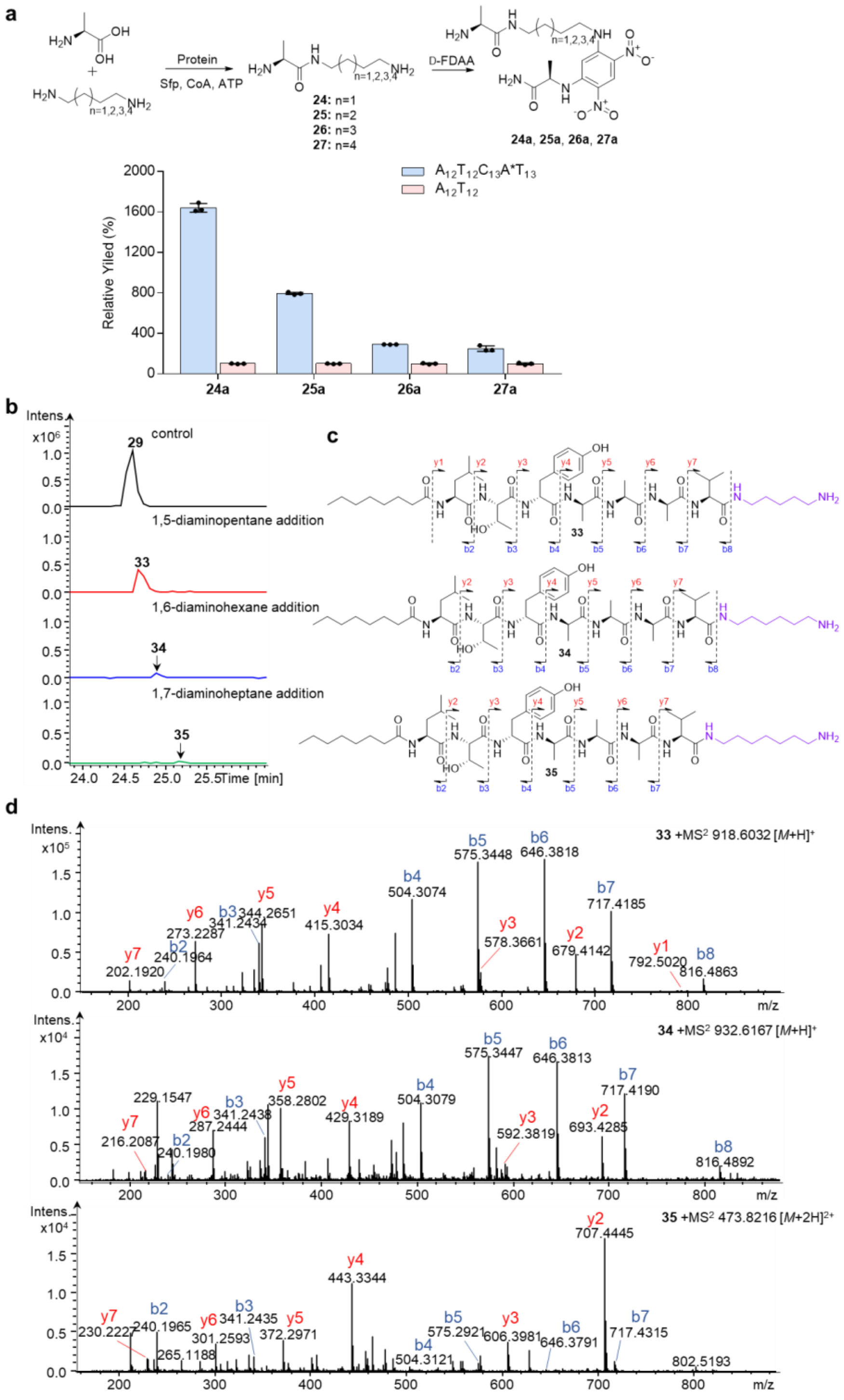

Supplementary Fig. 31. Identification of C8-RzmA's derivatives containing different diamines in RzmA-M2. **a.** *In vitro* bioactivity assays of L-Ala condensing with Put, 1,5-diaminopentane, 1,6-diaminohexane, and 1,7-diaminoheptane mediated by proteins $A_{12}T_{12}C_{13}A^{*}T_{13}$ and $A_{12}T_{12}$. The yield of **24a**, **25a**, **26a** and **27a** generated from the protein $A_{12}T_{12}$ was quantified as a reference (100%). Data are presented as mean values ±SD, n=3 biologically independent samples. Source data are provided as a Source Data file. **b.** HPLC-MS analysis of crude extracts from mutants GB05MtaA:C8-RzmA-M2 with the addition of 1,7-diaminoheptane, 1,6-diaminohexane, and 1,5-diaminopentane in the medium, respectively. **c**, **d.** The structures and MS/MS fragmentation of compounds **33-35**.

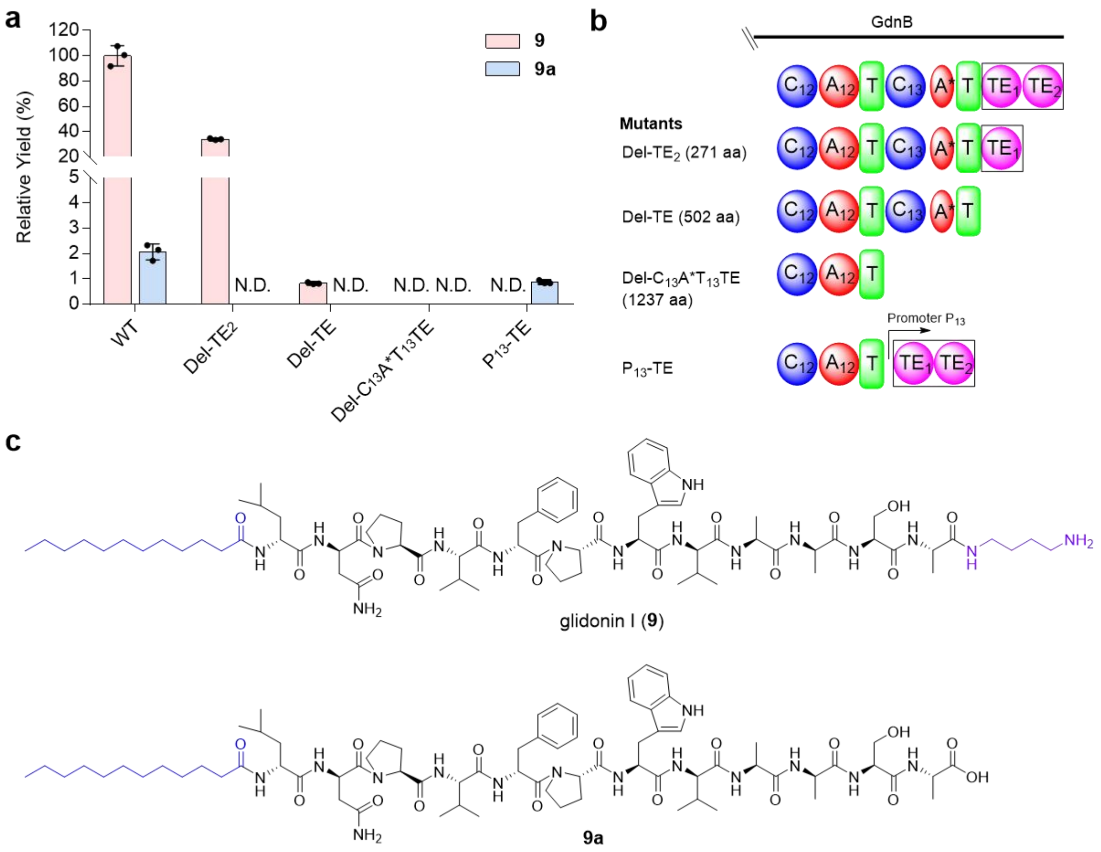

Supplementary Fig. 32. Analysis of TE domain influencing the glidonin biosynthesis. **a.** HPLC-MS analysis of crude extract of four mutants compared with wild type. The yield of **9** generated from the wild type strain DSM 7029:P_{Apra}-*gdn* was quantified as a reference (100%). Data are presented as mean values ±SD, n=3 biologically independent samples. N.D. indicates no products. N.D. indicates no products. Source data are provided as a Source Data file. **b.** The diagram of different mutants. Promoter P_{13} was from DSM 7029 genome. **c.** Structures of **9** and intermediate **9a**.

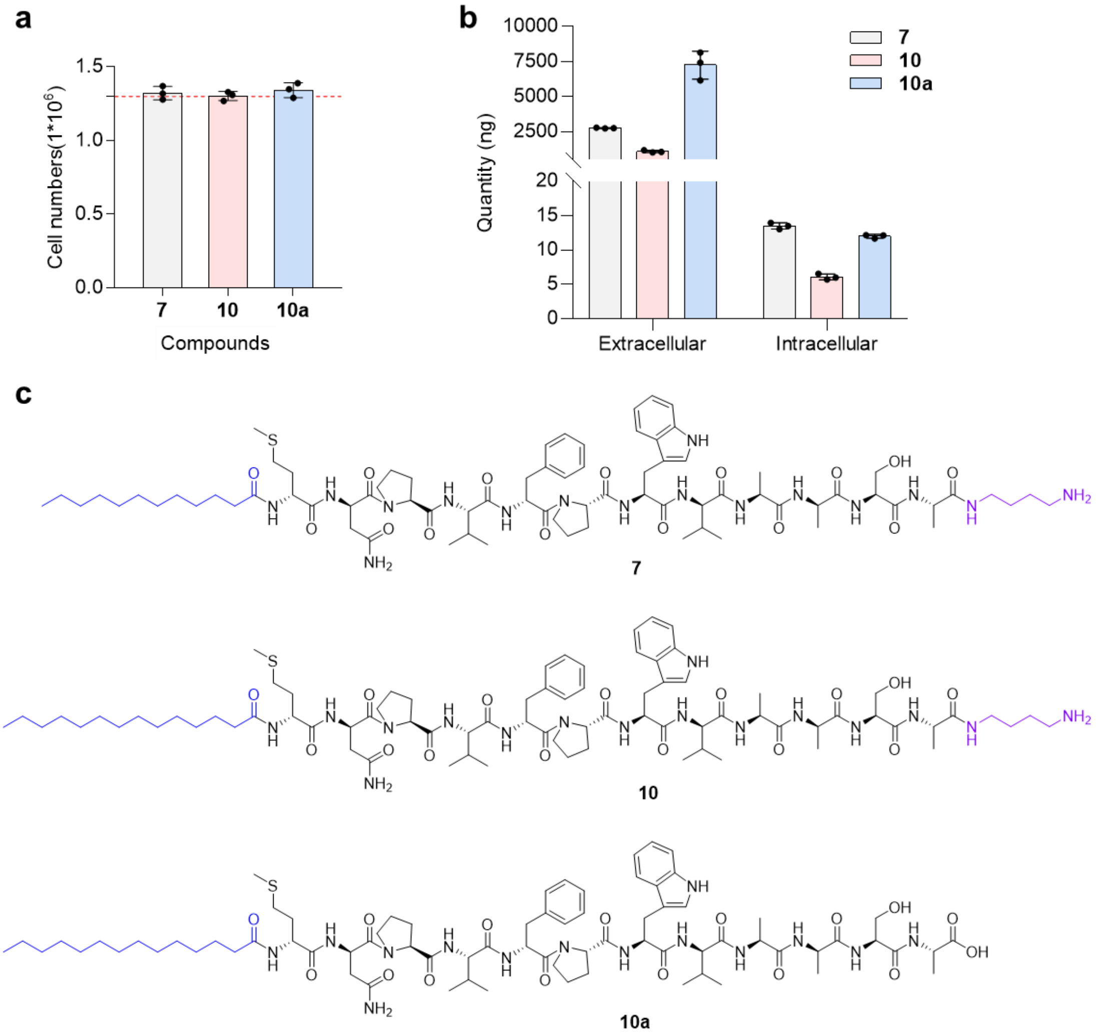

Supplementary Fig. 33. Analysis of hepatoma cell line HepG2 treated with compounds **7** (C12 acyl), **10** (C14 acyl), and **10a** (C14 acyl and without Put). **a.** Analysis of cell numbers treated with compounds. Data are presented as mean values ±SD, n=3 biologically independent samples. Source data are provided as a Source Data file. **b.** LC-MS analysis of extracellular and intracellular compounds. Data are presented as mean values ±SD, n=3 biologically independent samples. **c.** Structures of compounds **7**, **10**, and **10a**.

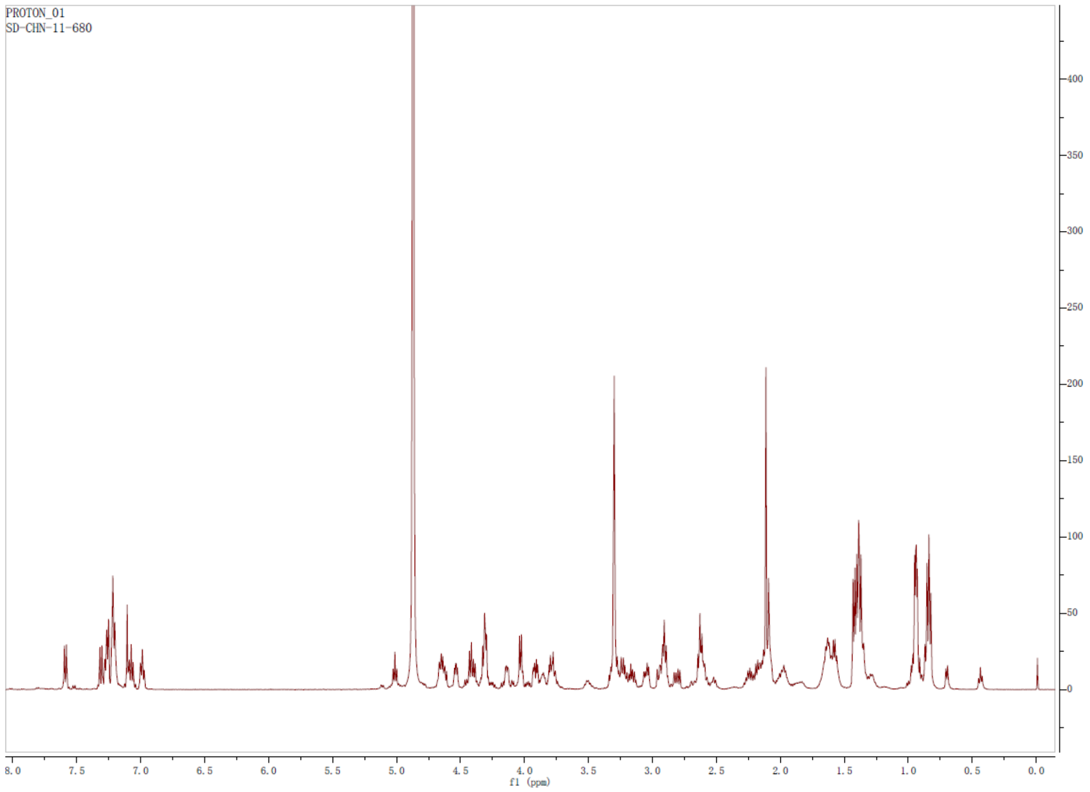

Supplementary Fig. 34. ^{1}H NMR spectrum of glidonin A (**1**) in MeOD-d_4 (500 MHz)

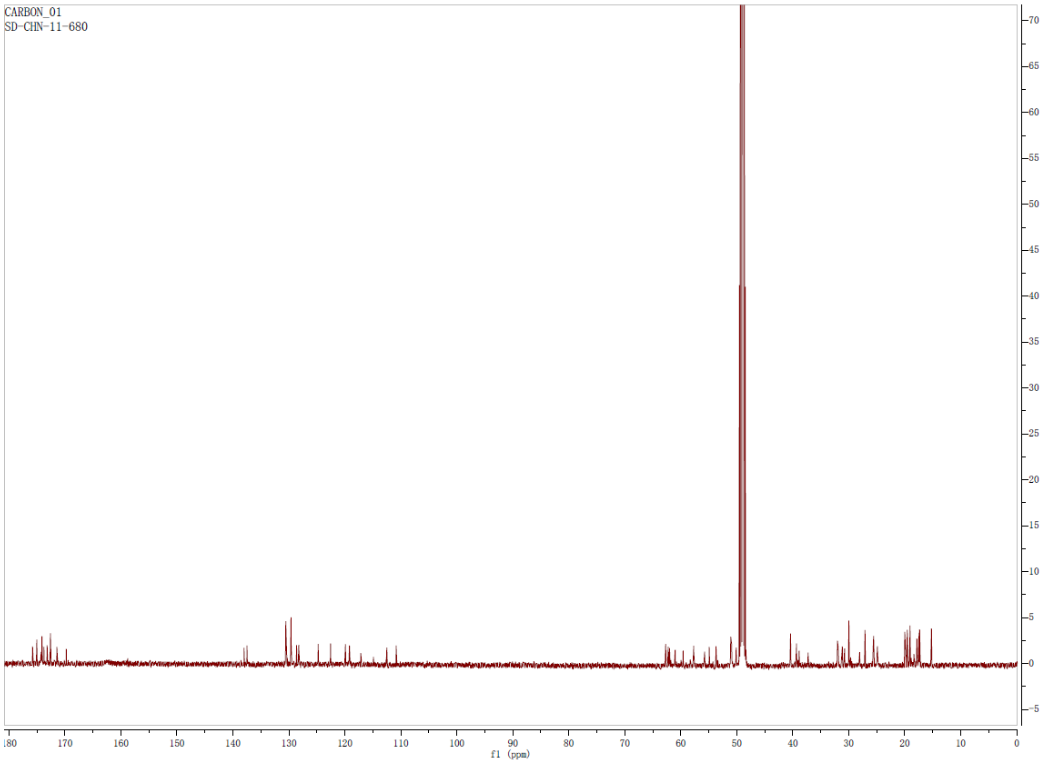

Supplementary Fig. 35. ^{13}C NMR spectrum of glidonin A (**1**) in MeOD-d_4 (125 MHz)

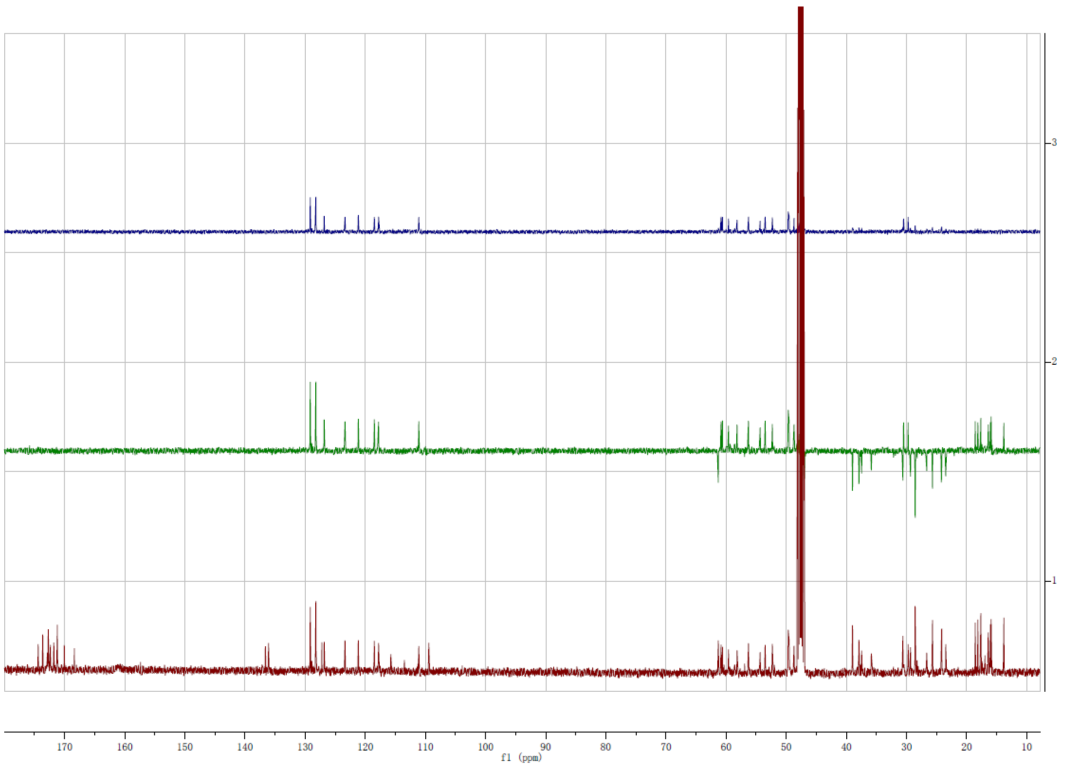

Supplementary Fig. 36. DEPT spectrum of glidonin A (**1**) in MeOD-d_4

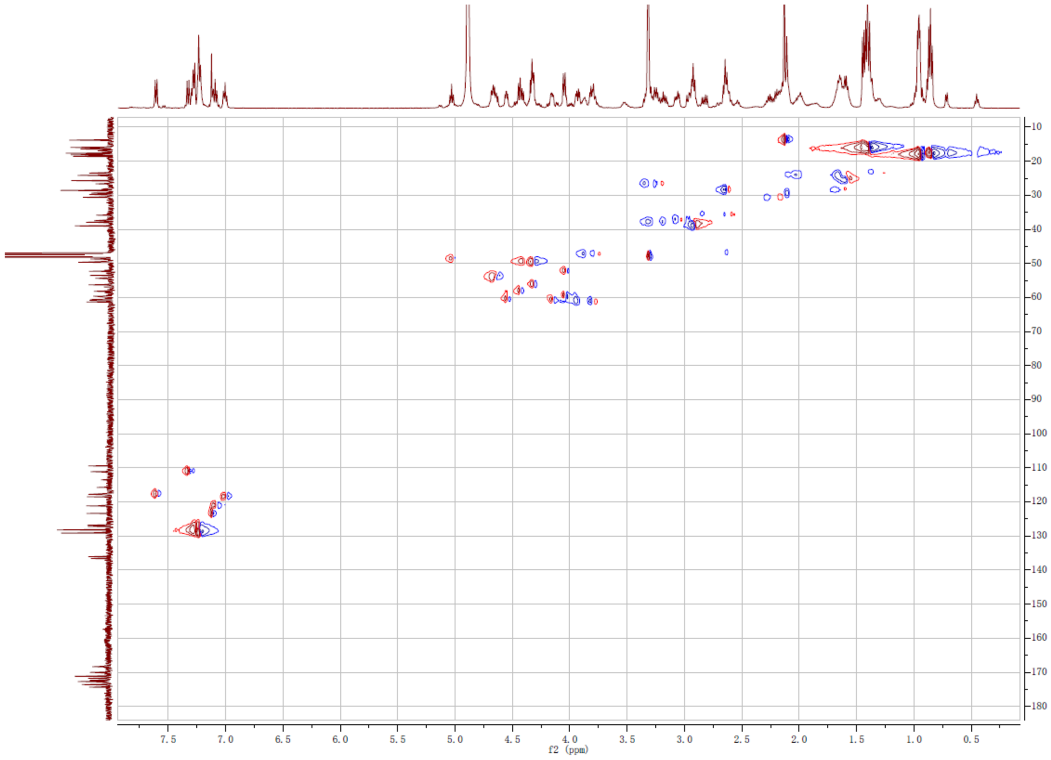

Supplementary Fig. 37. HSQC spectrum of glidonin A (**1**) in MeOD-d_4

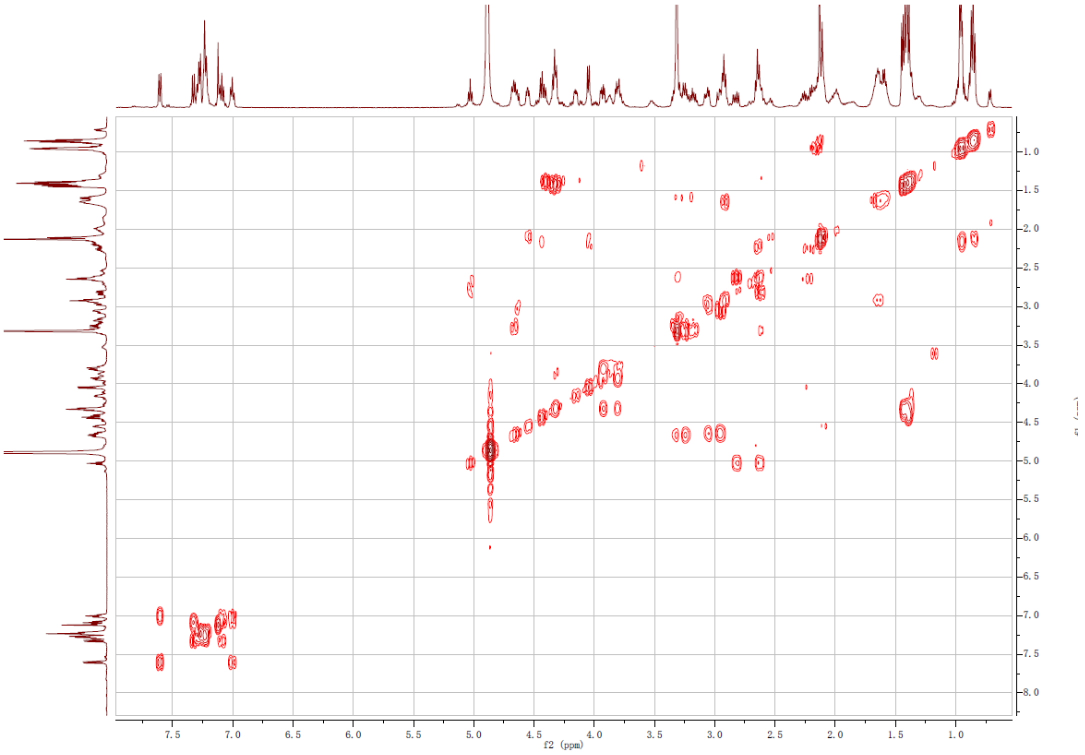

Supplementary Fig. 38. ^{1}H-^{1}H COSY spectrum of glidonin A (**1**) in MeOD-d_4

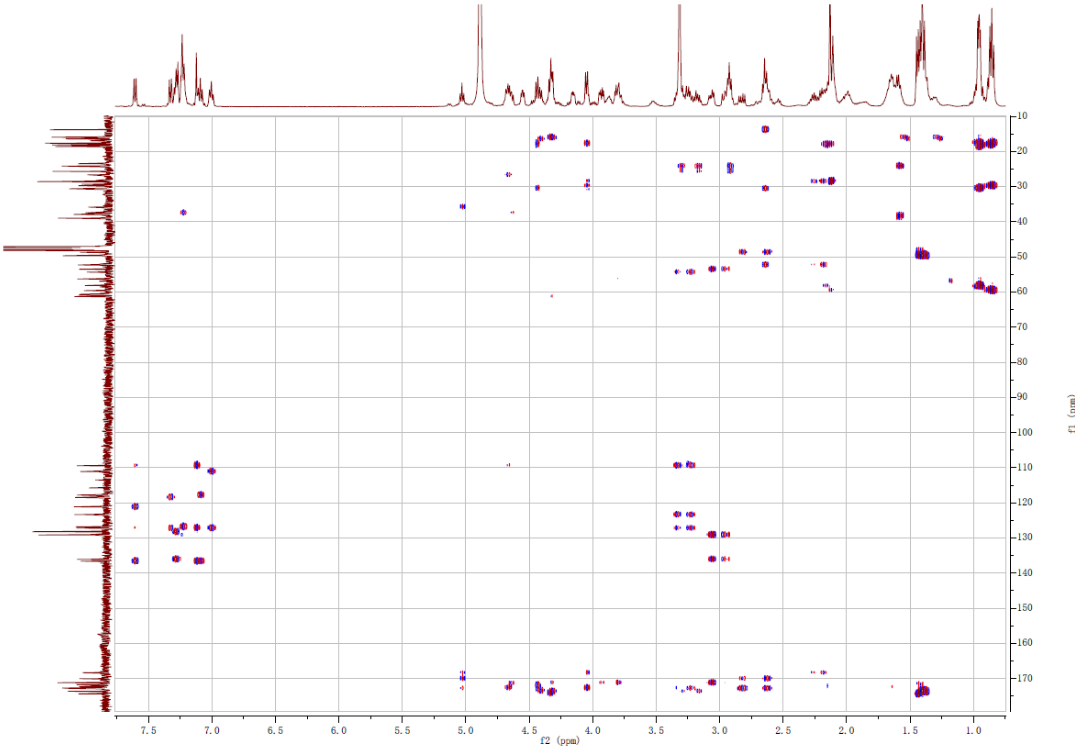

Supplementary Fig. 39. HMBC spectrum of glidonin A (**1**) in MeOD-d_4

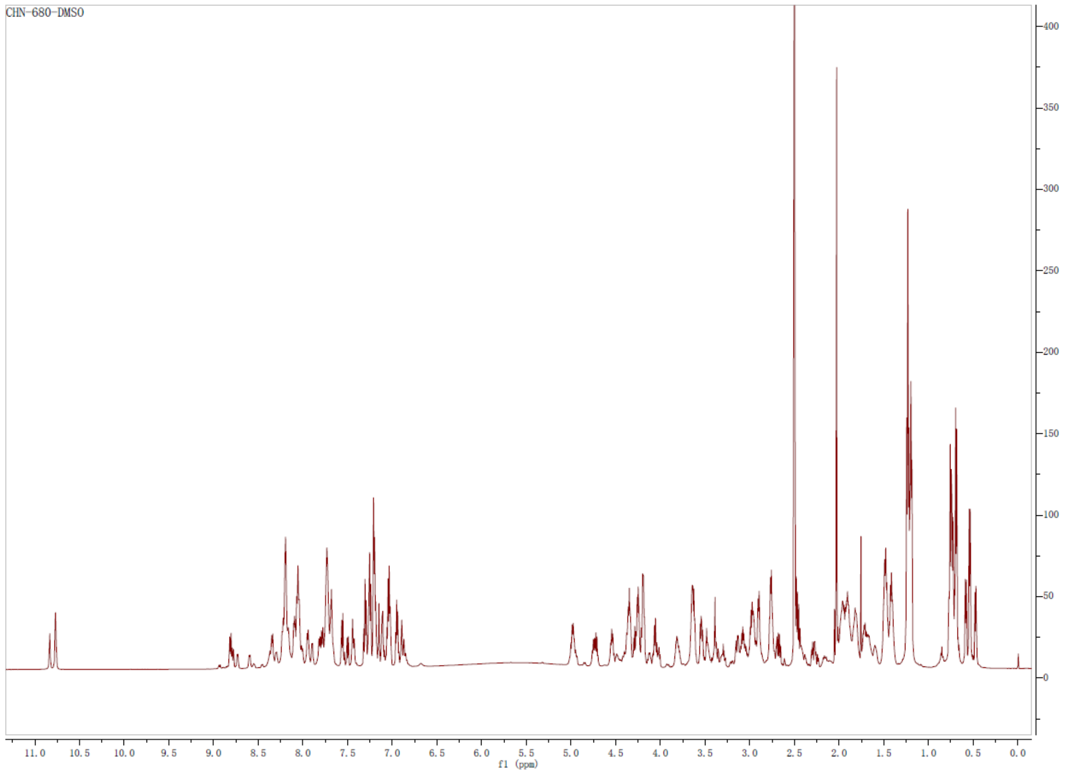

Supplementary Fig. 40. ^{1}H NMR spectrum of glidonin A (**1**) in DMSO-d_6 (600 MHz)

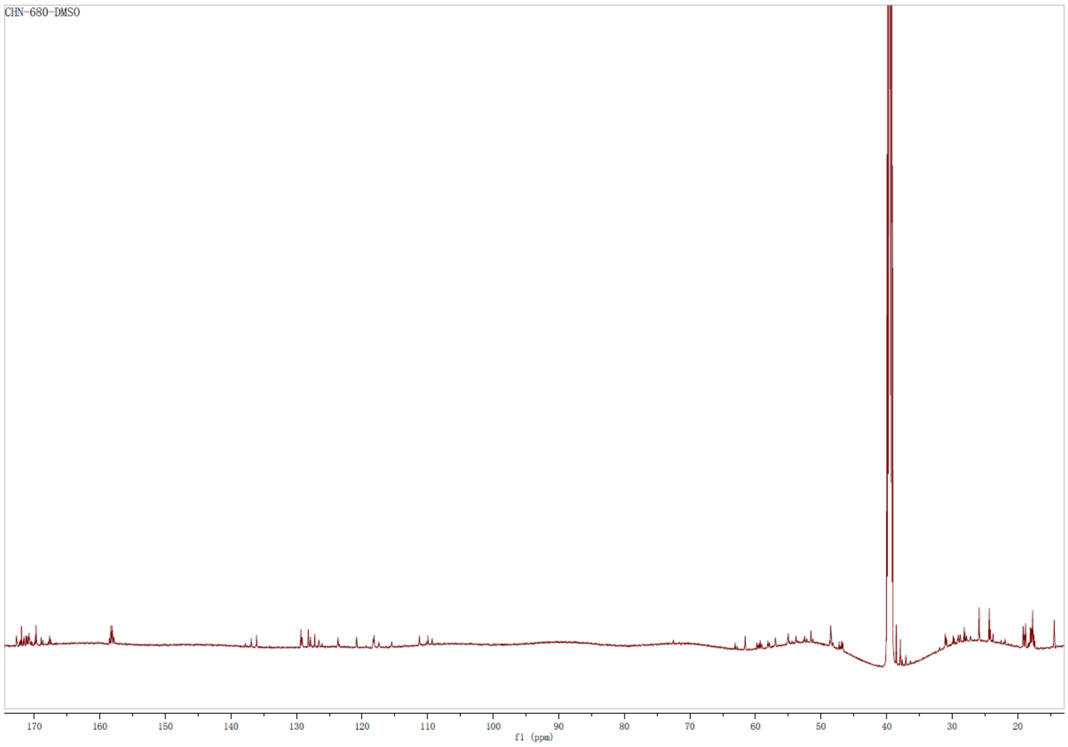

Supplementary Fig. 41. ^{13}C NMR spectrum of glidonin A (**1**) in DMSO-d_6 (150 MHz)

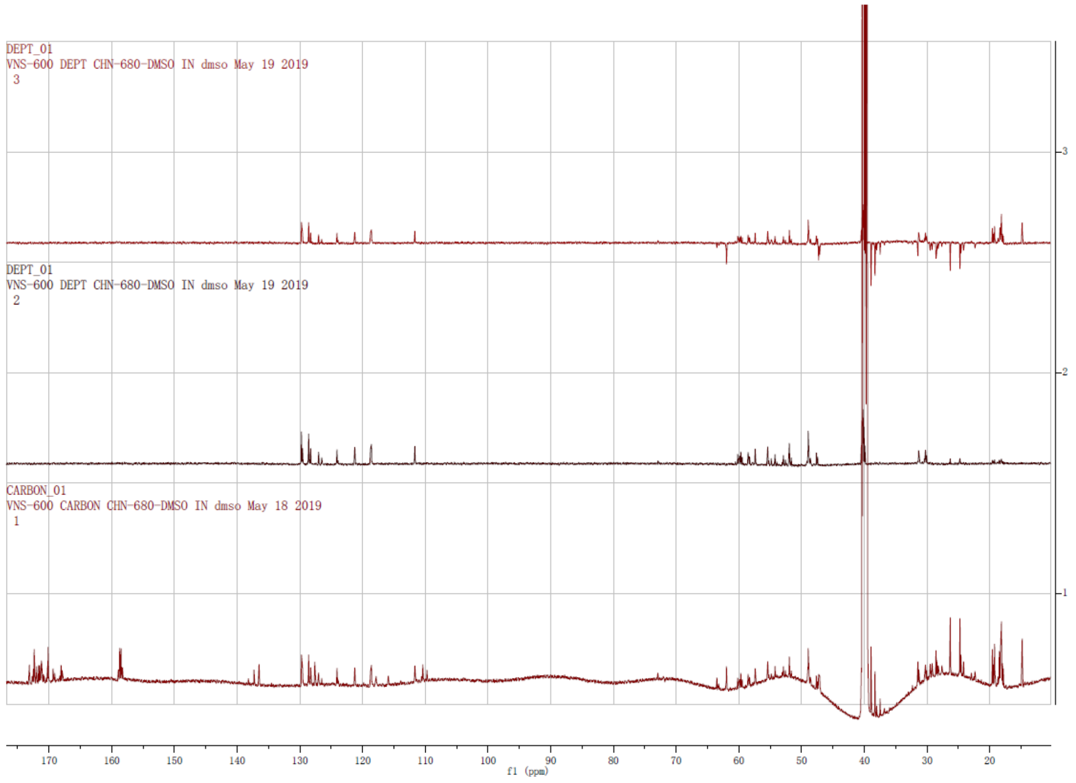

Supplementary Fig. 42. DEPT spectrum of glidonin A (**1**) in DMSO-d_6

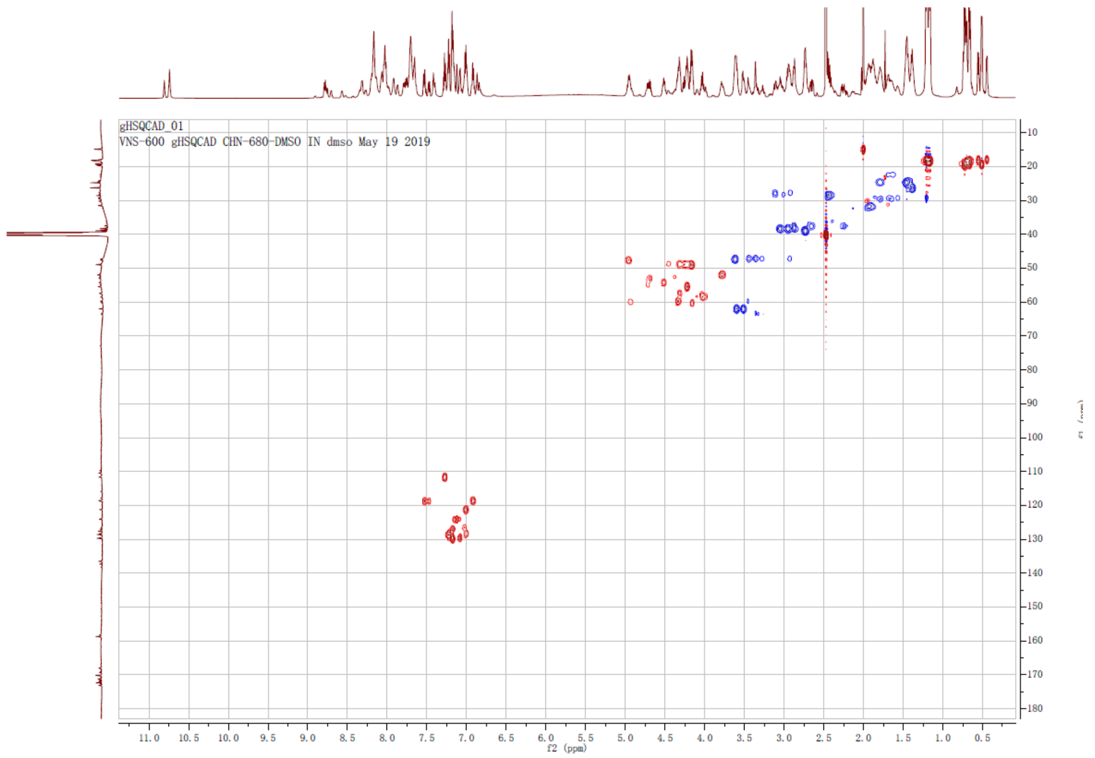

Supplementary Fig. 43. HSQC spectrum of glidonin A (**1**) in DMSO-d_6

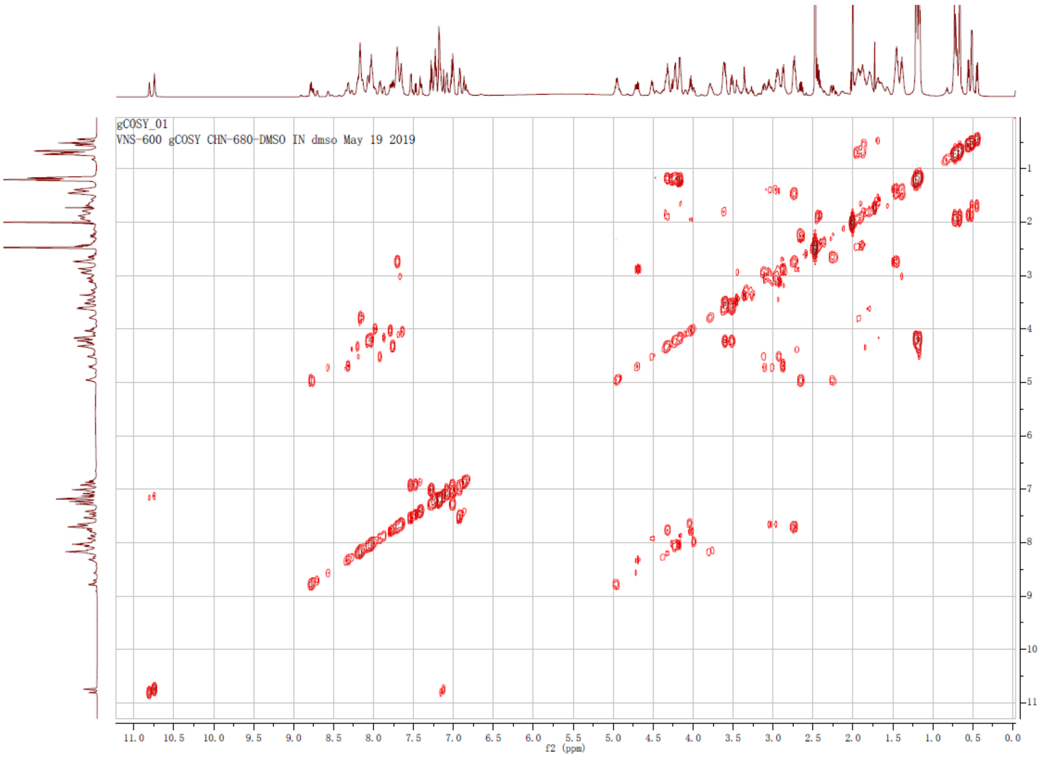

Supplementary Fig. 44. ^{1}H-^{1}H COSY spectrum of glidonin A (**1**) in DMSO-d_6

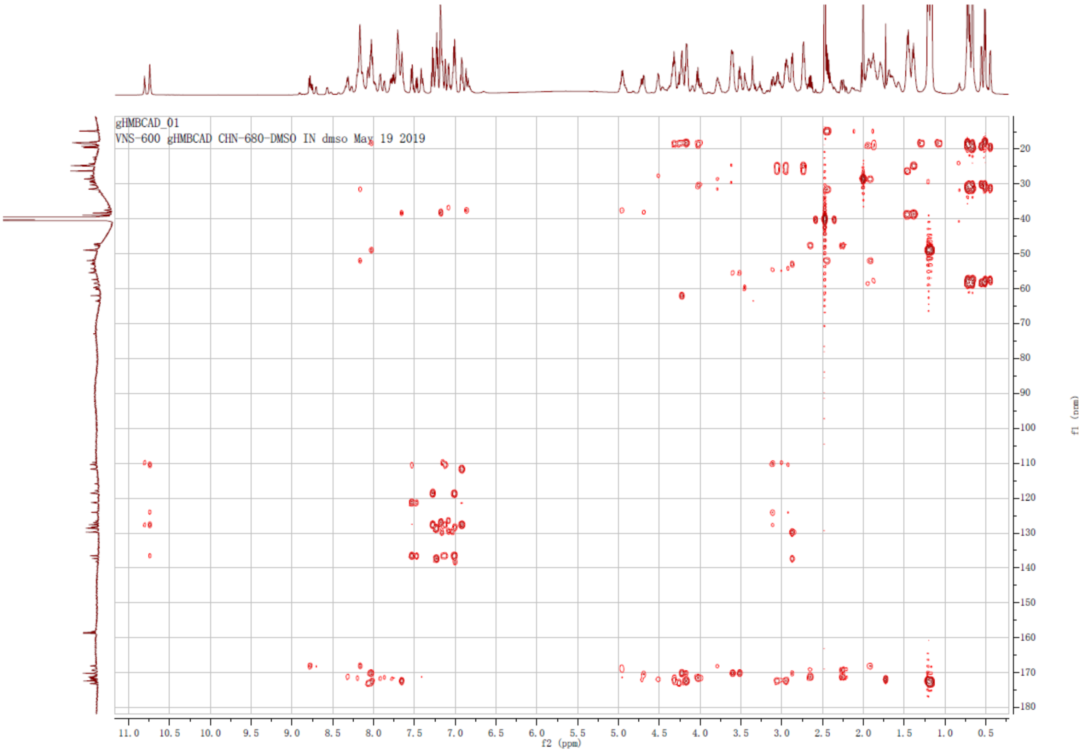

Supplementary Fig. 45. HMBC spectrum of glidonin A (**1**) in DMSO-d_6

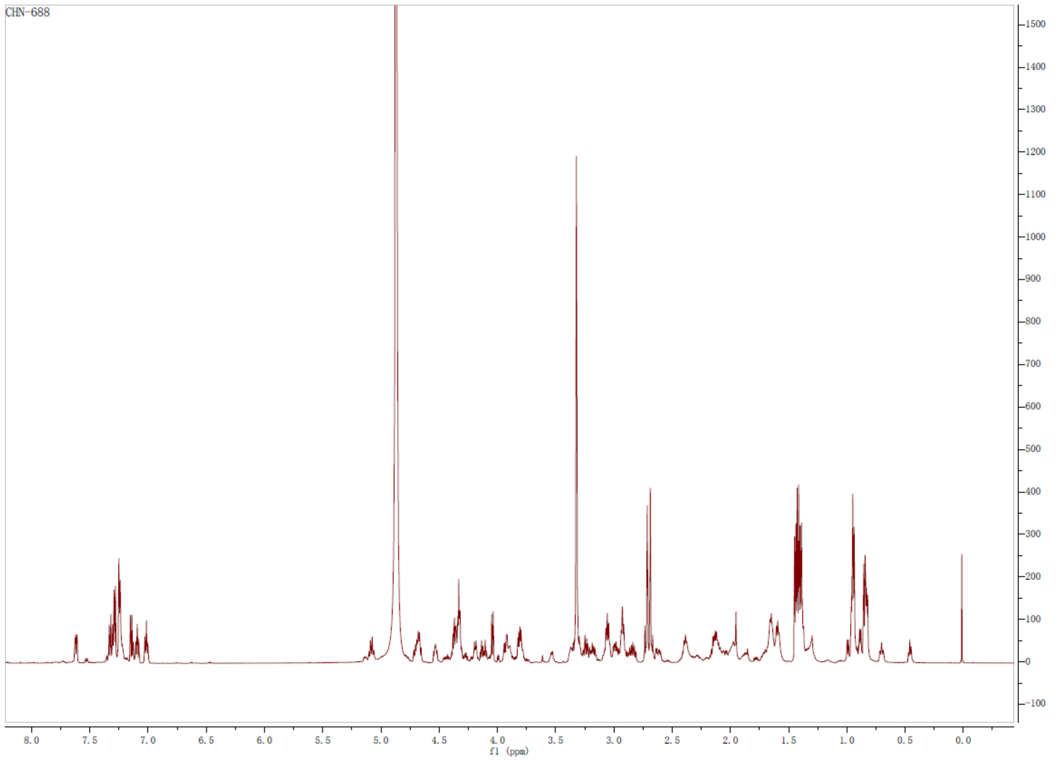

Supplementary Fig. 46. ^{1}H NMR spectrum of glidonin B (**2**) in MeOD-d_4 (600 MHz)

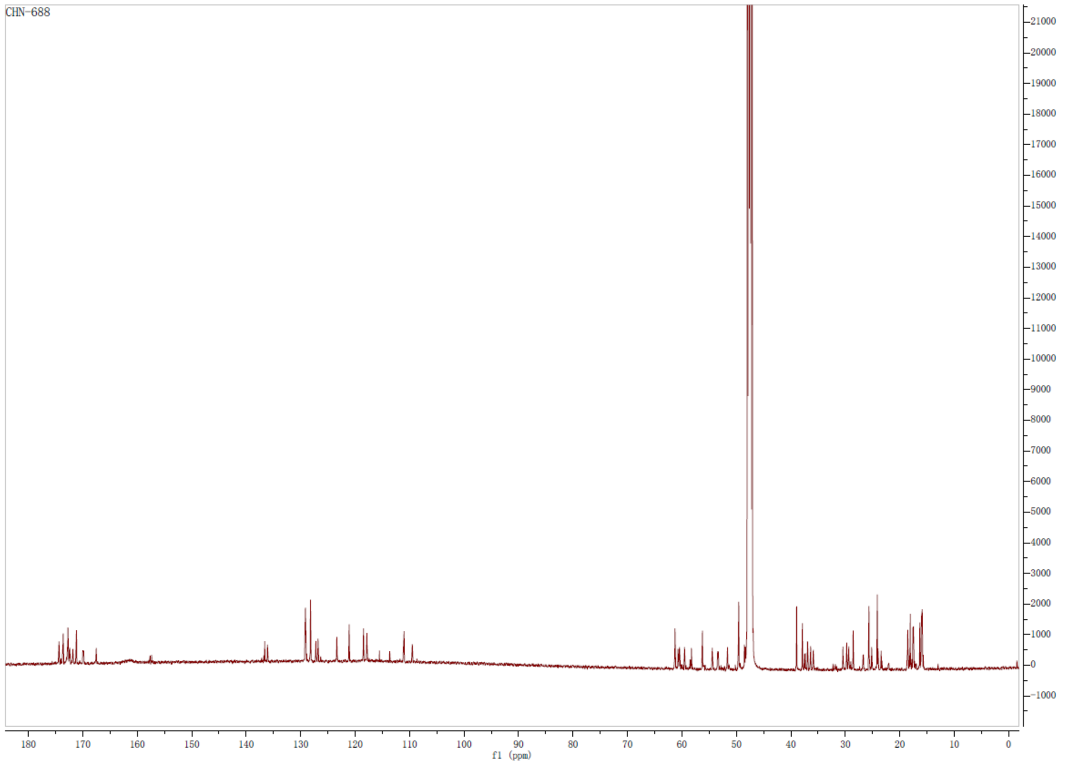

Supplementary Fig. 47. ^{13}C NMR spectrum of glidonin B (**2**) in MeOD-d_4 (150 MHz)

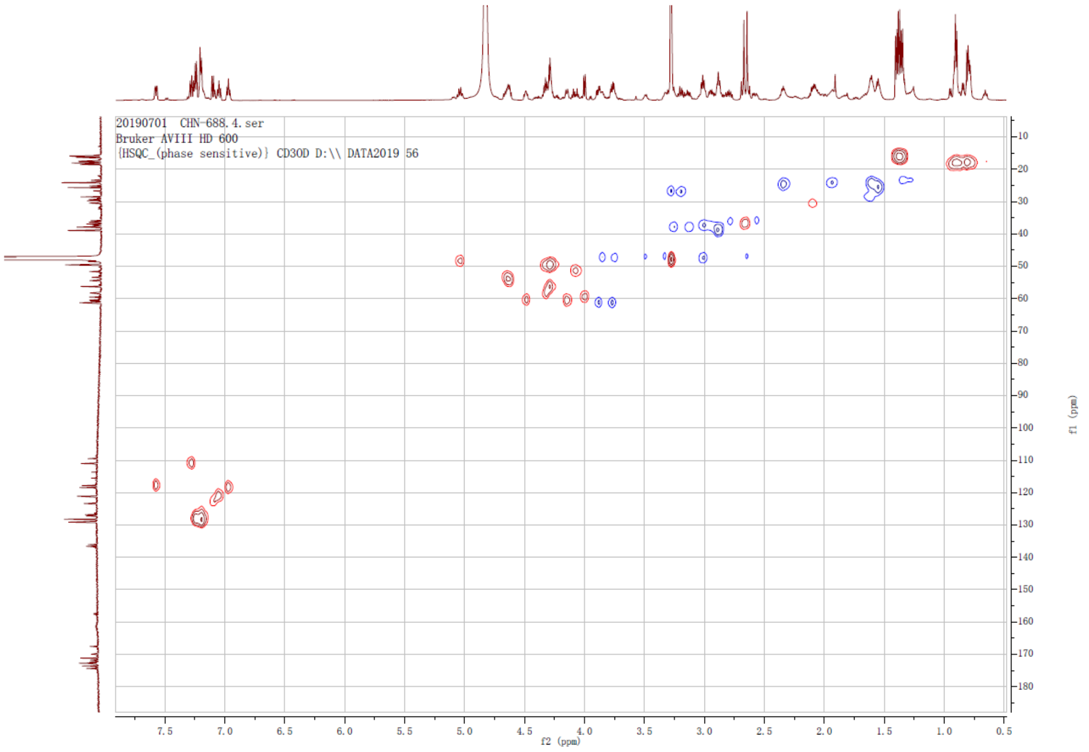

Supplementary Fig. 48. HSQC spectrum of glidonin B (**2**) in MeOD-d_4

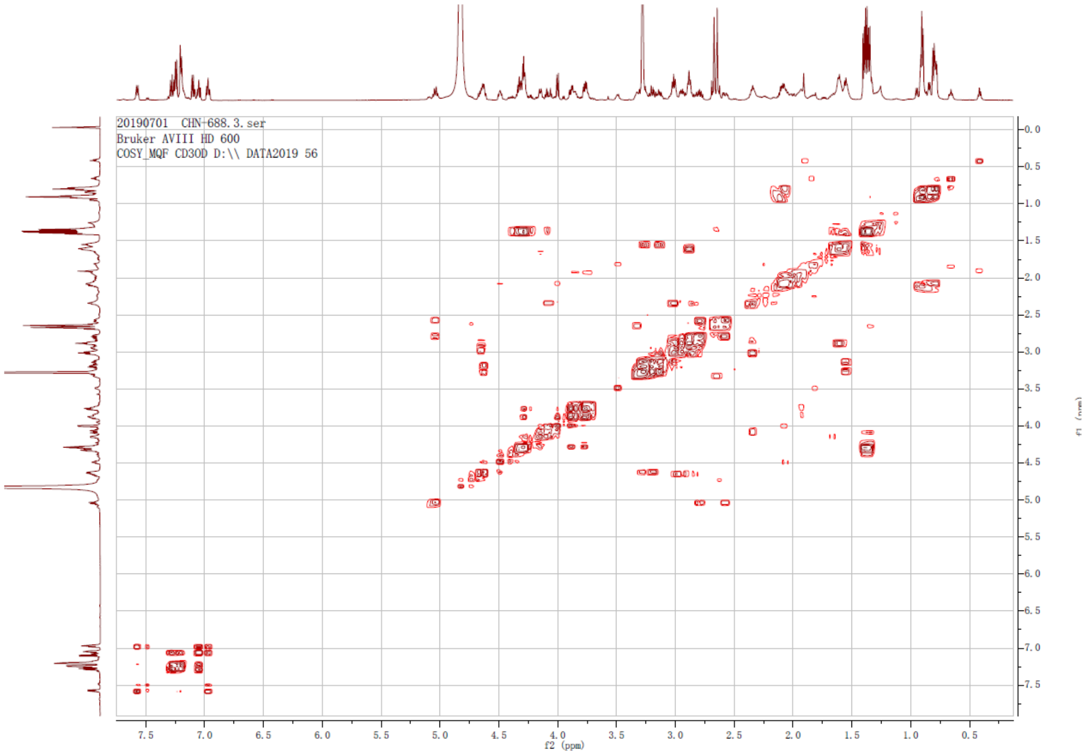

Supplementary Fig. 49. ^{1}H-^{1}H COSY spectrum of glidonin B (**2**) in MeOD-d_4

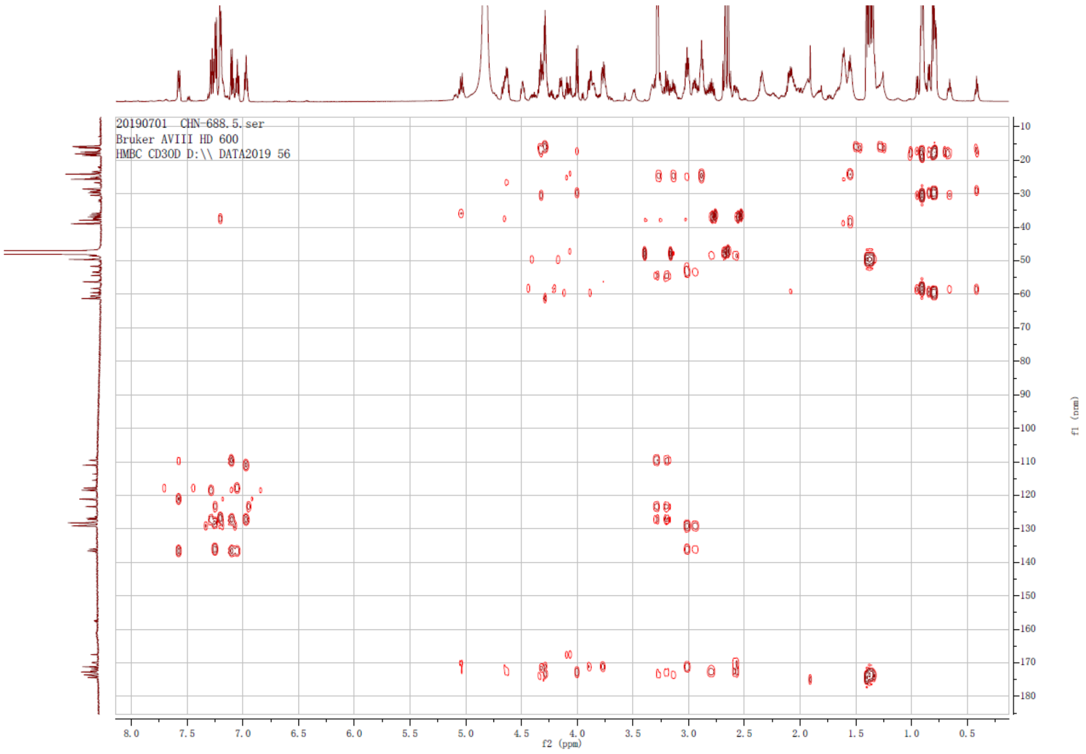

Supplementary Fig. 50. HMBC spectrum of glidonin B (**2**) in MeOD-d_4

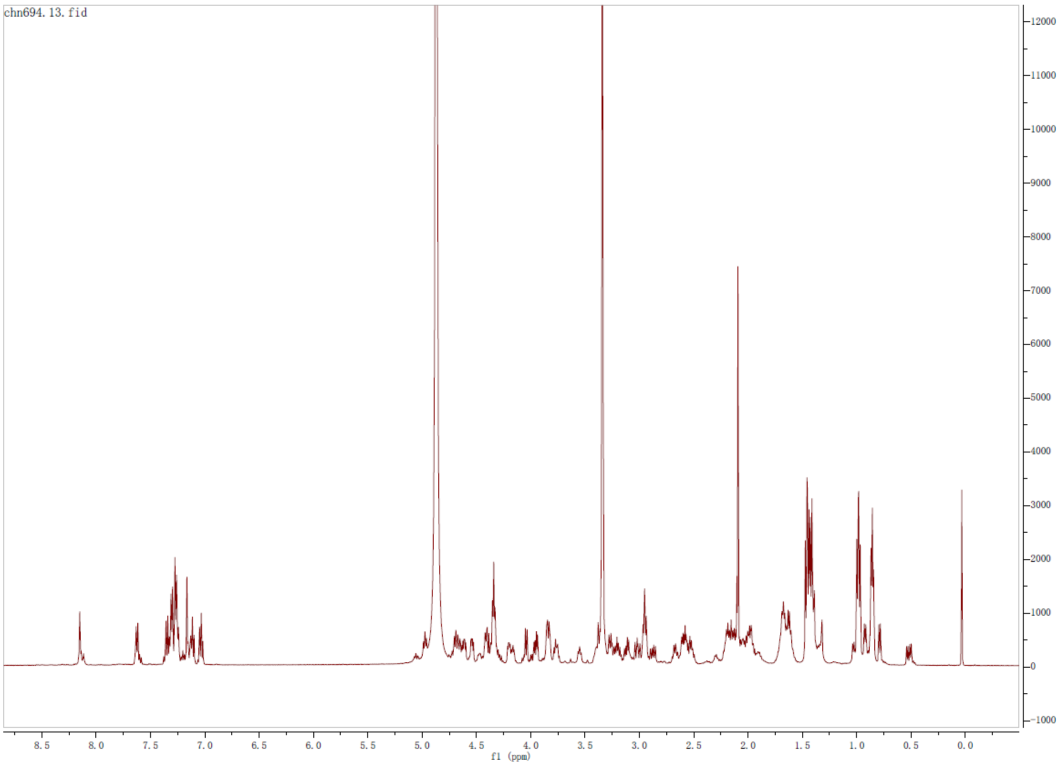

Supplementary Fig. 51. ^{1}H NMR spectrum of glidonin D (**4**) in MeOD-d_4 (500 MHz)

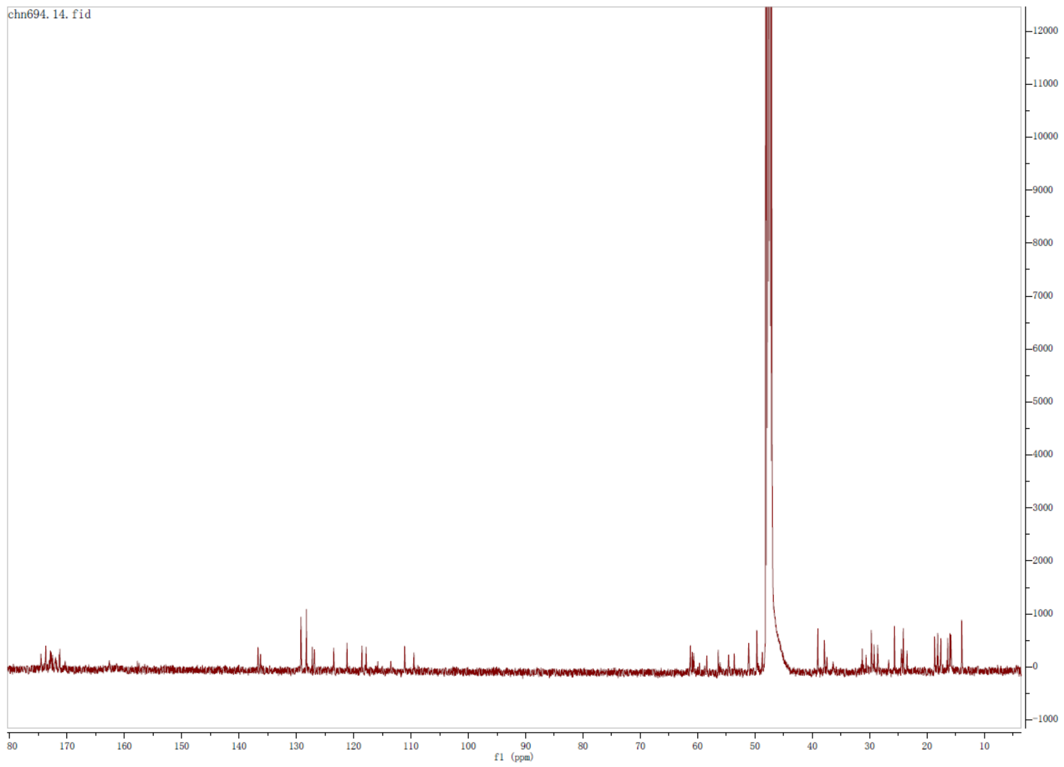

Supplementary Fig. 52. ^{13}C NMR spectrum of glidonin D (**4**) in MeOD-d_4 (125 MHz)

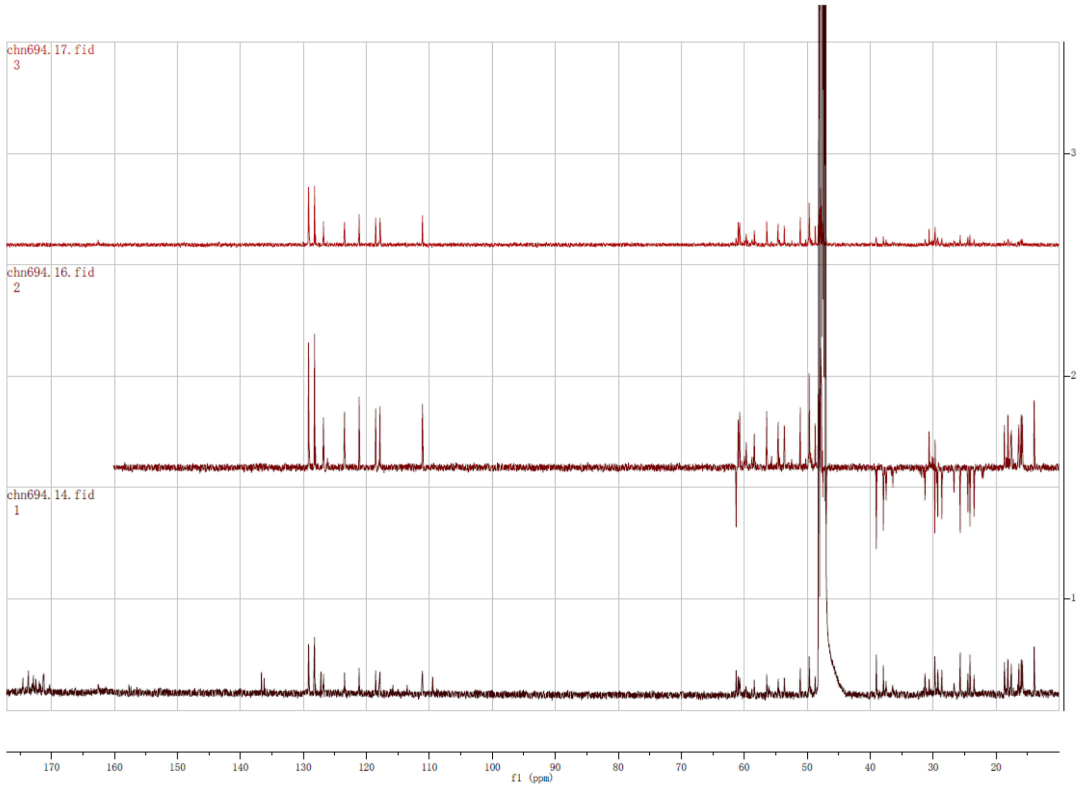

Supplementary Fig. 53. DEPT spectrum of glidonin D (**4**) in MeOD-d_4

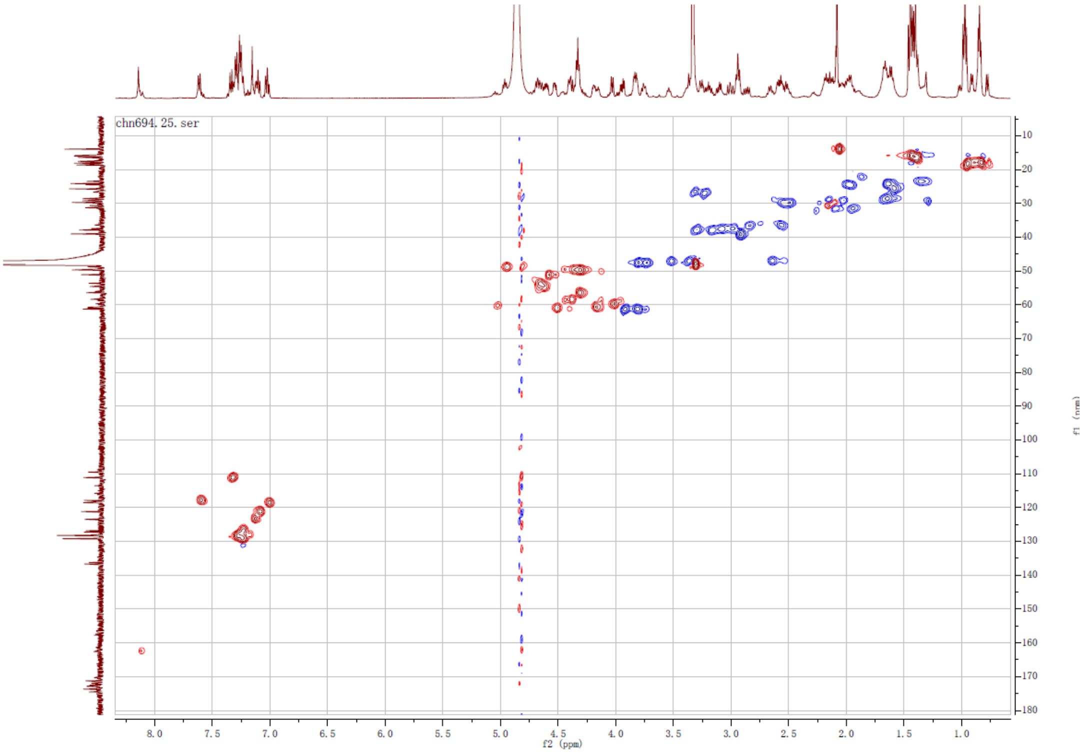

Supplementary Fig. 54. HSQC spectrum of glidonin D (**4**) in MeOD-d_4

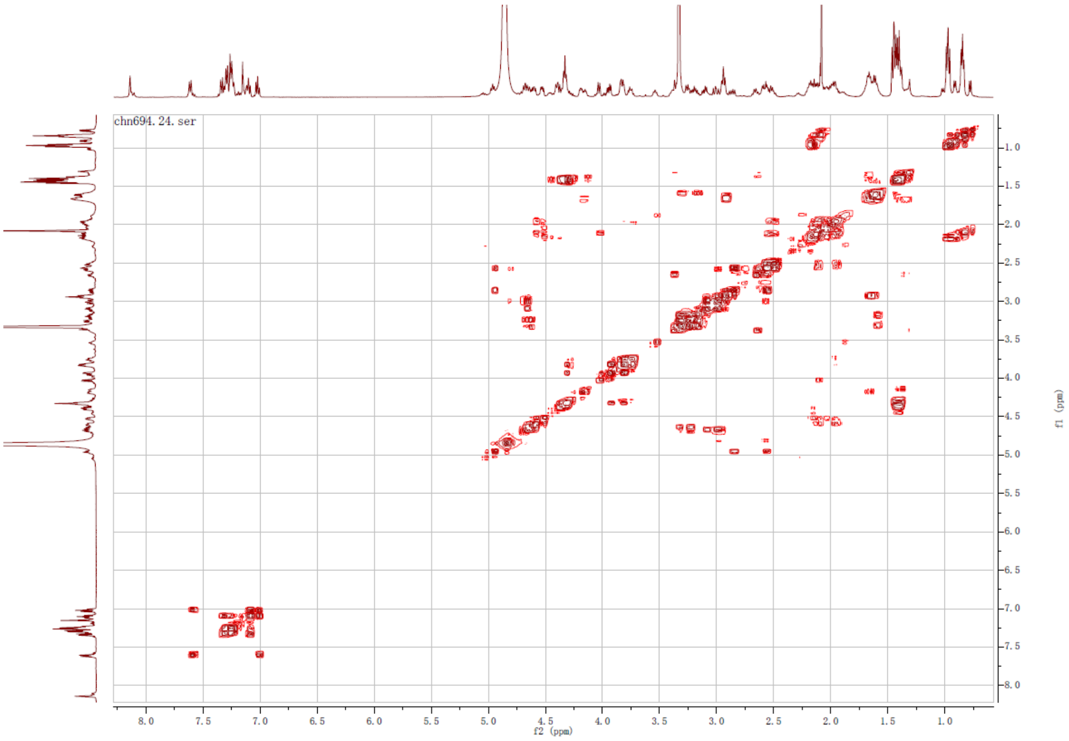

Supplementary Fig. 55. ^{1}H-^{1}H COSY spectrum of glidonin D (**4**) in MeOD-d_4

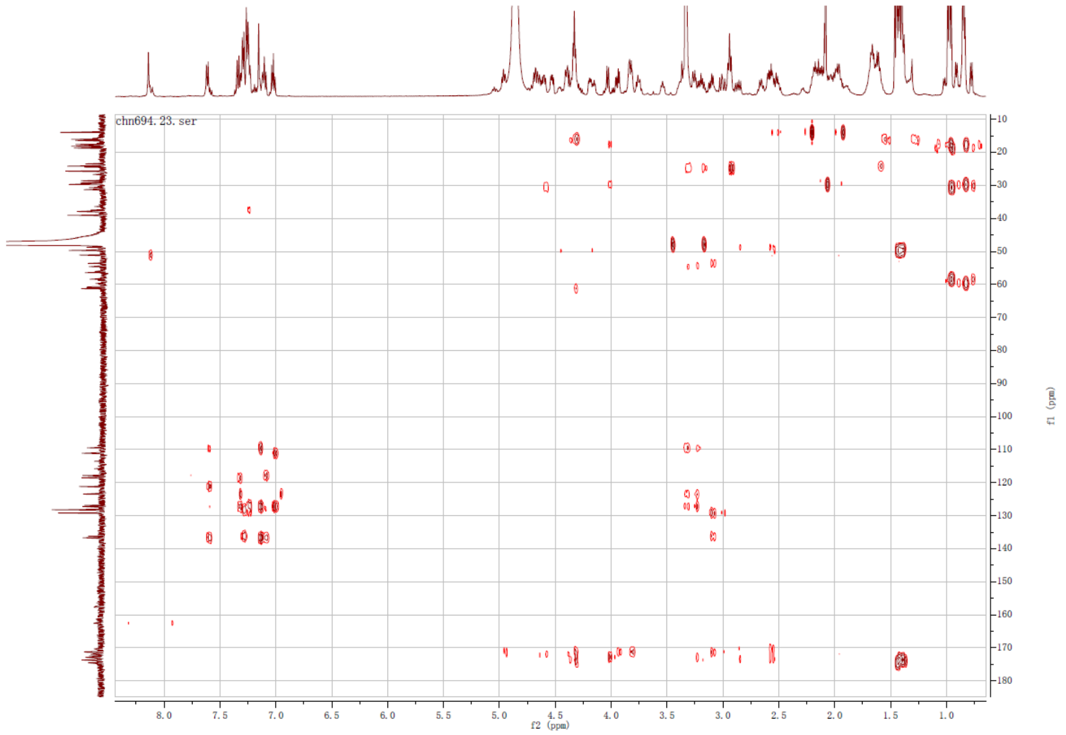

Supplementary Fig. 56. HMBC spectrum of glidonin D (**4**) in MeOD-d_4

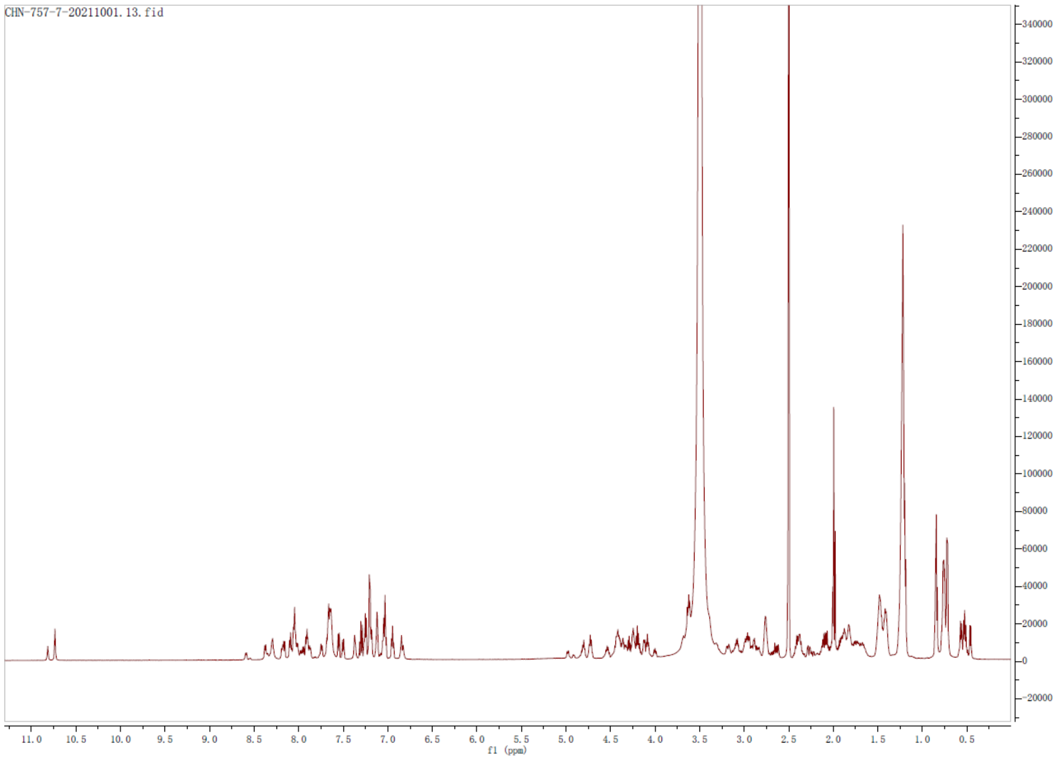

Supplementary Fig. 57. ^{1}H NMR spectrum of glidonin F (**6**) in DMSO-d_6 (600 MHz)

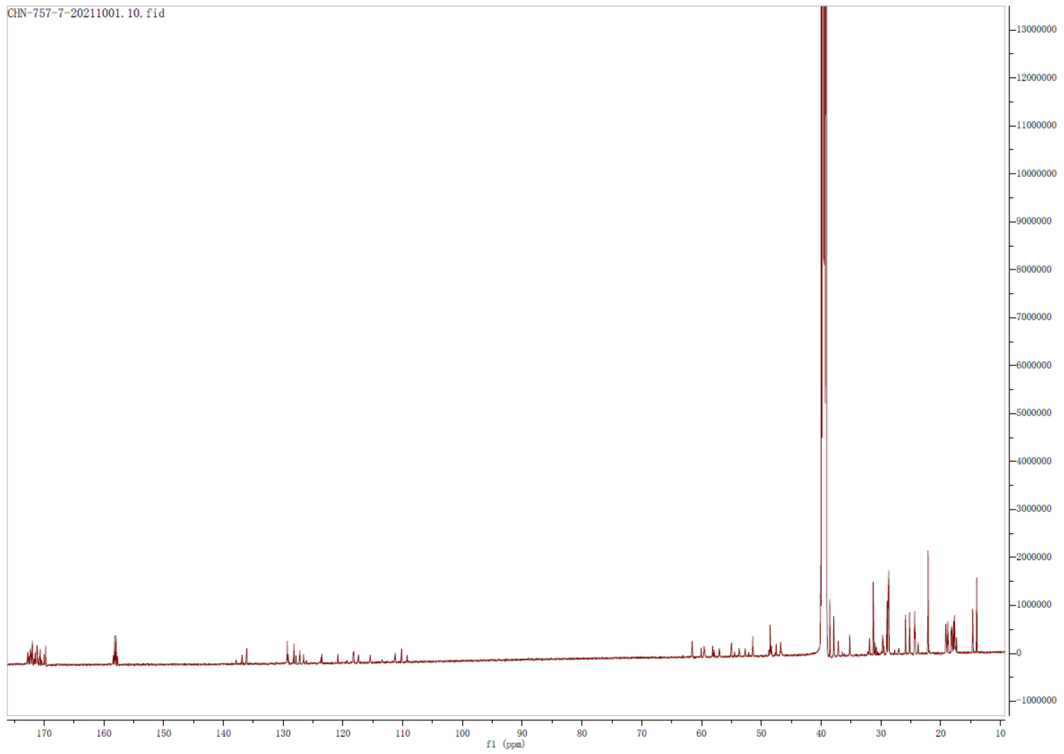

Supplementary Fig. 58. ^{13}C NMR spectrum of glidonin F (**6**) in DMSO-d_6 (150 MHz)

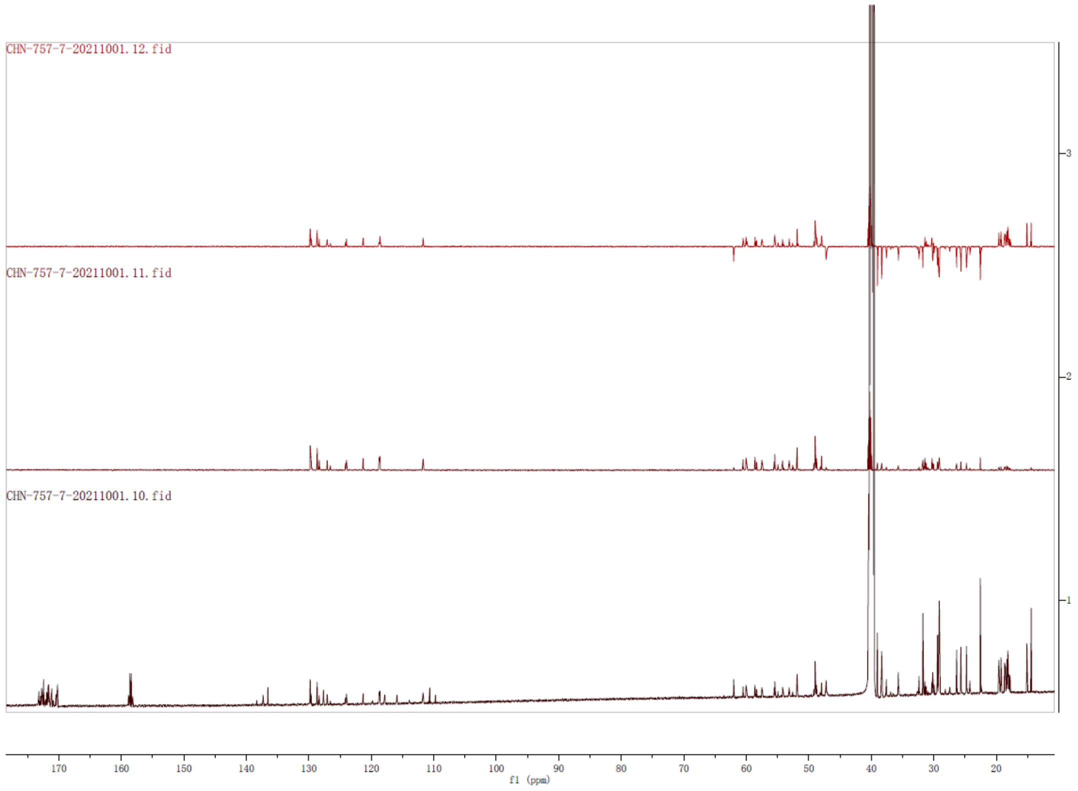

Supplementary Fig. 59. DEPT NMR spectrum of glidonin F (**6**) in DMSO-d_6

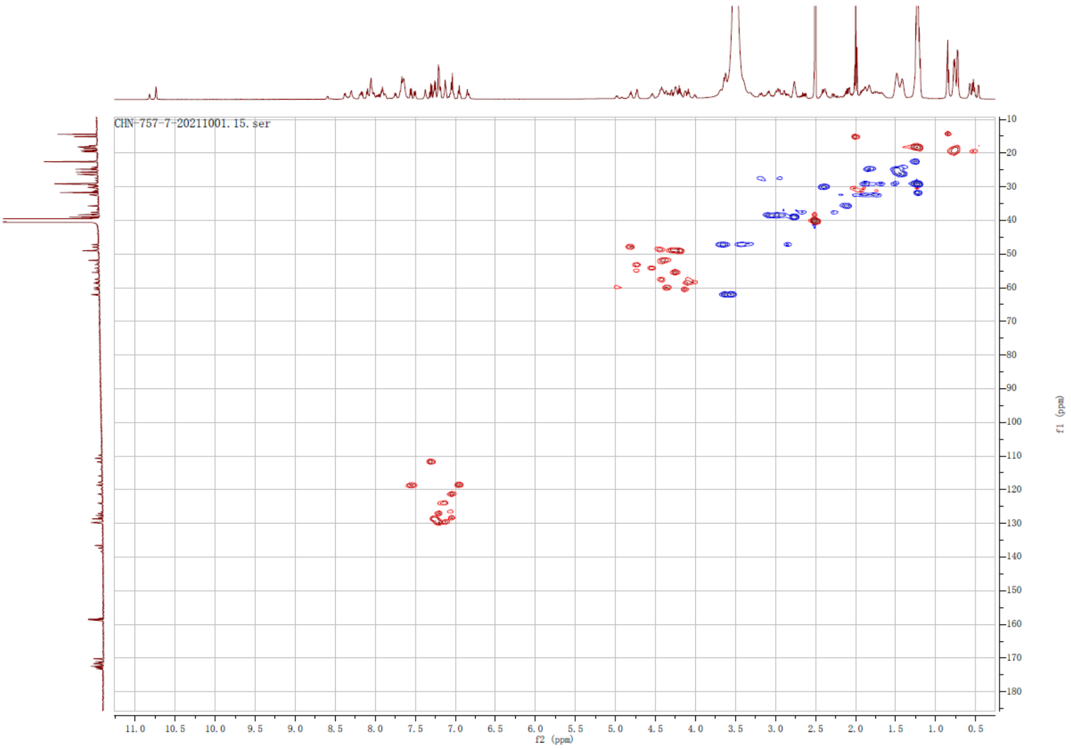

Supplementary Fig. 60. HSQC spectrum of glidonin F (**6**) in DMSO-d_6

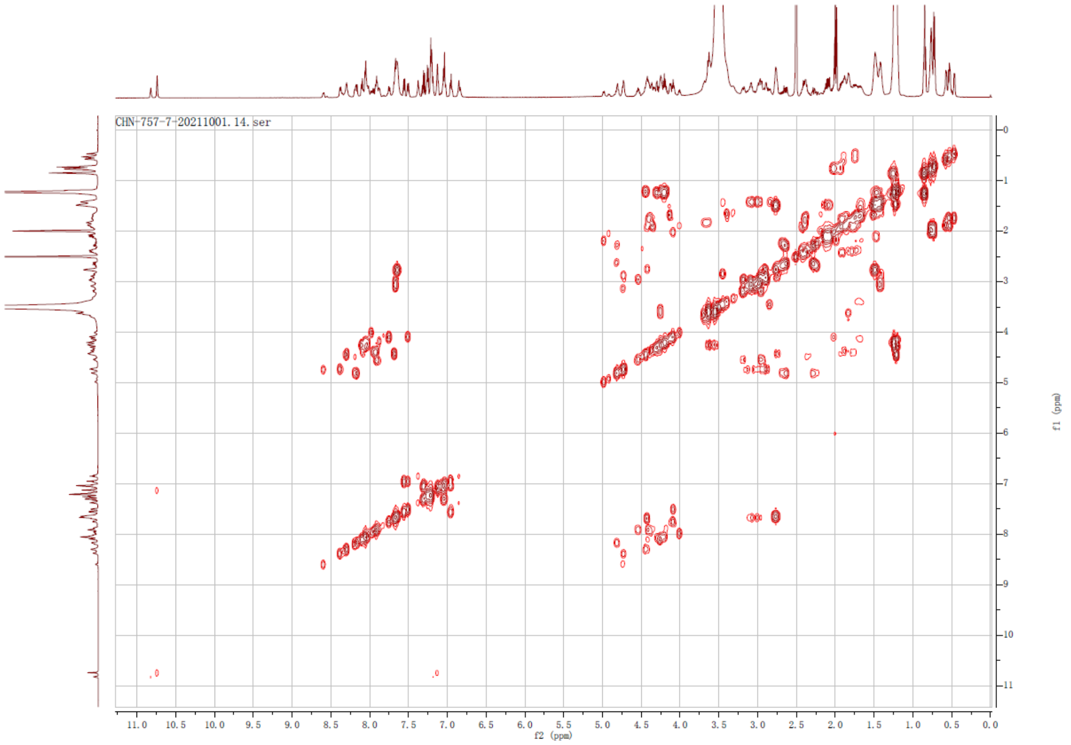

Supplementary Fig. 61. ^{1}H-^{1}H COSY spectrum of glidonin F (**6**) in DMSO-d_6

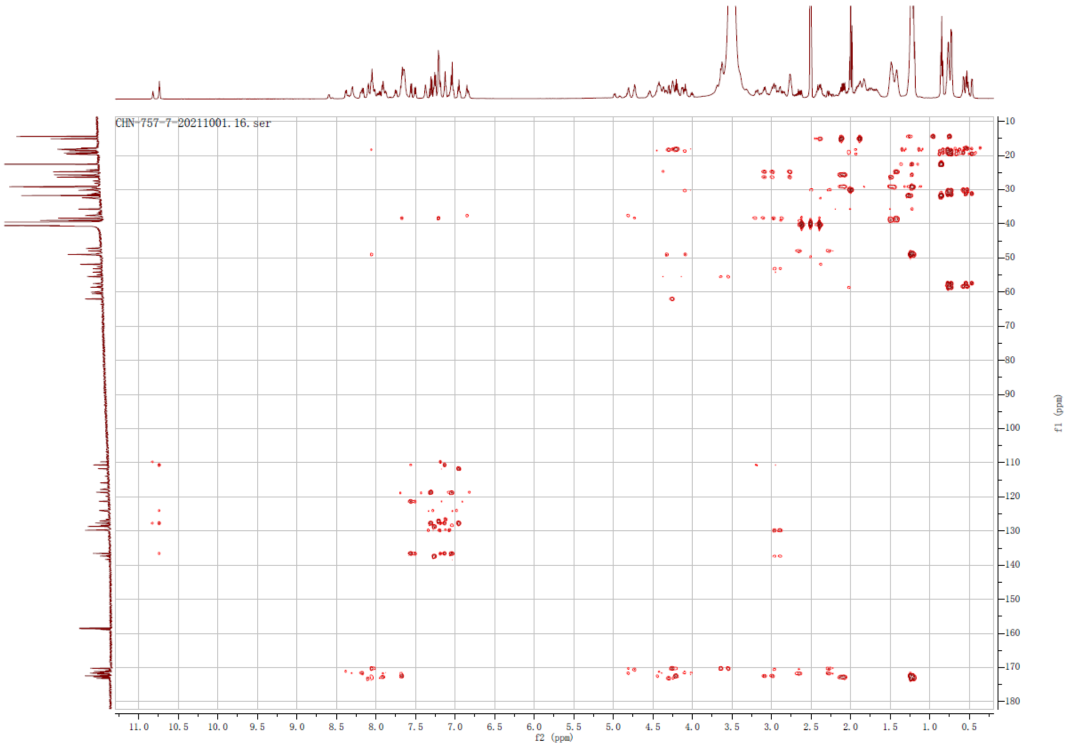

Supplementary Fig. 62. HMBC spectrum of glidonin F (**6**) in DMSO-d_6

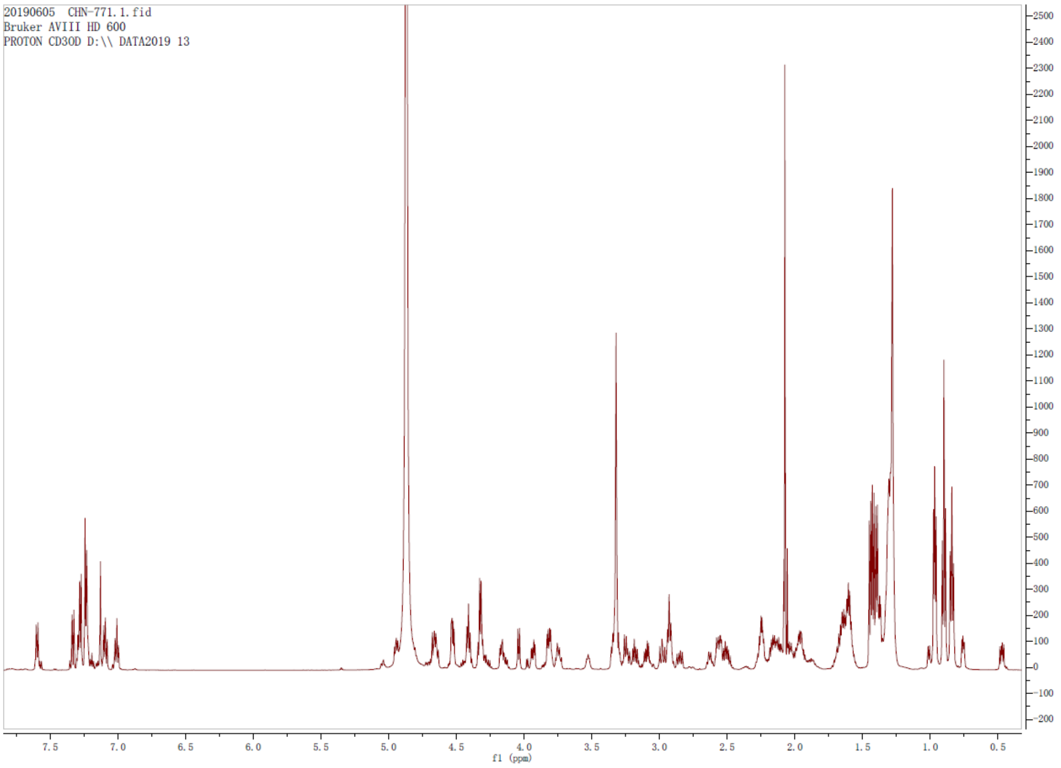

Supplementary Fig. 63. ^{1}H NMR spectrum of glidonin G (**7**) in MeOD-d_4 (600 MHz)

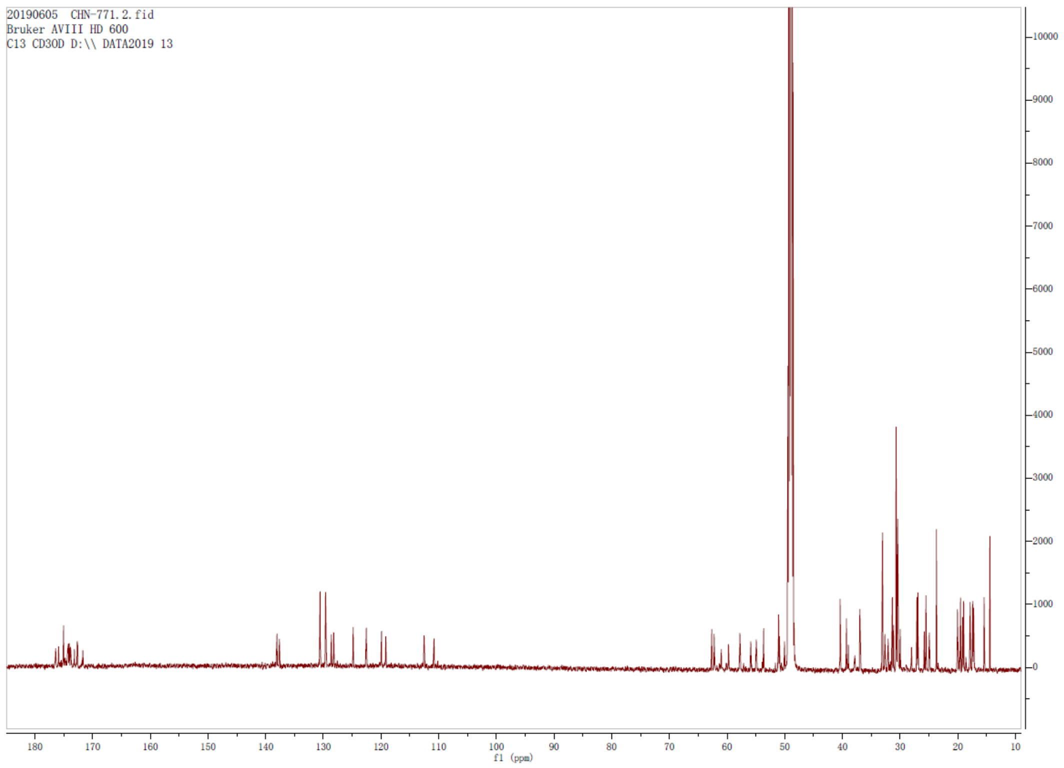

Supplementary Fig. 64. ^{13}C NMR spectrum of glidonin G (**7**) in MeOD-d_4 (150 MHz)

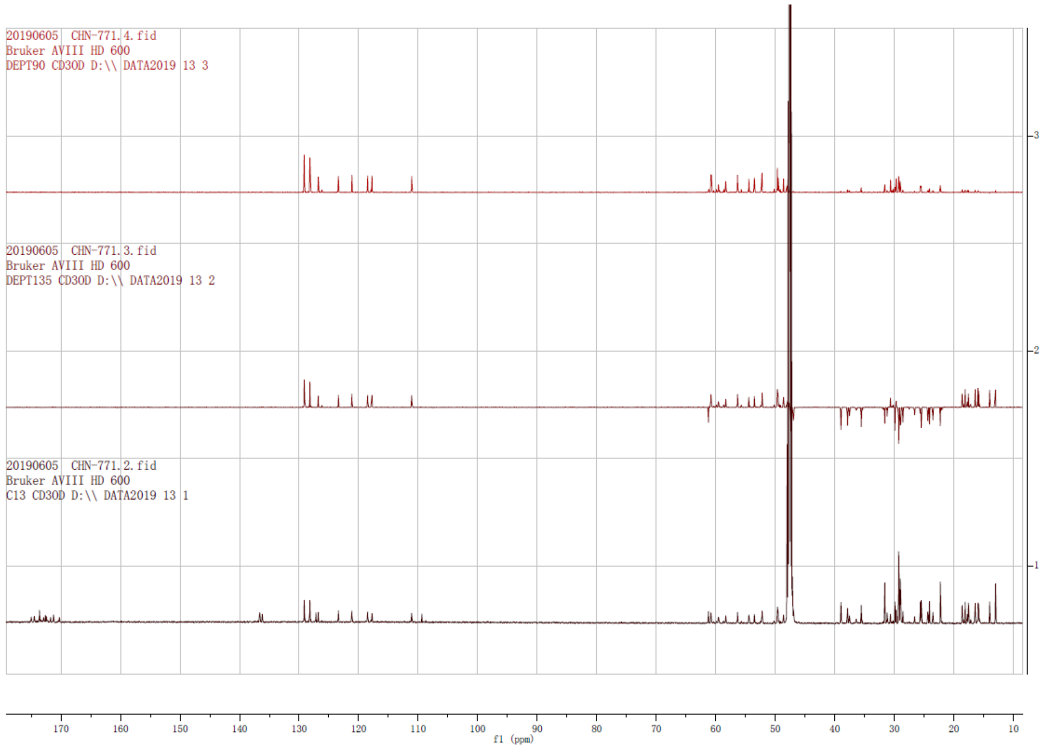

Supplementary Fig. 65. DEPT spectrum of glidonin G (**7**) in MeOD-d_4

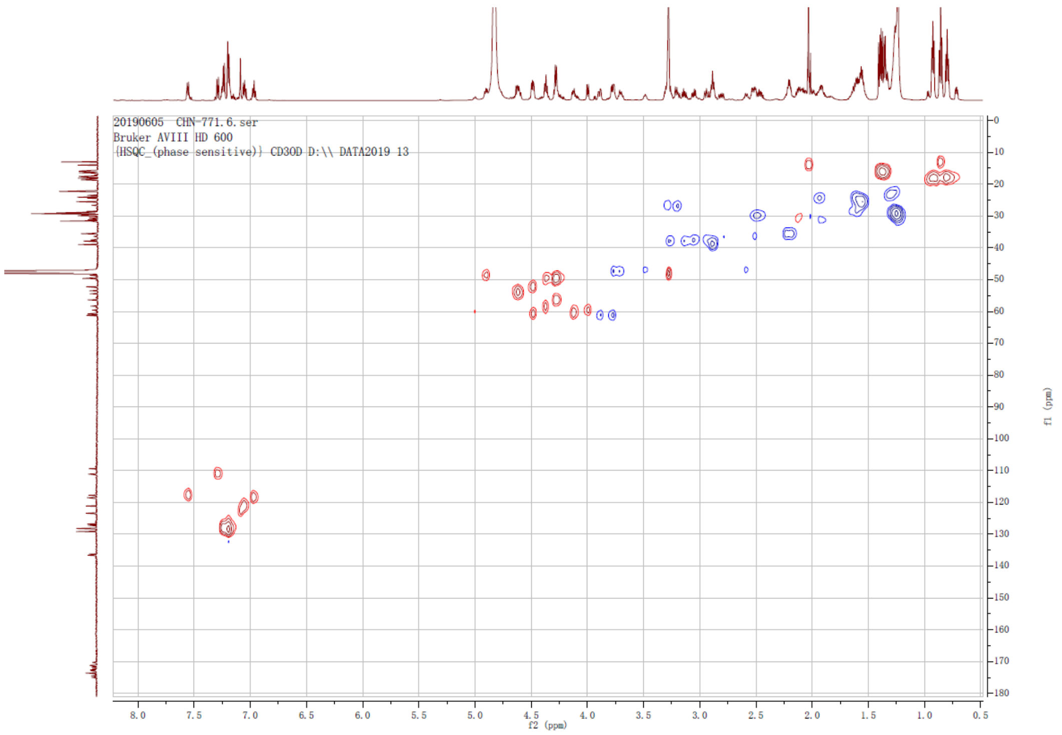

Supplementary Fig. 66. HSQC spectrum of glidonin G (**7**) in MeOD-d_4

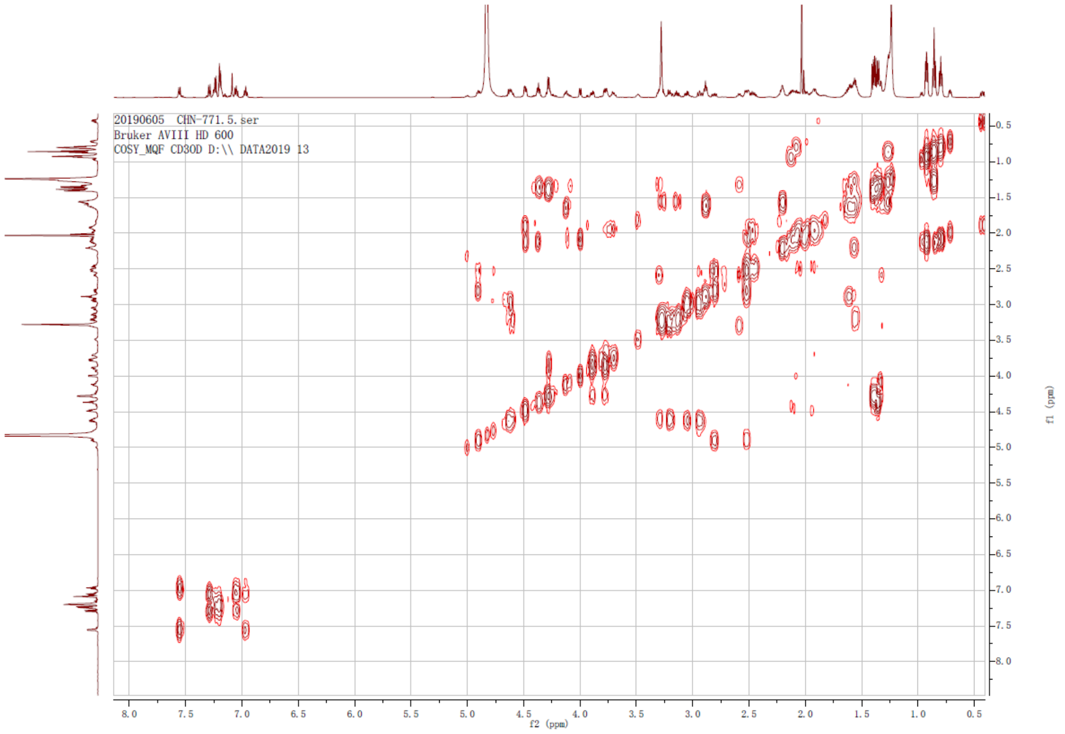

Supplementary Fig. 67. ^{1}H-^{1}H COSY spectrum of glidonin G (**7**) in MeOD-d_4

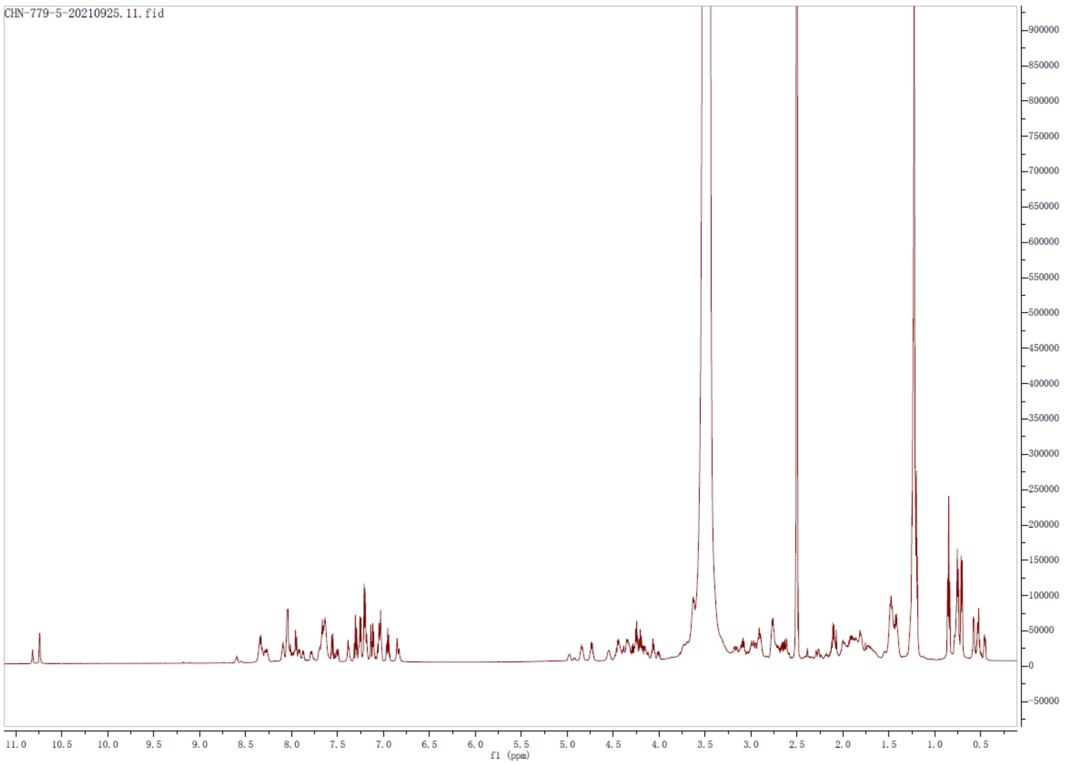

Supplementary Fig. 68. ^{1}H NMR spectrum of glidonin H (**8**) in DMSO-d_6 (600 MHz)

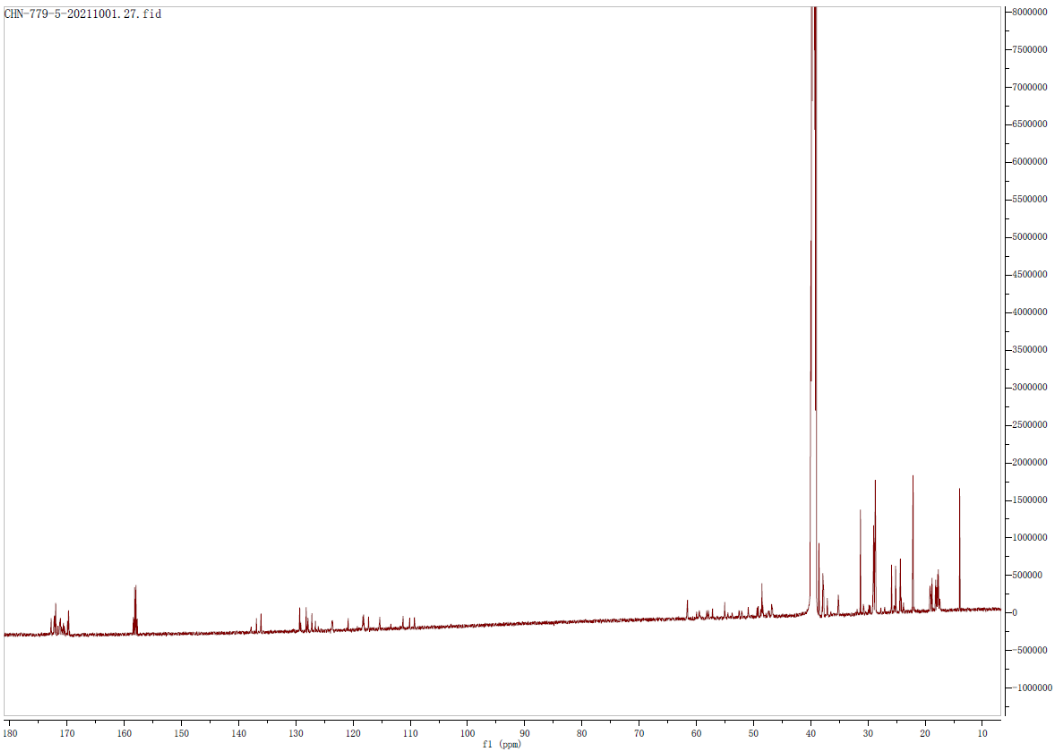

Supplementary Fig. 69. ^{13}C NMR spectrum of glidonin H (**8**) in DMSO-d_6 (150 MHz)

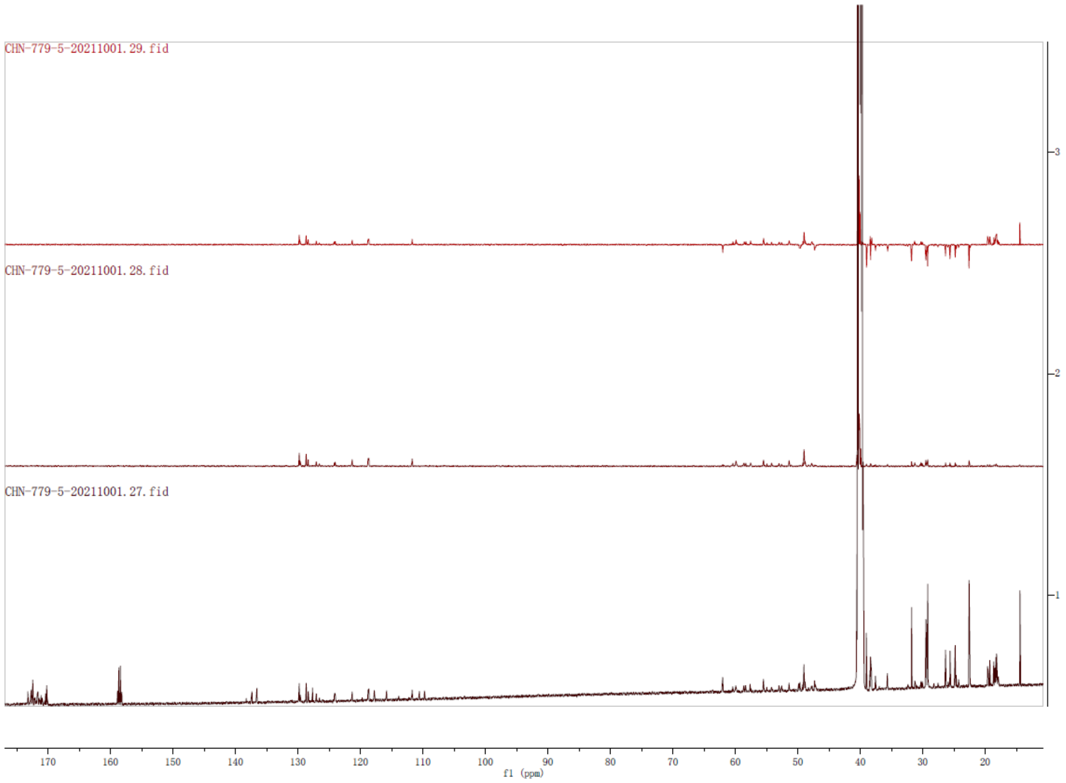

Supplementary Fig. 70. DEPT NMR spectrum of glidonin H (**8**) in DMSO-d_6

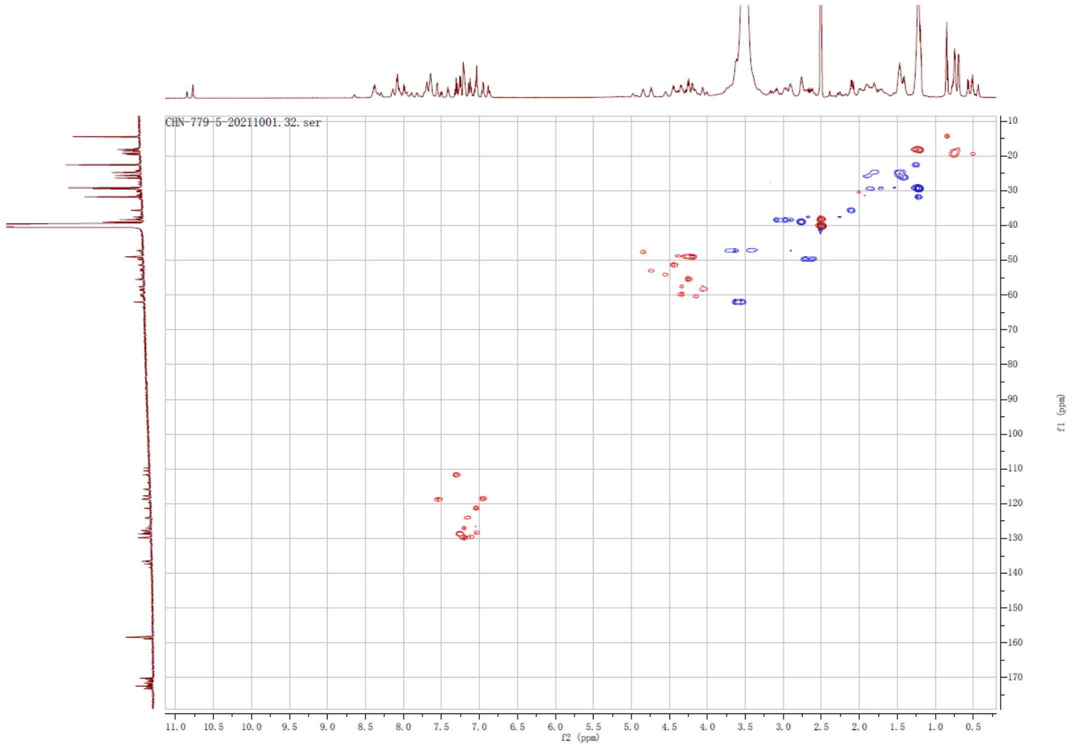

Supplementary Fig. 71. HSQC spectrum of glidonin H (**8**) in DMSO-d_6

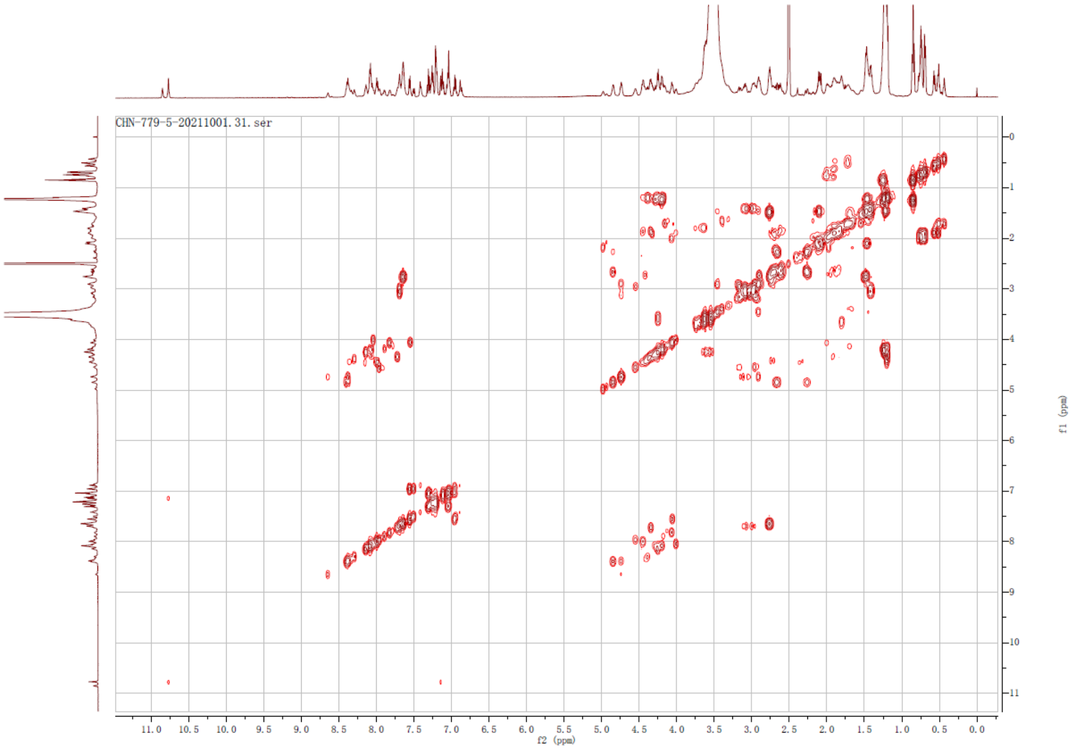

Supplementary Fig. 72. ^{1}H-^{1}H COSY spectrum of glidonin H (**8**) in DMSO-d_6

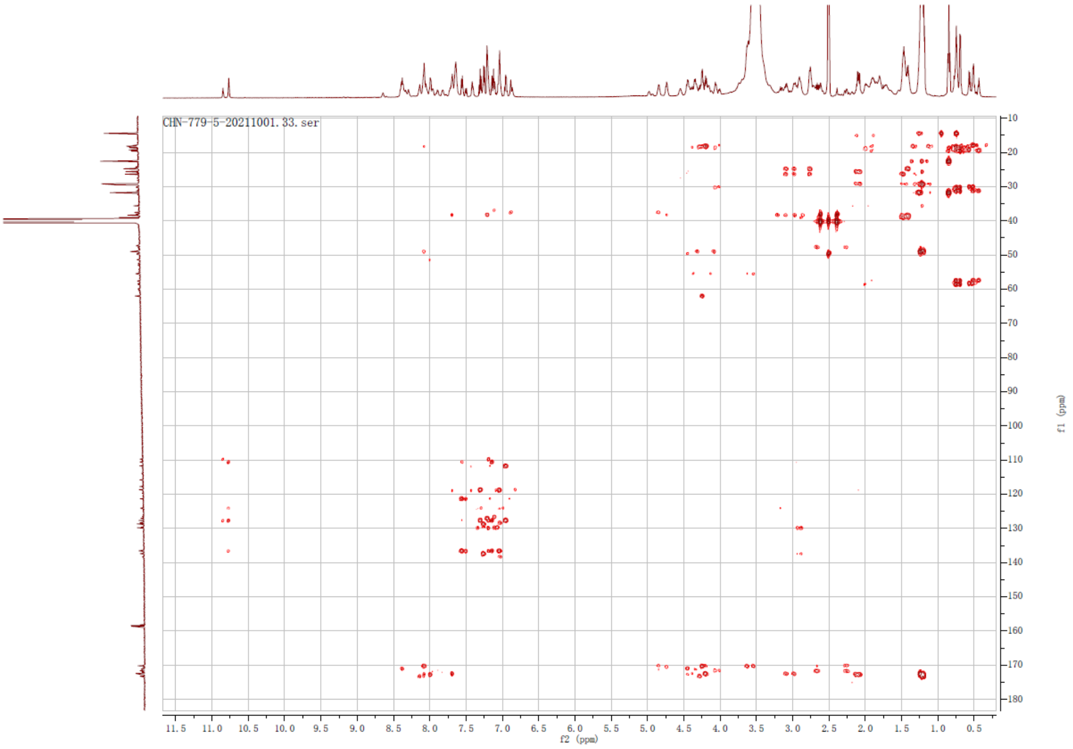

Supplementary Fig. 73. HMBC spectrum of glidonin H (**8**) in DMSO-d_6

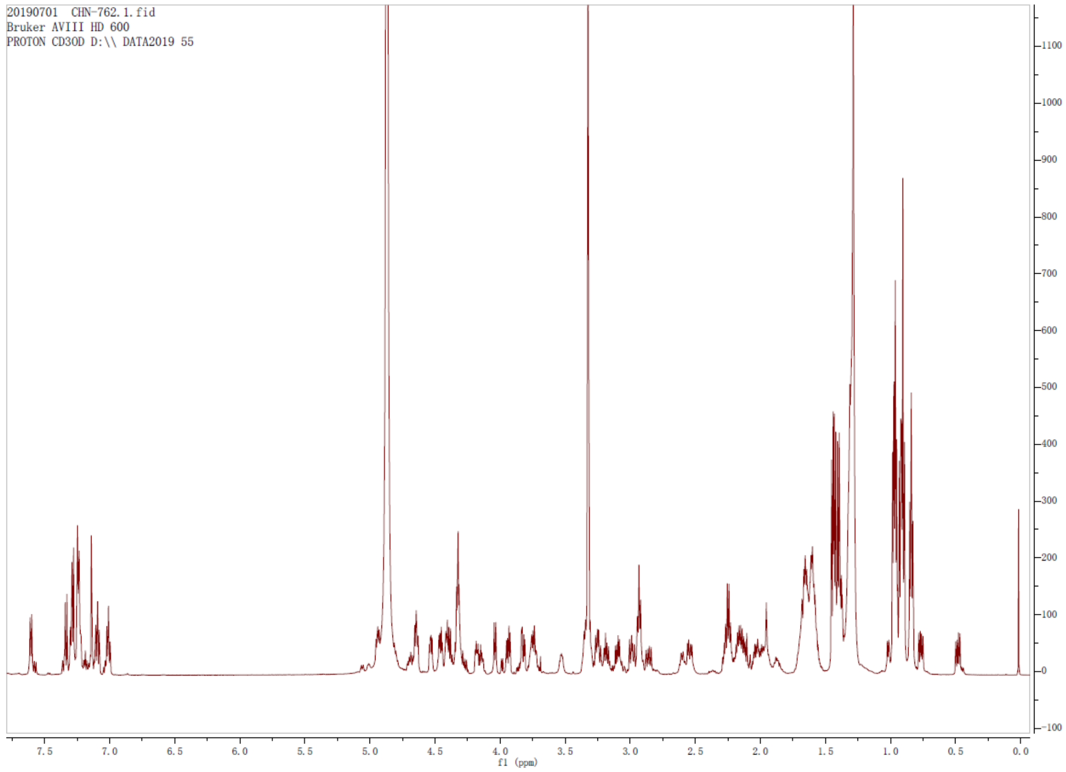

Supplementary Fig. 74. ^{1}H NMR spectrum of glidonin I (**9**) in MeOD-d_4 (600 MHz)

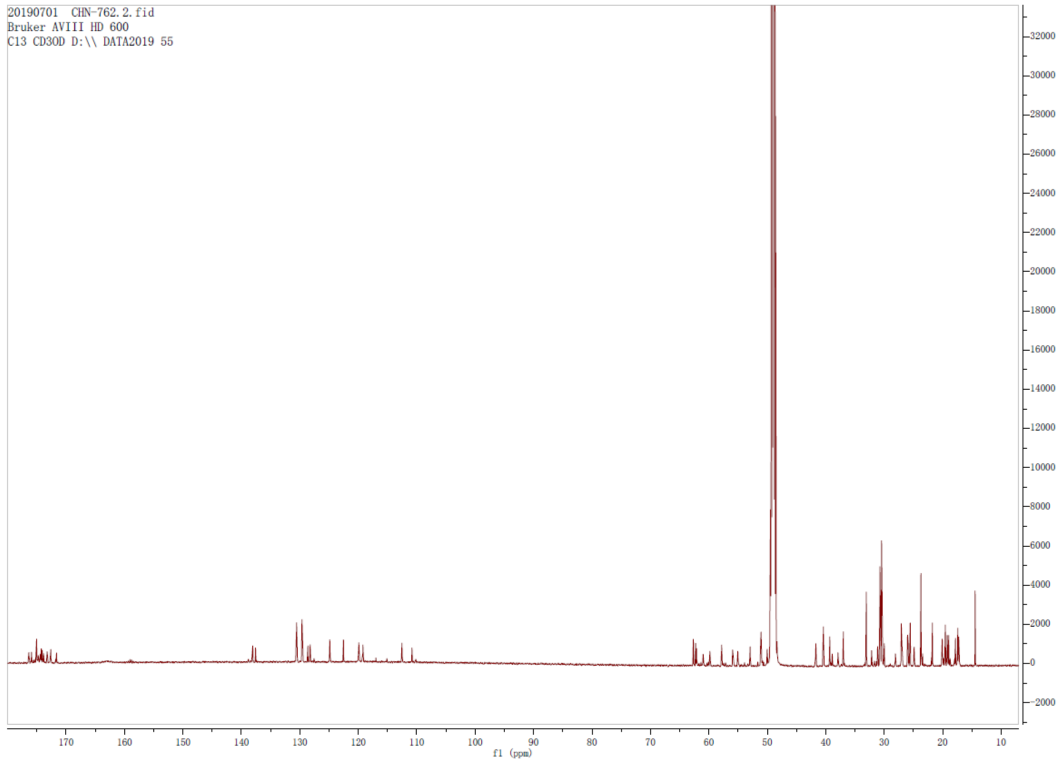

Supplementary Fig. 75. ^{13}C NMR spectrum of glidonin I (**9**) in MeOD-d_4 (150 MHz)

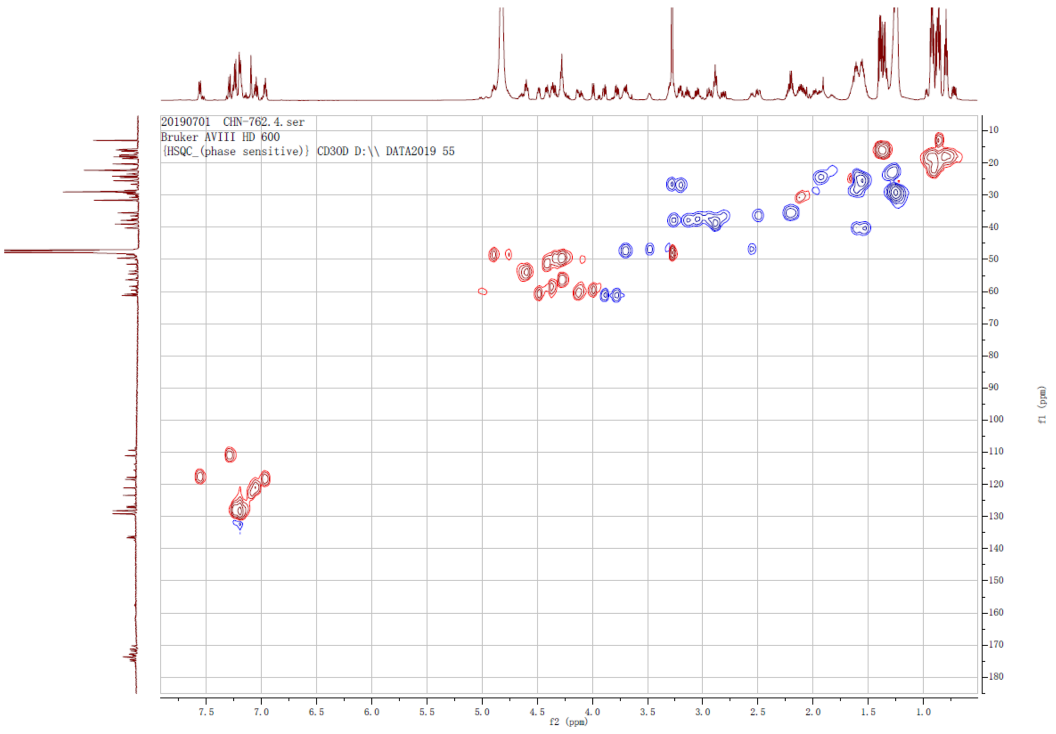

Supplementary Fig. 76. HSQC spectrum of glidonin I (**9**) in MeOD-d_4

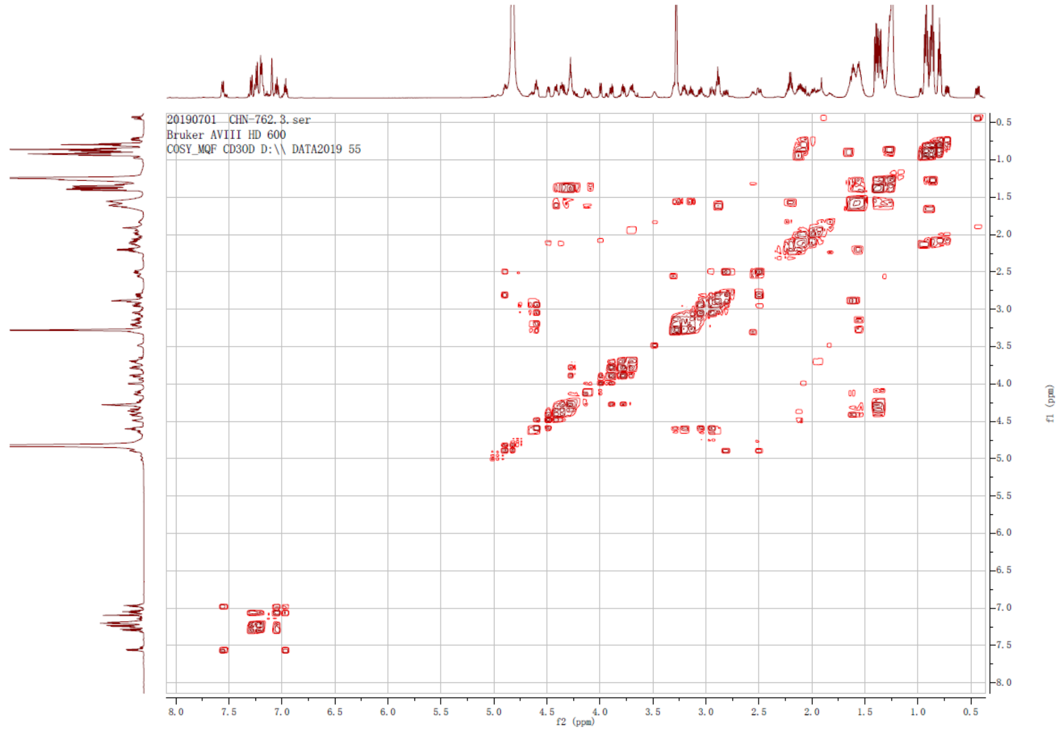

Supplementary Fig. 77. ^{1}H-^{1}H COSY spectrum of glidonin I (**9**) in MeOD-d_4

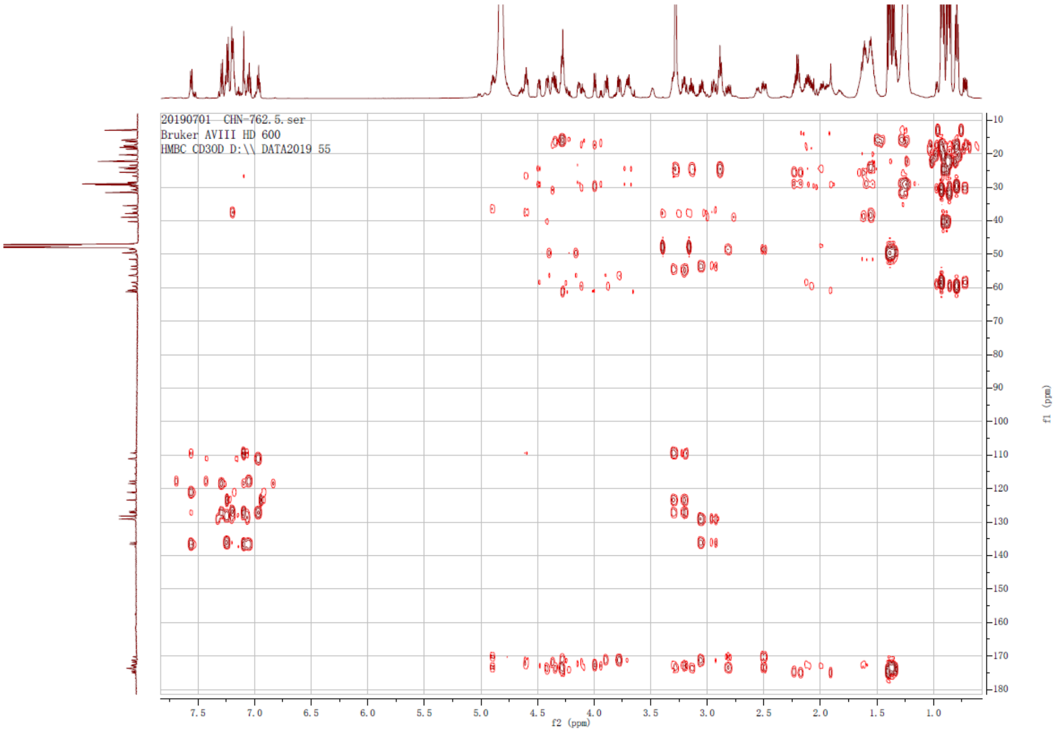

Supplementary Fig. 78. HMBC spectrum of glidonin I (**9**) in MeOD-d_4

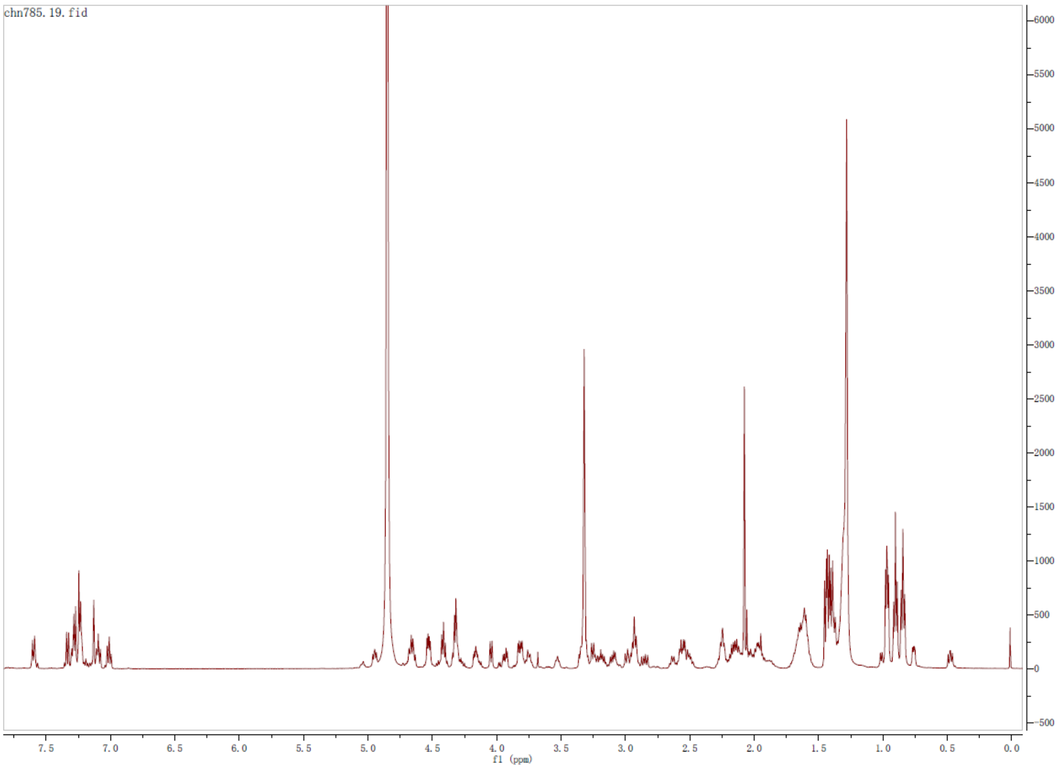

Supplementary Fig. 79. ^{1}H NMR spectrum of glidonin J (**10**) in MeOD-d_4 (600 MHz)

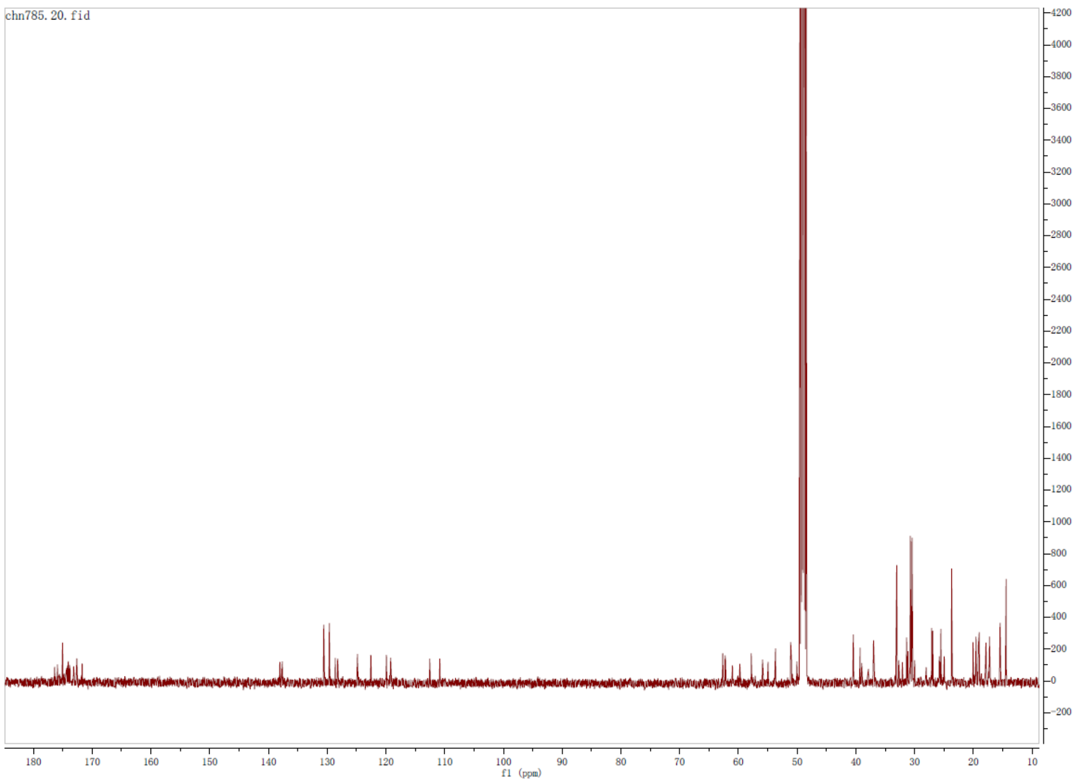

Supplementary Fig. 80. ^{13}C NMR spectrum of glidonin J (**10**) in MeOD-d_4 (150 MHz)

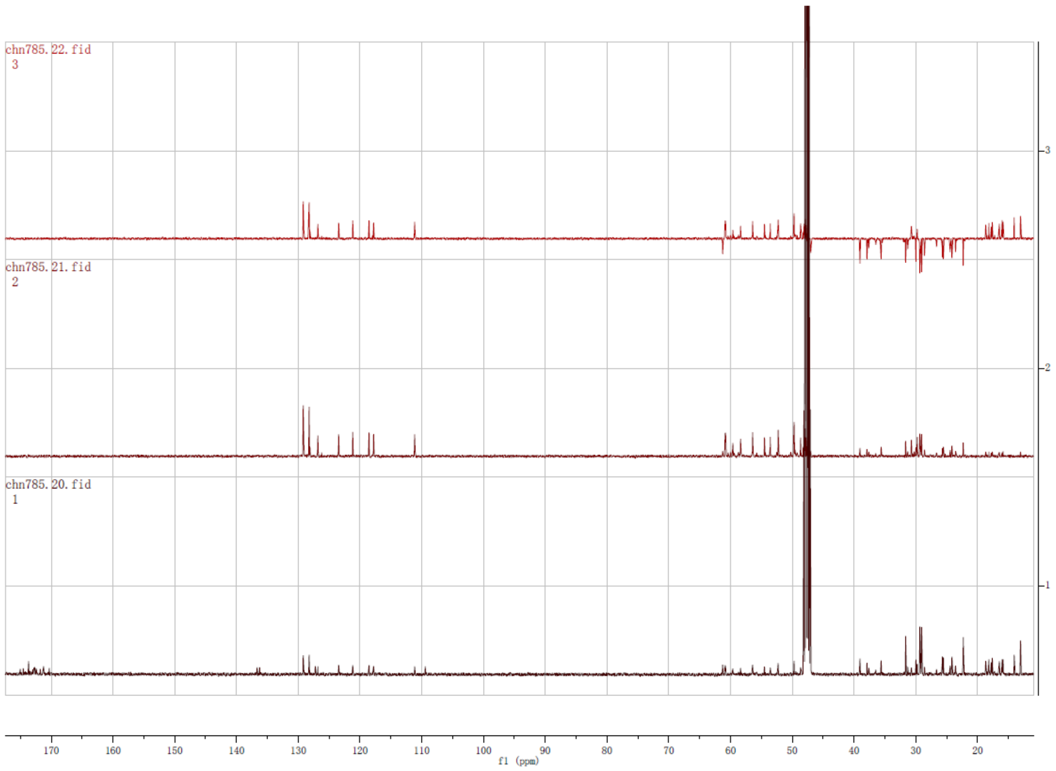

Supplementary Fig. 81. DEPT NMR spectrum of glidonin J (**10**) in MeOD-d_4

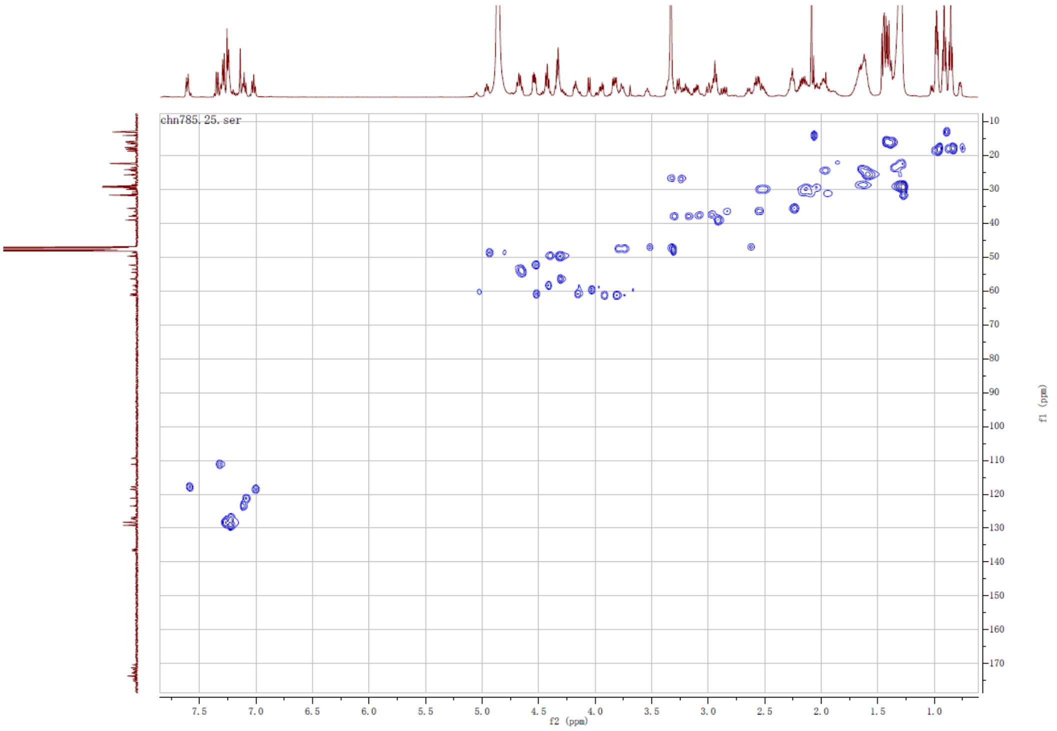

Supplementary Fig. 82. HSQC spectrum of glidonin J (**10**) in MeOD-*d*4

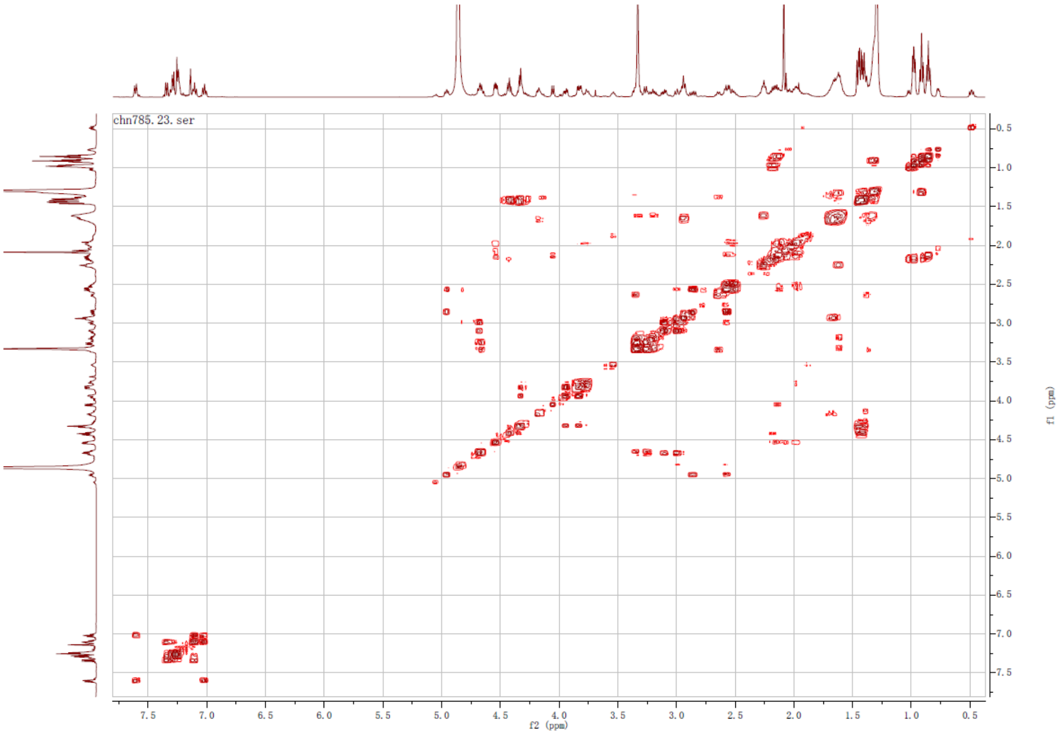

Supplementary Fig. 83. ^{1}H-^{1}H COSY spectrum of glidonin J (**10**) in MeOD-*d*4

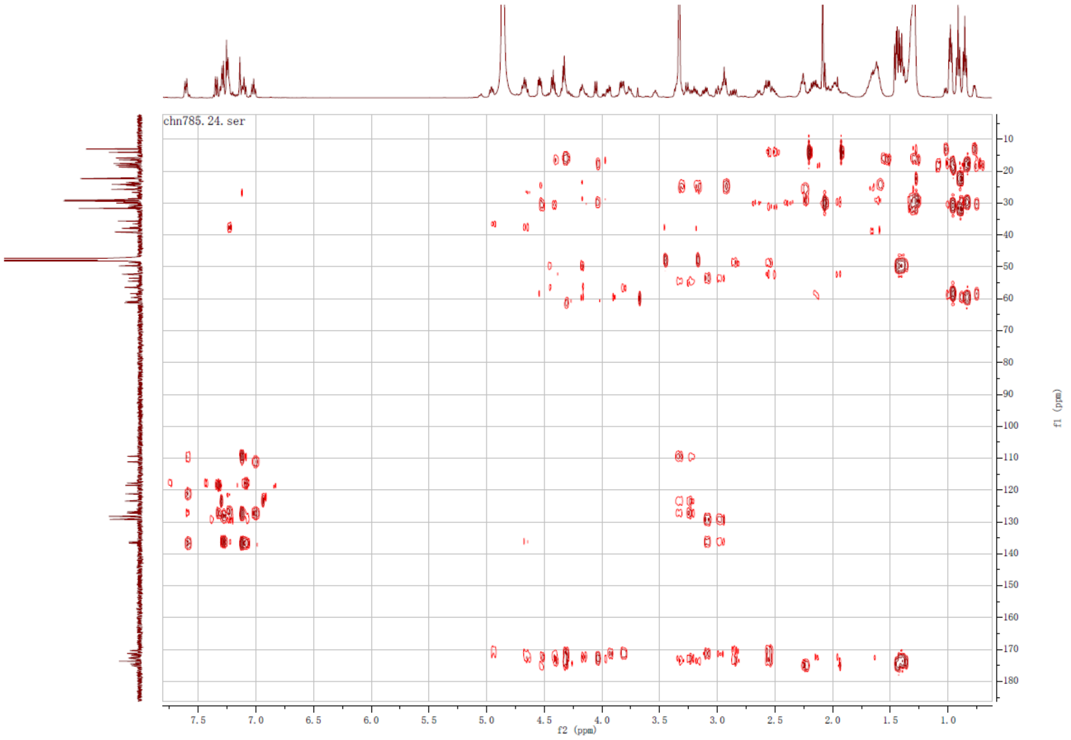

Supplementary Fig. 84. HMBC spectrum of glidonin J (**10**) in MeOD-d_4

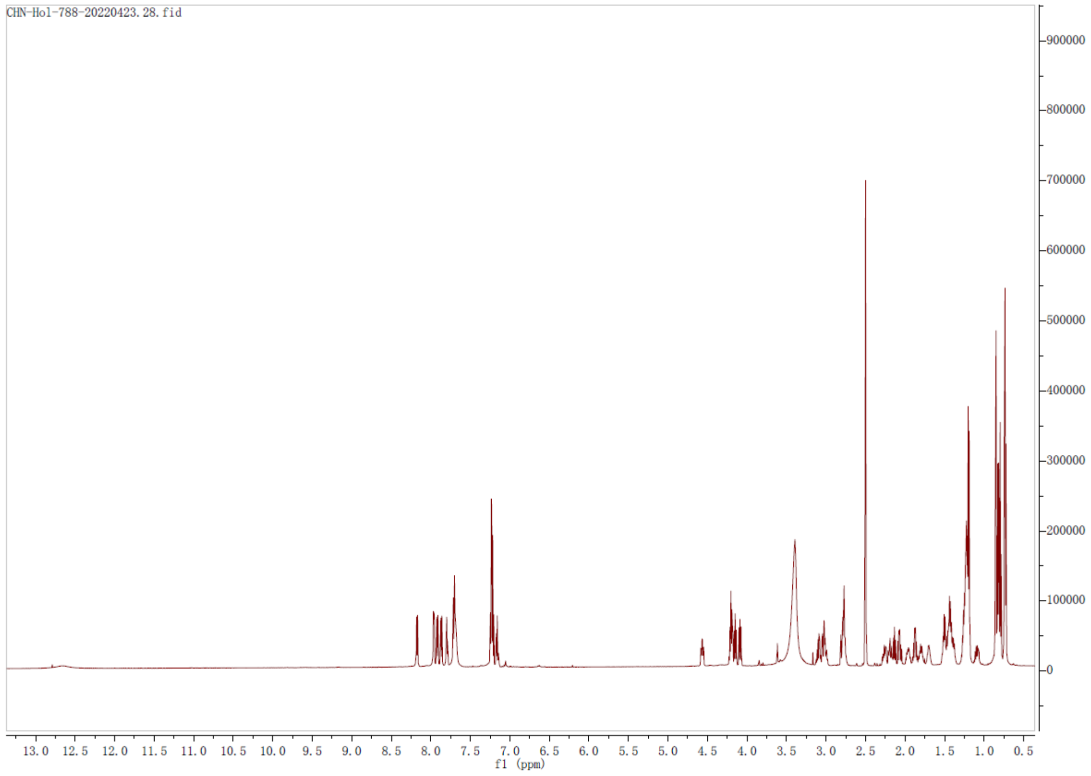

Supplementary Fig. 85. ^{1}H NMR spectrum of **31** in DMSO-d_6 (600 MHz)

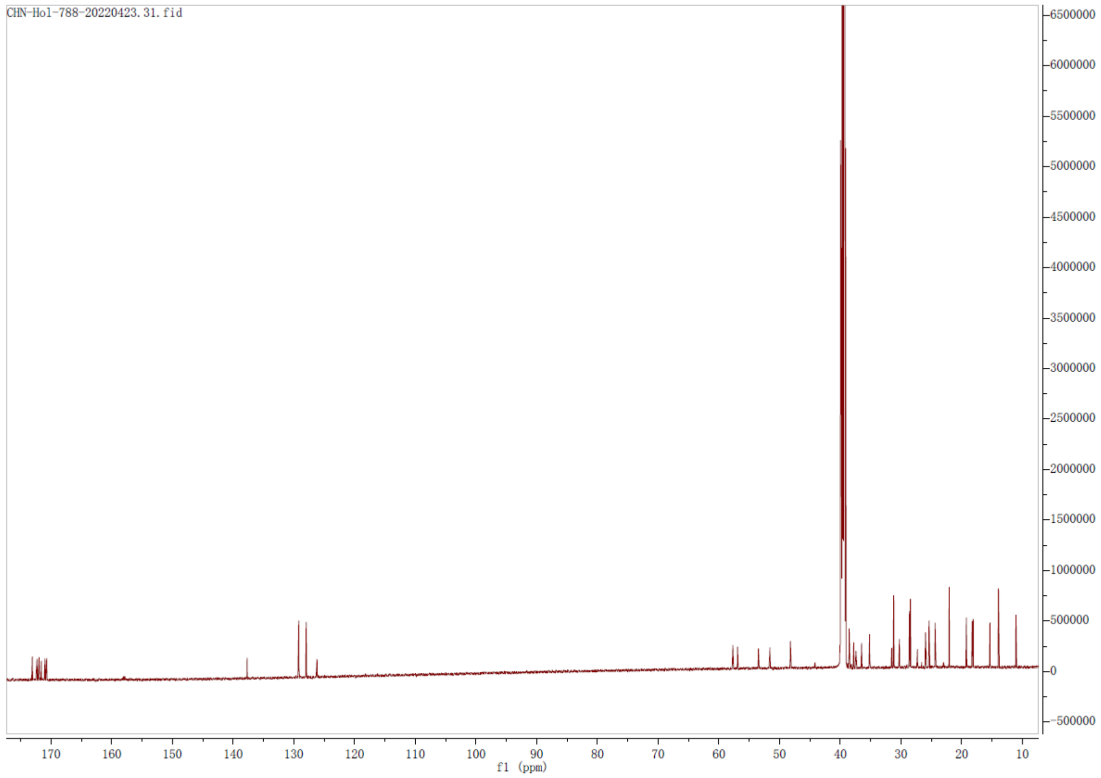

Supplementary Fig. 86. ^{13}C NMR spectrum of **31** in DMSO-d_6 (150 MHz)

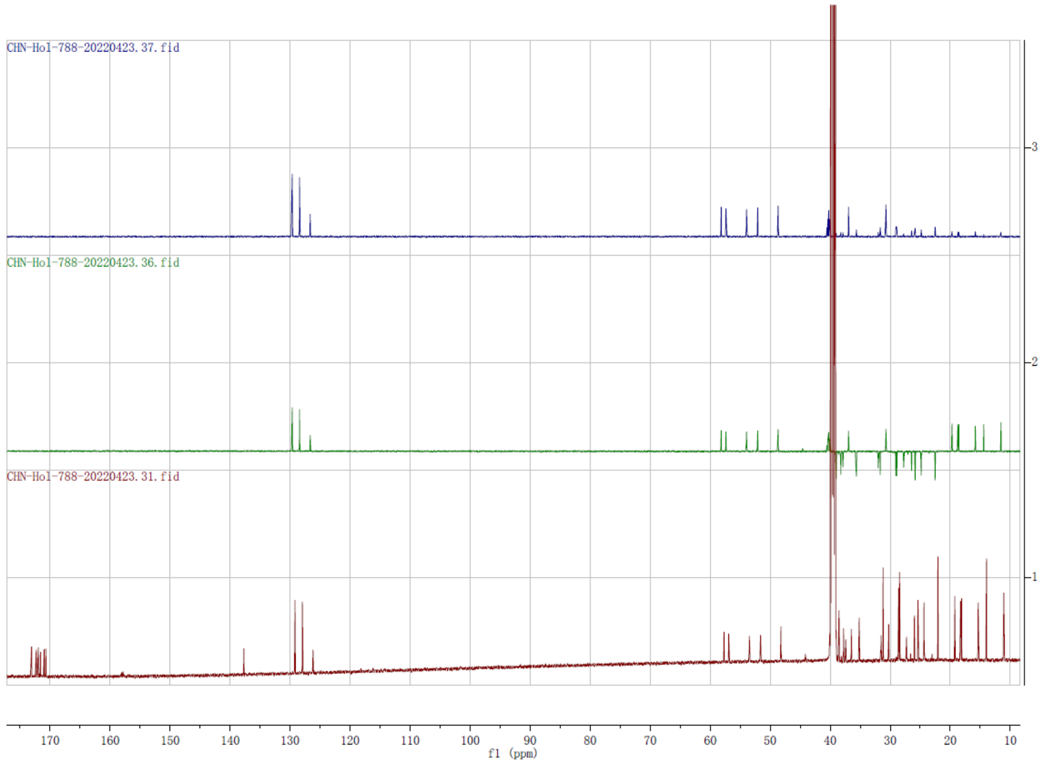

Supplementary Fig. 87. DEPT spectrum of **31** in DMSO-d_6

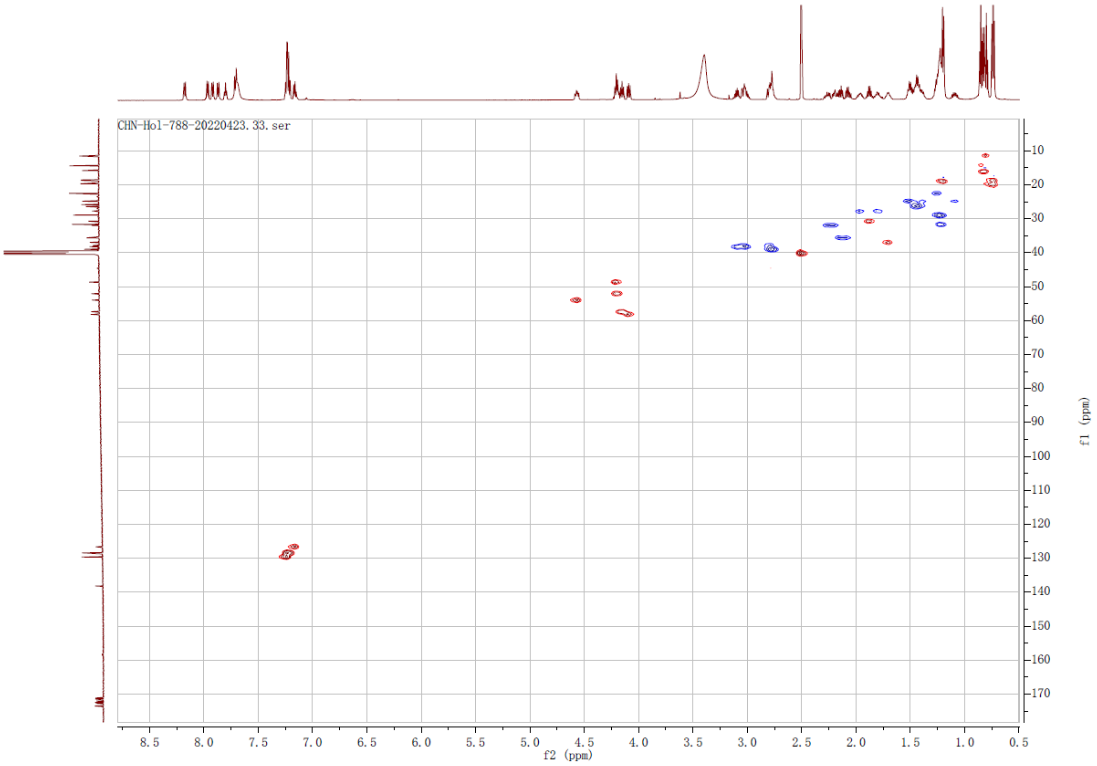

Supplementary Fig. 88. HSQC spectrum of **31** in DMSO-d_6

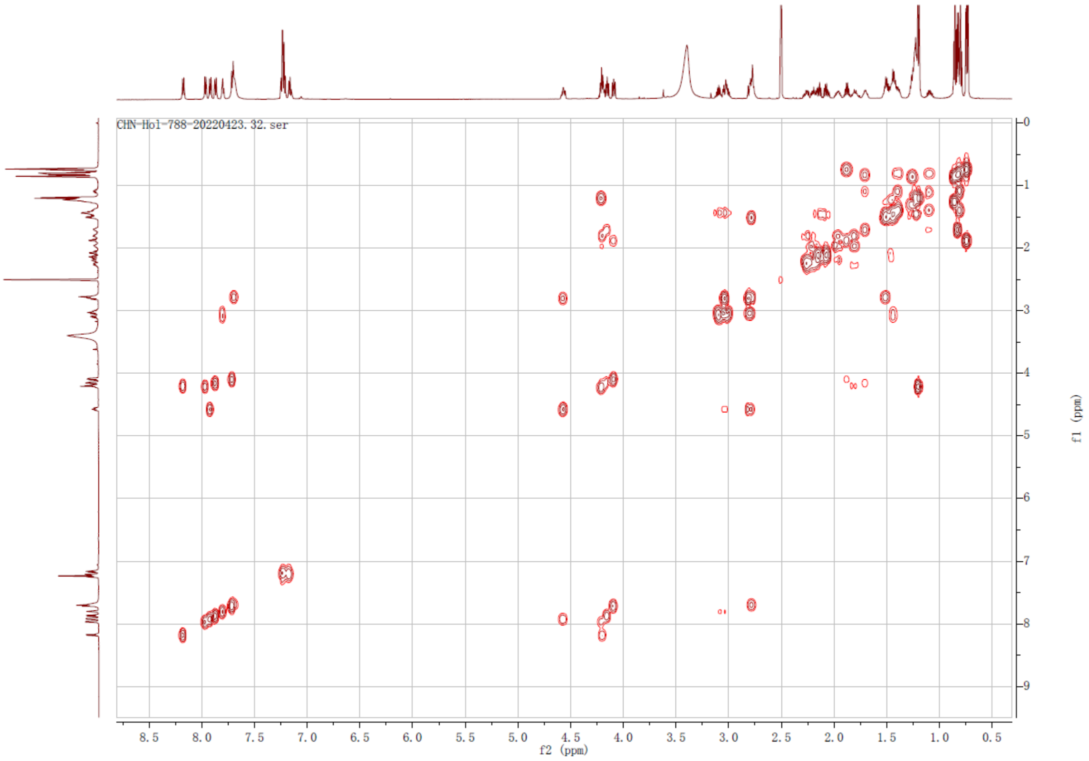

Supplementary Fig. 89. ^{1}H-^{1}H COSY spectrum of **31** in DMSO-d_6

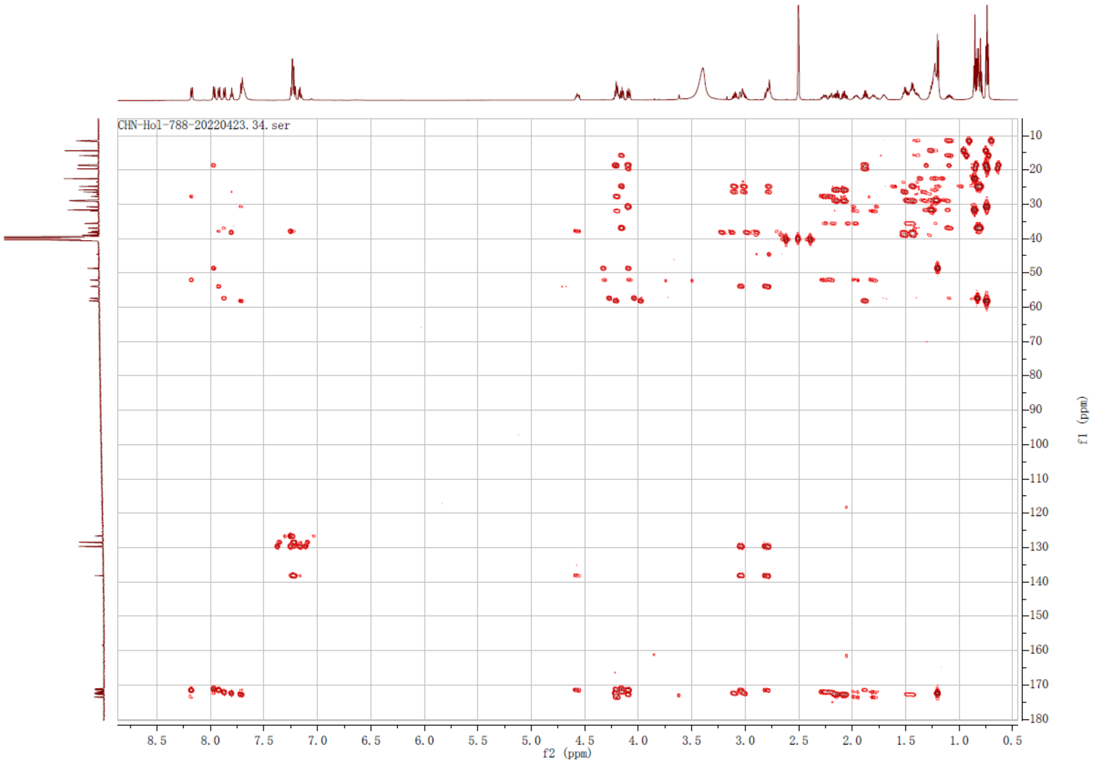

Supplementary Fig. 90. HMBC spectrum of **31** in DMSO-d_6

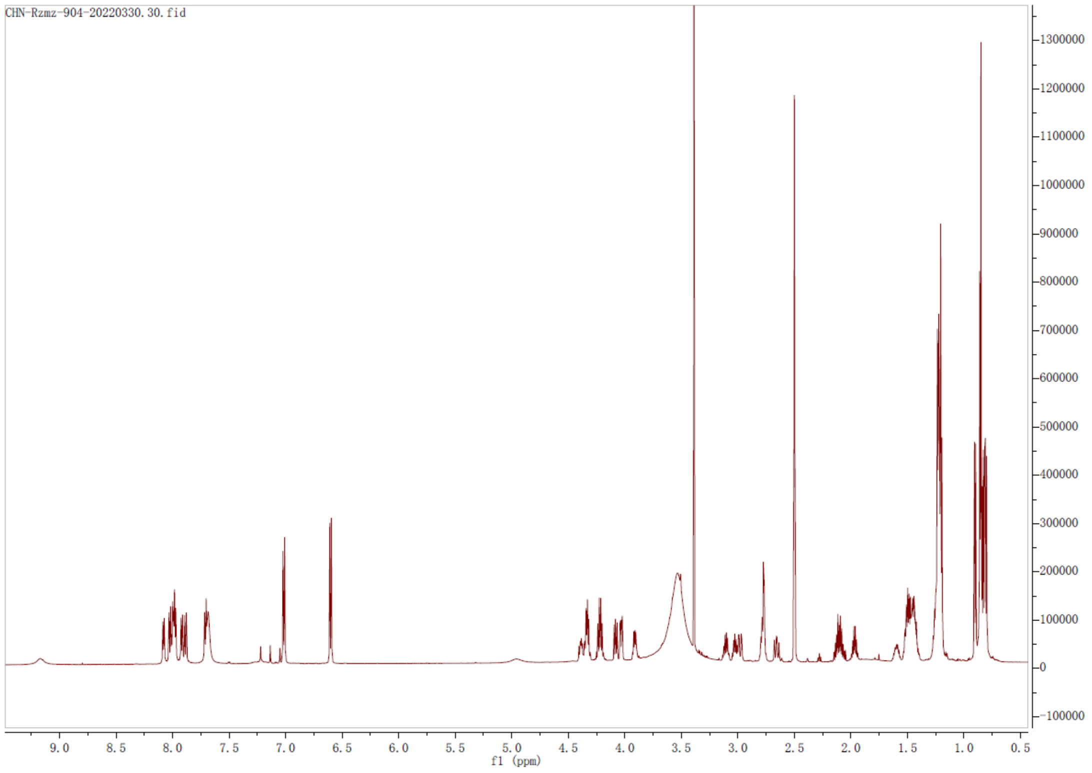

Supplementary Fig. 91. ^{1}H NMR spectrum of **29** in DMSO-d_6 (600 MHz)

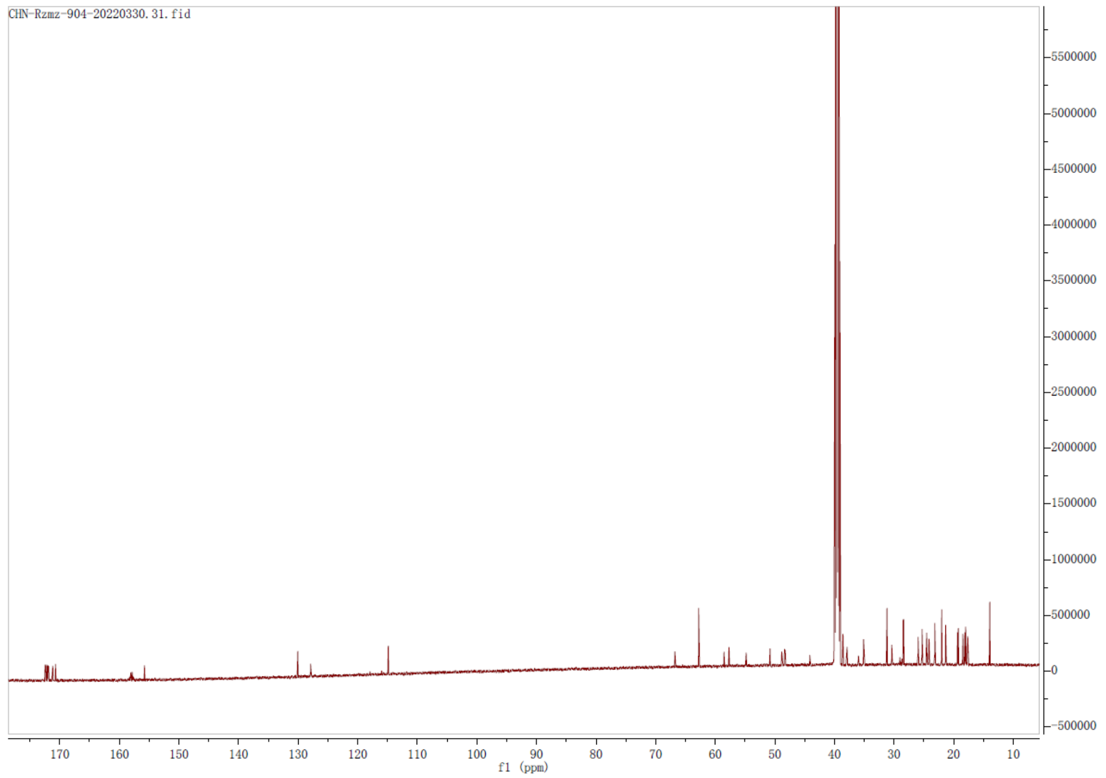

Supplementary Fig. 92. ^{13}C NMR spectrum of **29** in DMSO-d_6 (150 MHz)

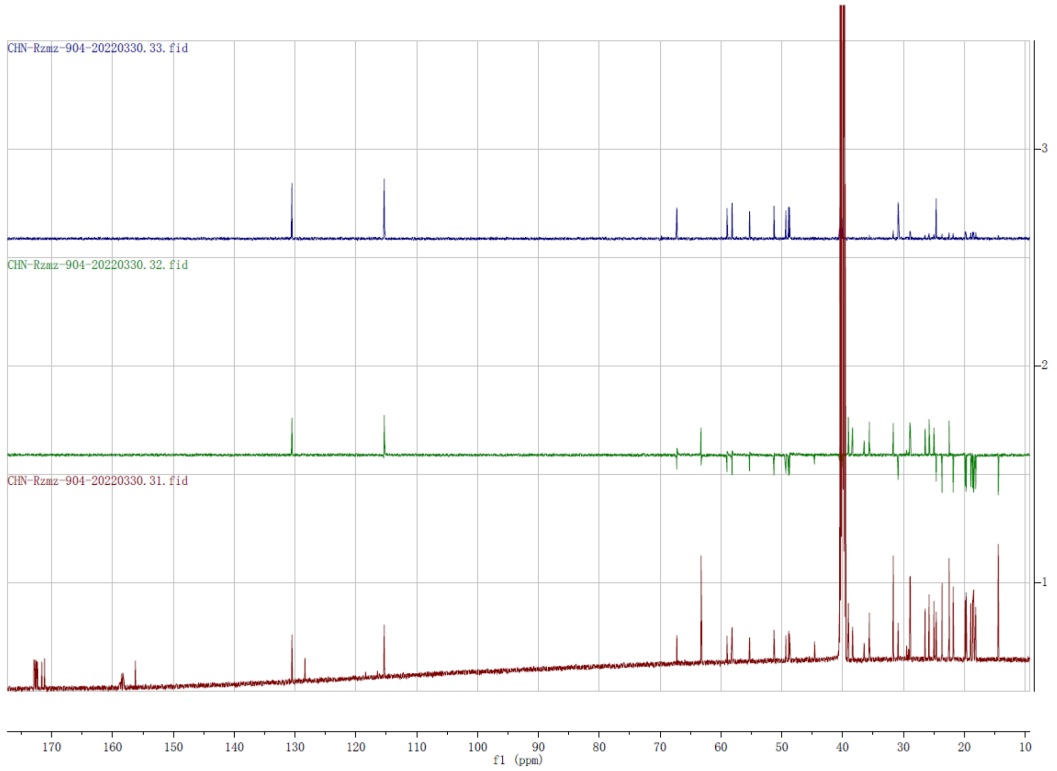

Supplementary Fig. 93. DEPT spectrum of **29** in DMSO-d_6

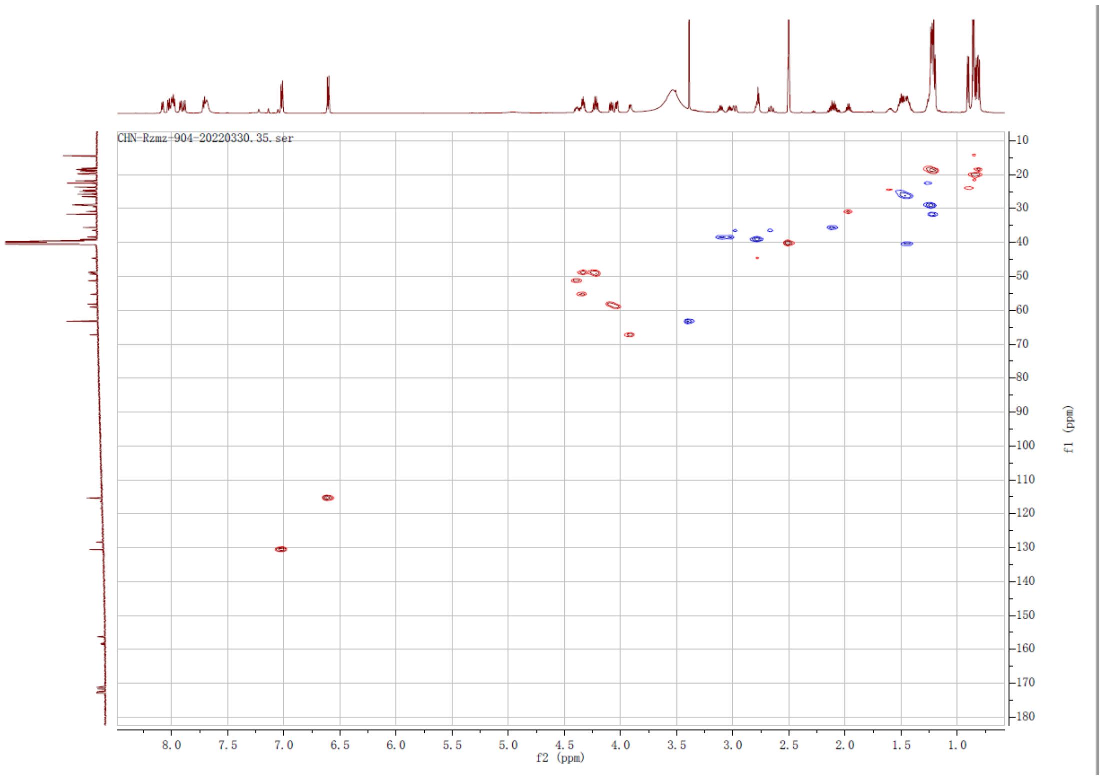

Supplementary Fig. 94. HSQC spectrum of **29** in DMSO-d_6

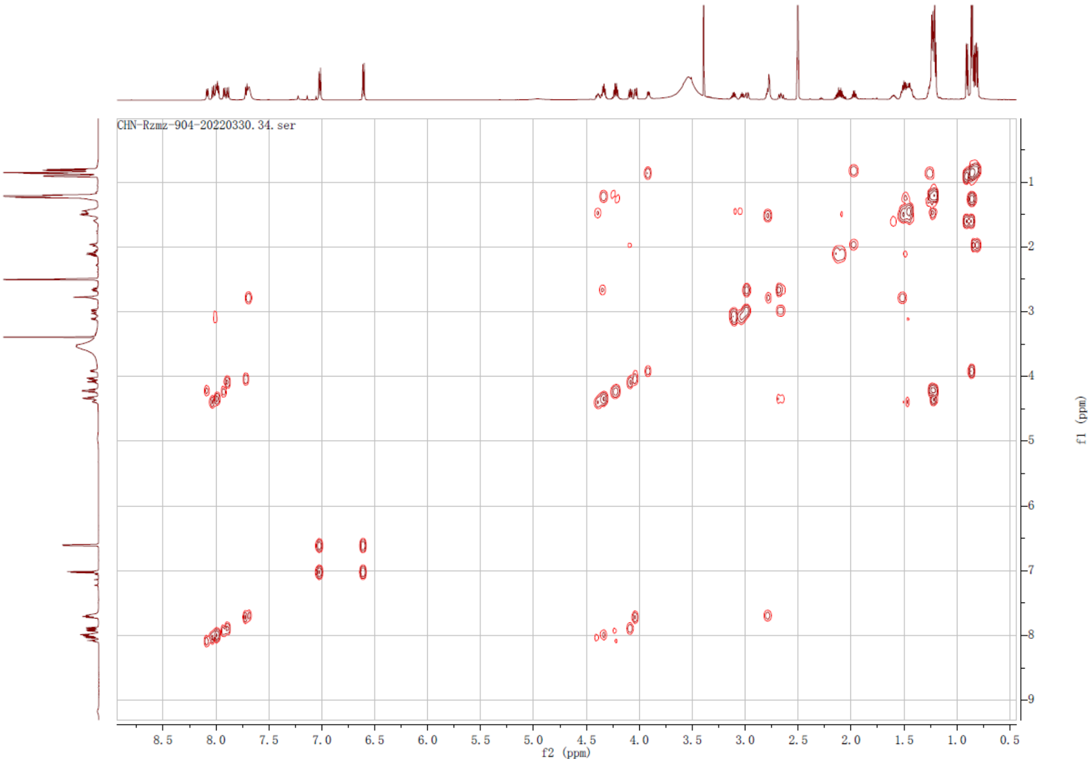

Supplementary Fig. 95. ^{1}H-^{1}H COSY spectrum of **29** in DMSO-d_6

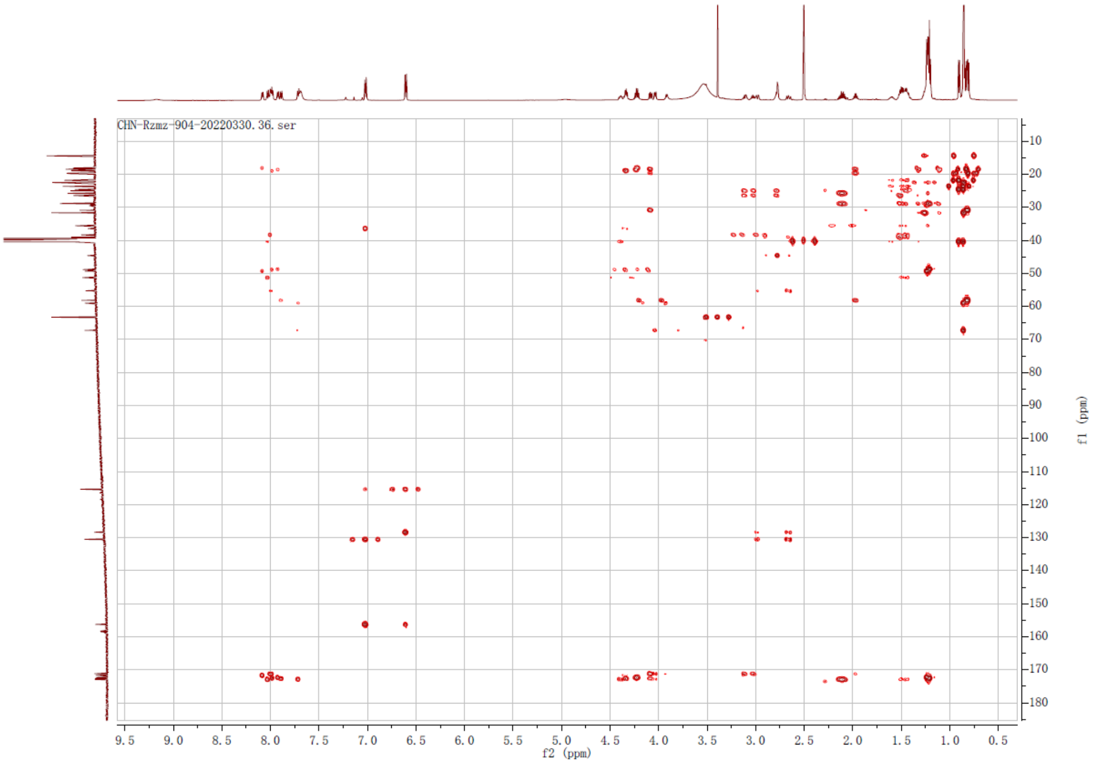

Supplementary Fig. 96. HMBC spectrum of **29** in DMSO-d_6

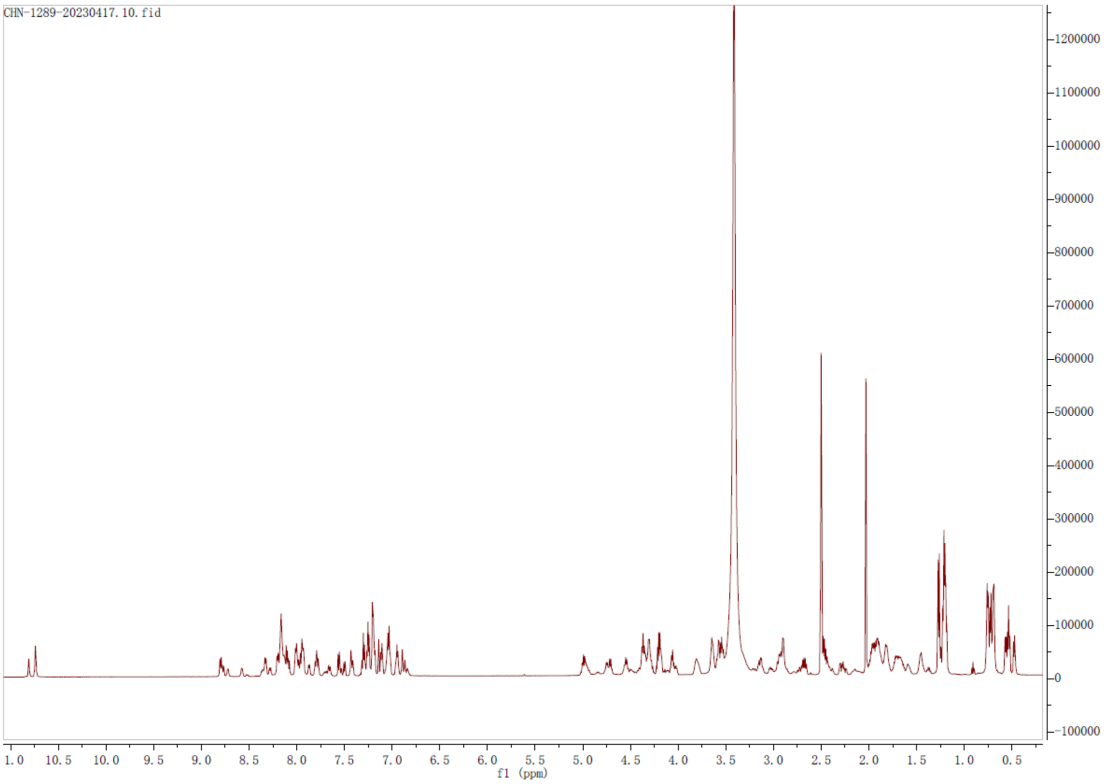

Supplementary Fig. 97. ^{1}H NMR spectrum of **1a** in DMSO-d_6 (600 MHz)

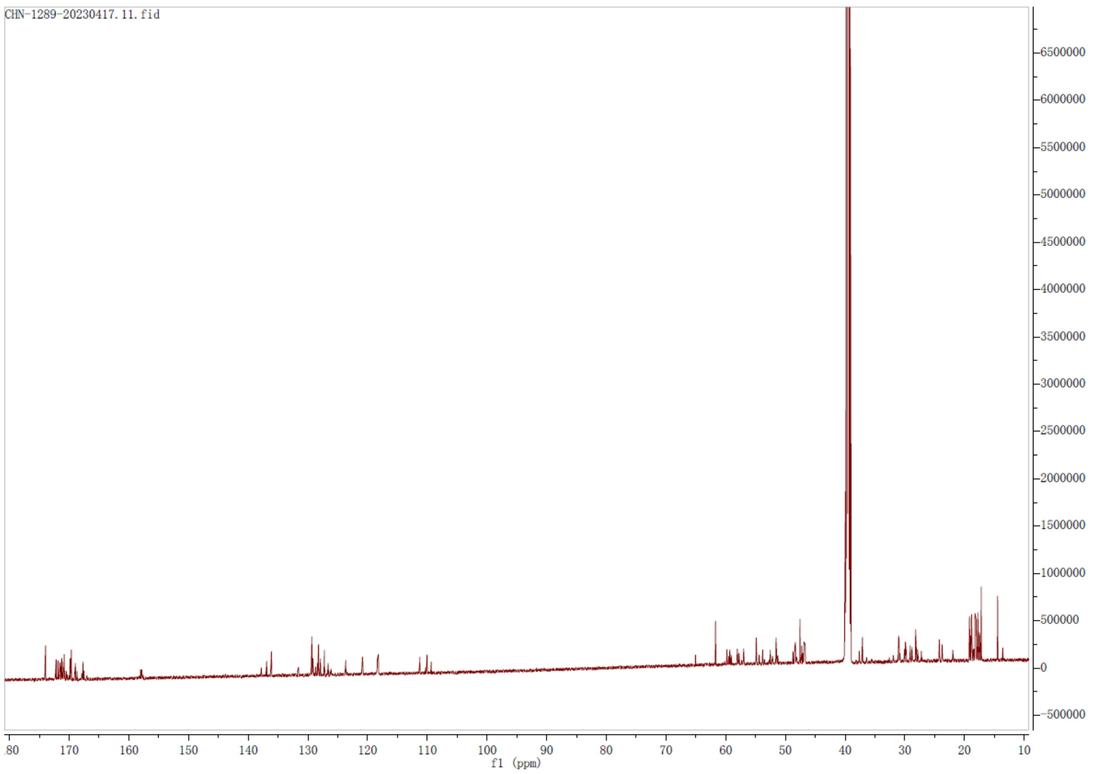

Supplementary Fig. 98. ^{13}C NMR spectrum of **1a** in DMSO-d_6 (150 MHz)

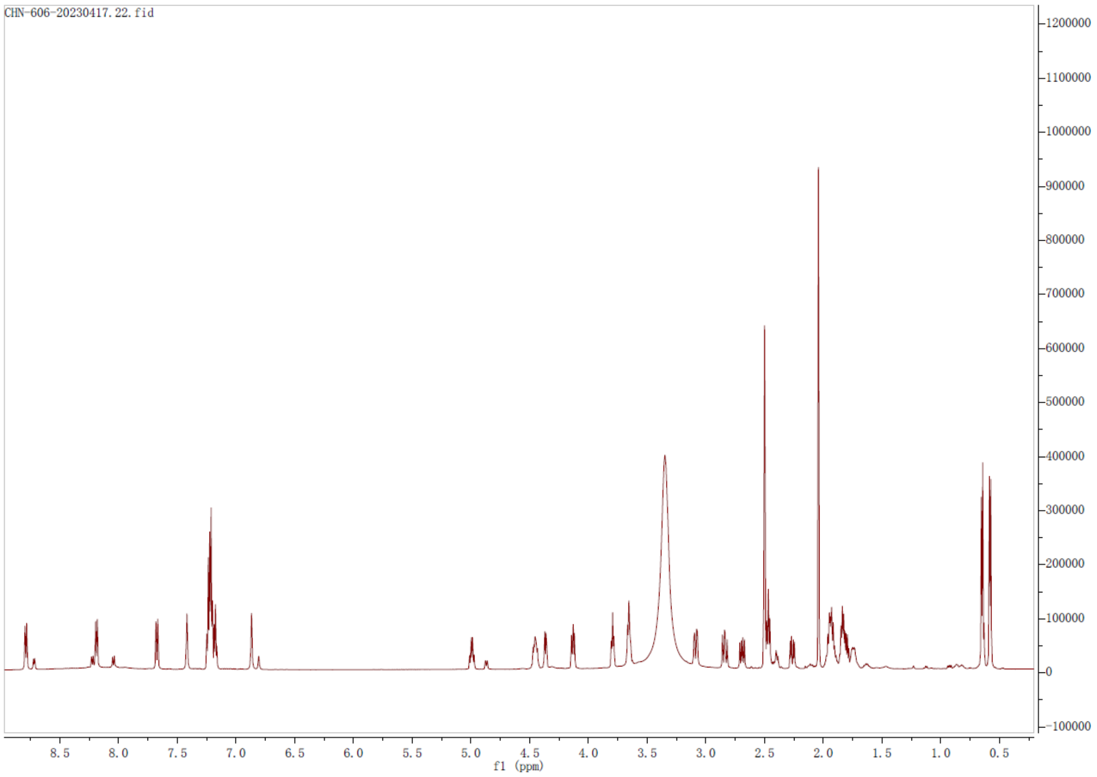

Supplementary Fig. 99. ^{1}H NMR spectrum of **13a** in DMSO-d_6 (600 MHz)

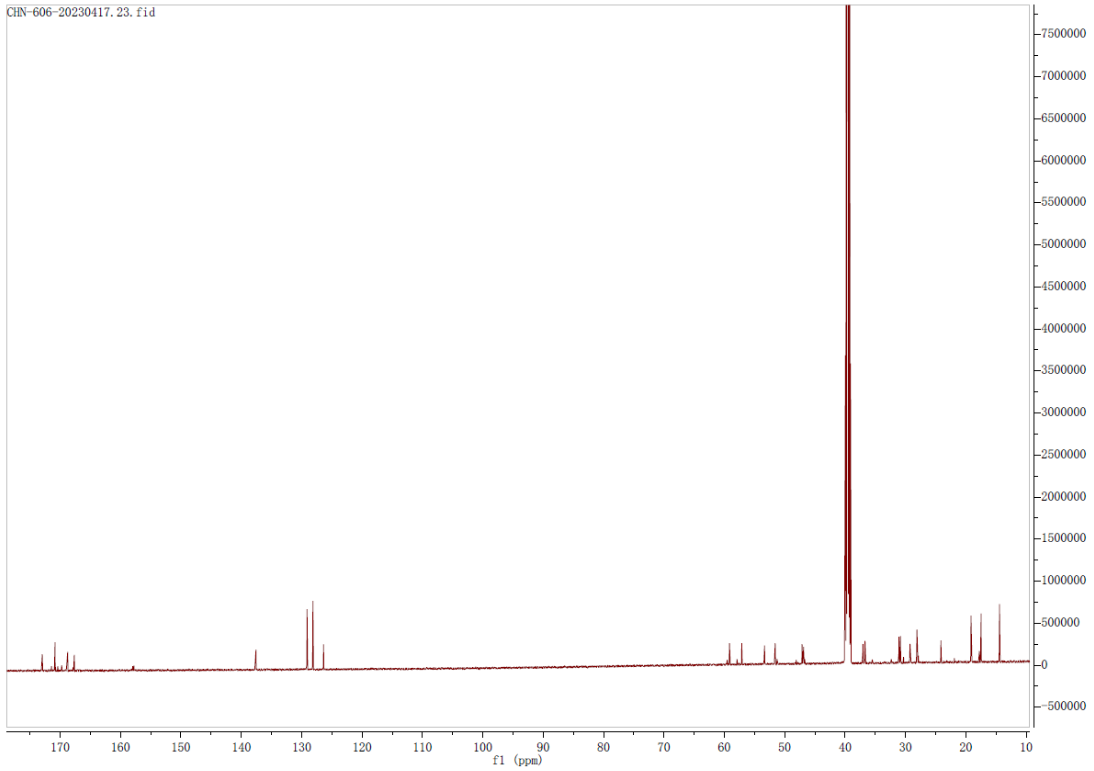

Supplementary Fig. 100. ^{13}C NMR spectrum of **13a** in DMSO-d_6 (150 MHz)

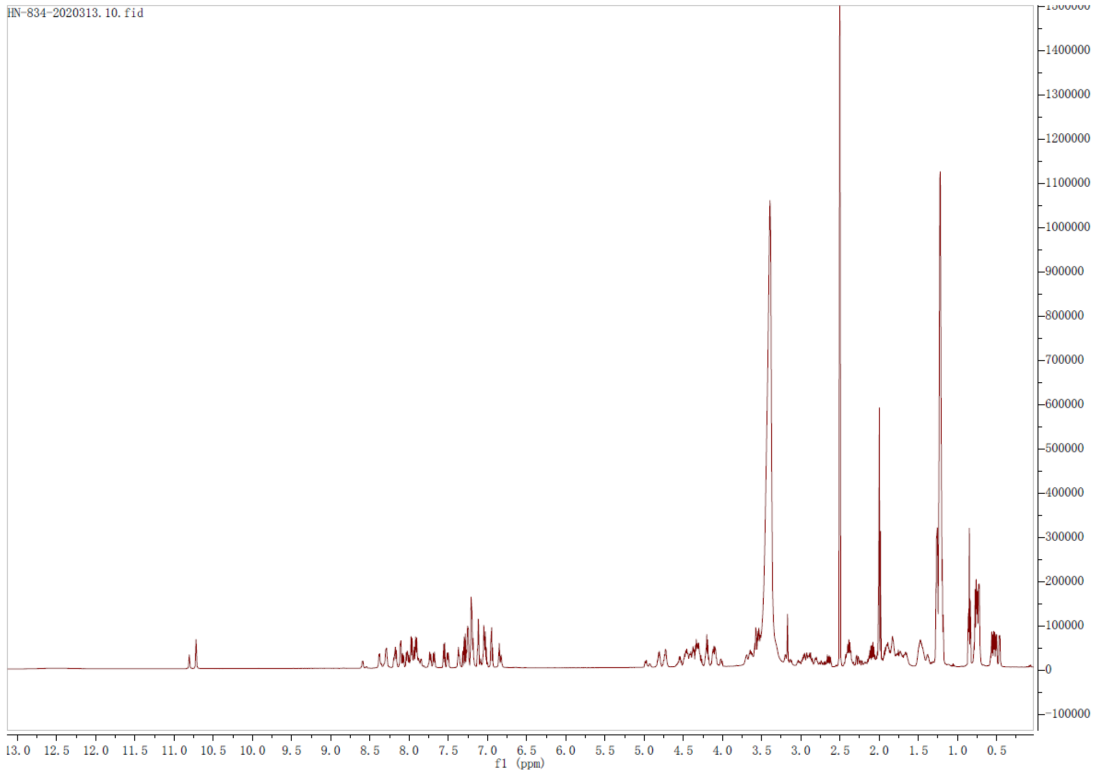

Supplementary Fig. 101. ^{1}H NMR spectrum of **10a** in DMSO-d_6 (600 MHz)

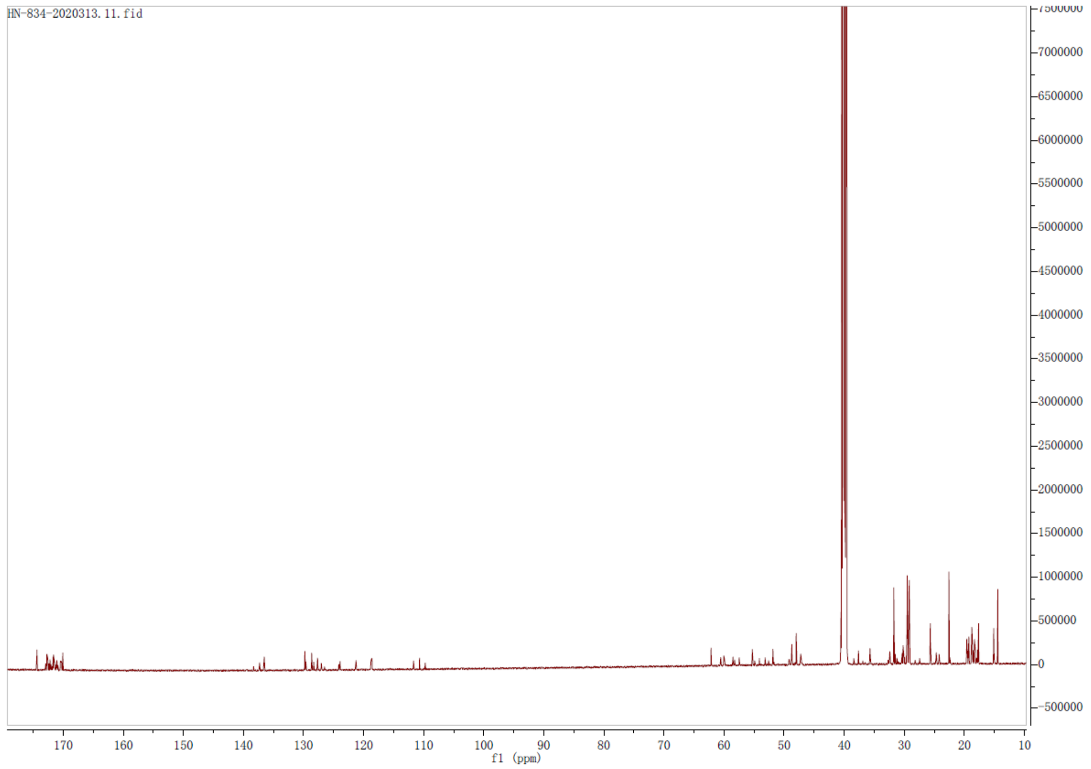

Supplementary Fig. 102. ^{13}C NMR spectrum of **10a** in DMSO-d_6 (150 MHz)

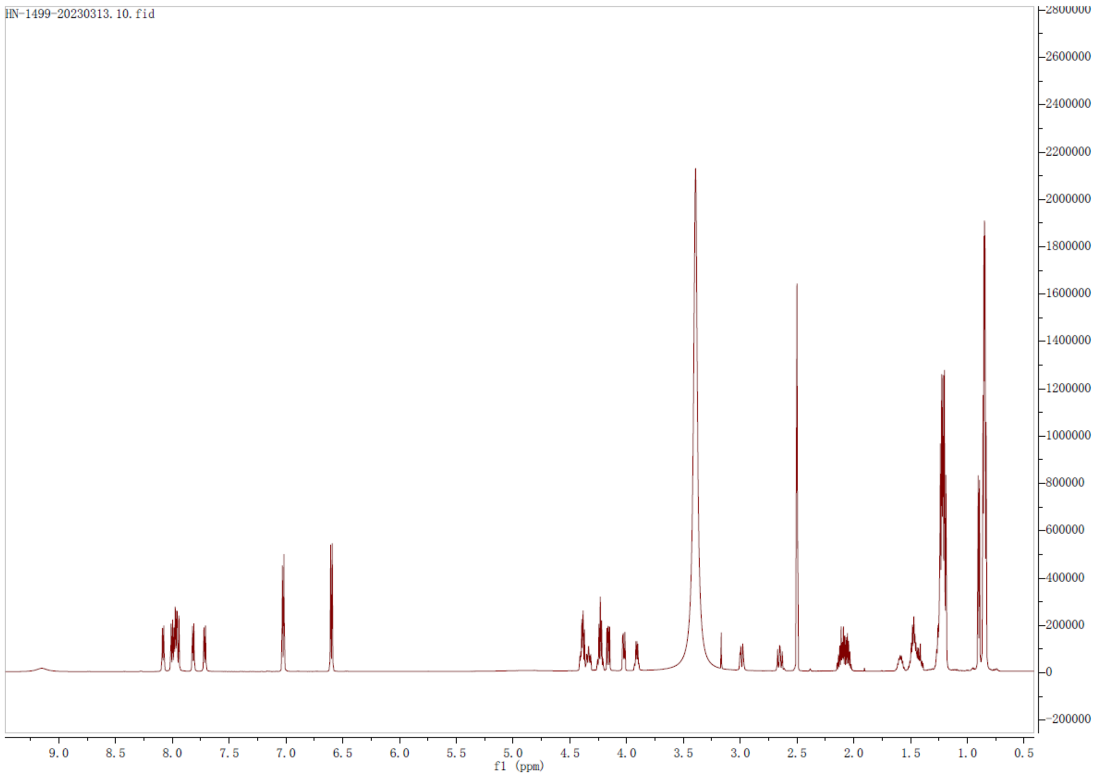

Supplementary Fig. 103. ^{1}H NMR spectrum of **29a** in DMSO-d_6 (600 MHz)

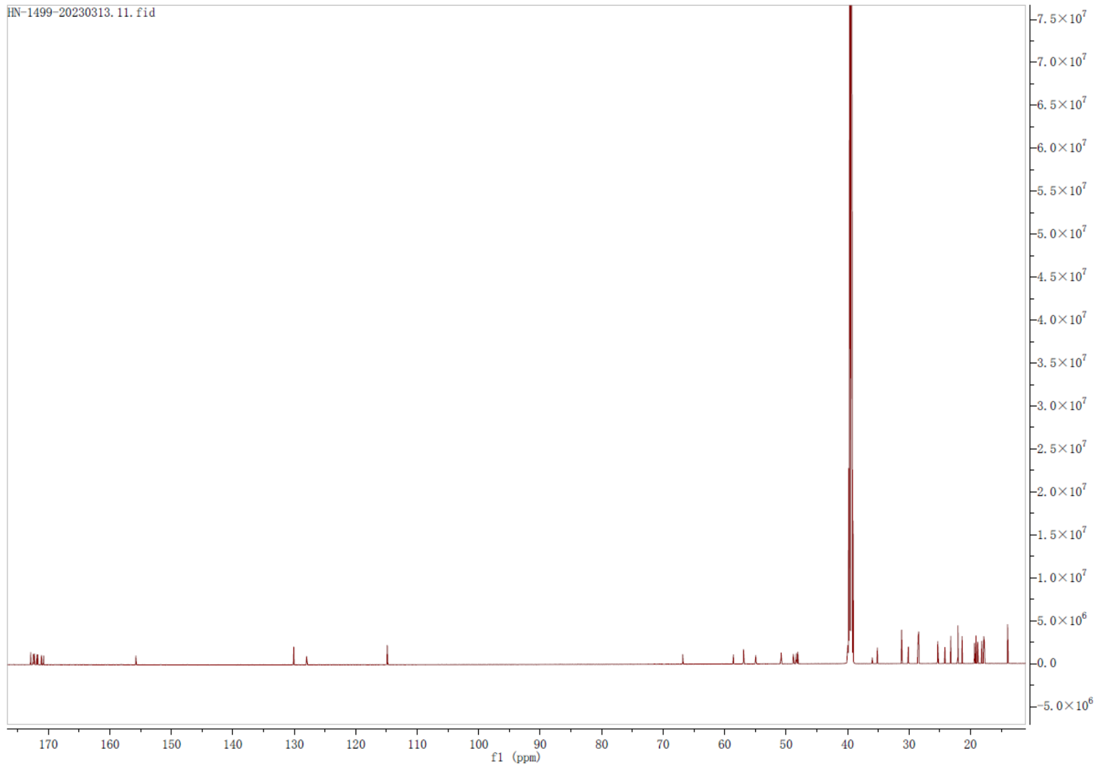

Supplementary Fig. 104. ^{13}C NMR spectrum of **29a** in DMSO-d_6 (150 MHz)

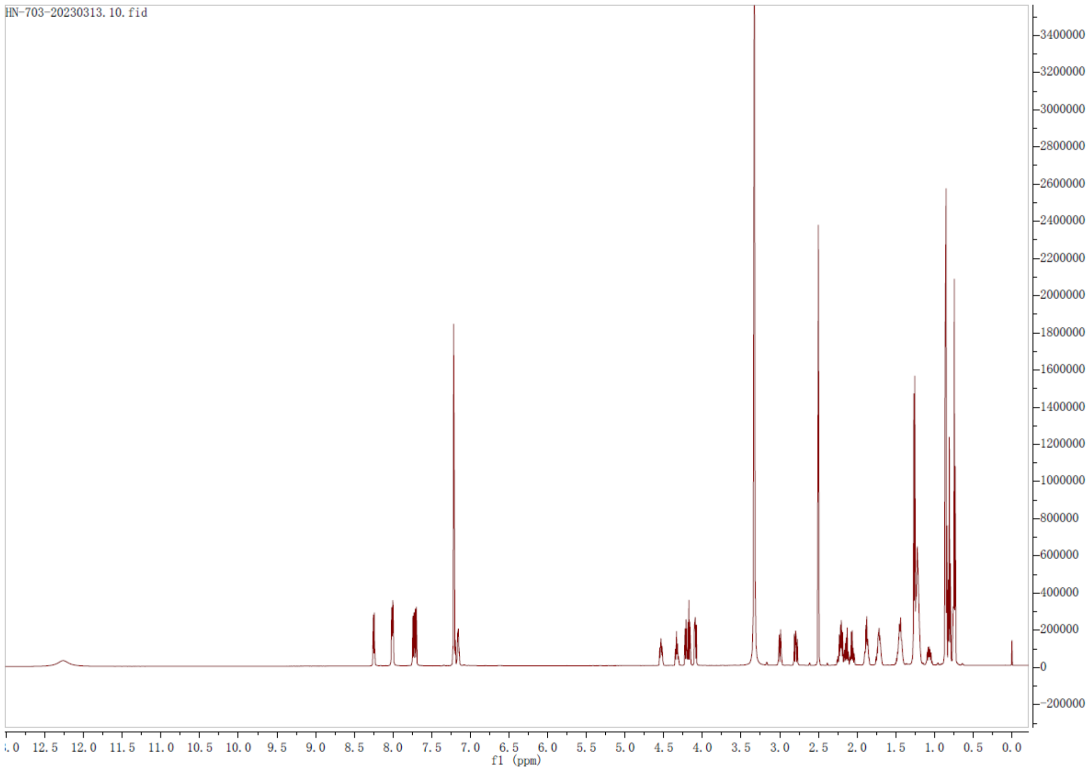

Supplementary Fig. 105. ^{1}H NMR spectrum of **31a** in DMSO-d_6 (600 MHz)

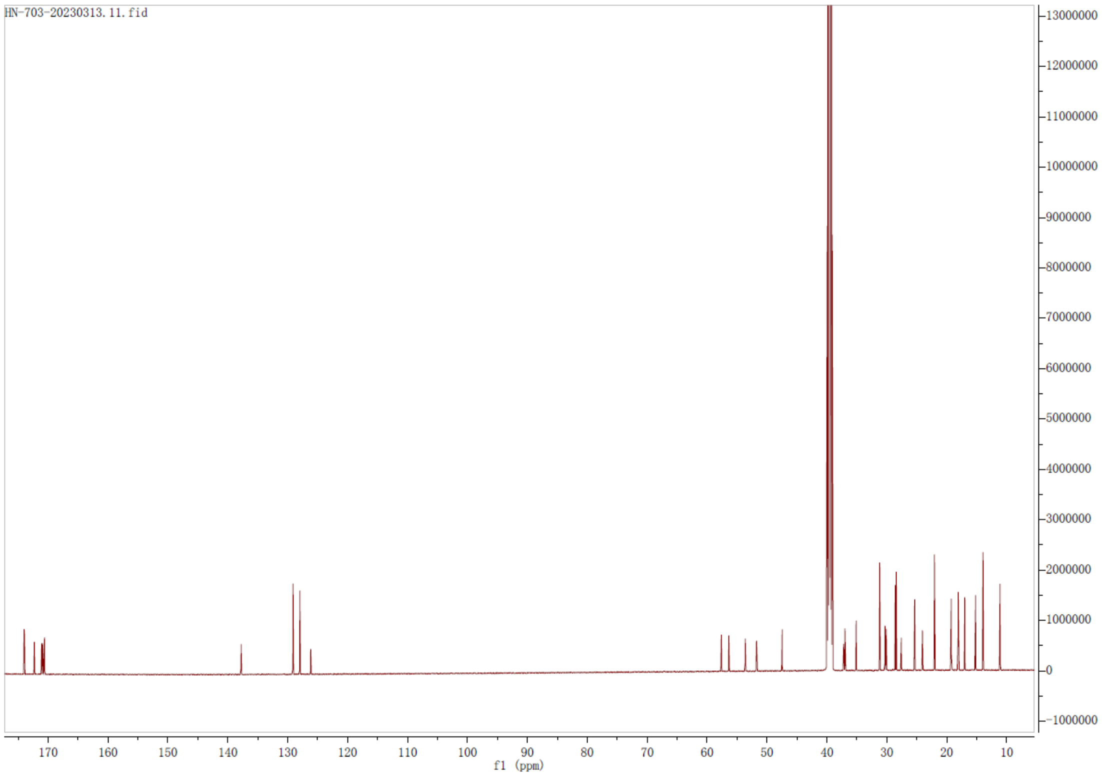

Supplementary Fig. 106. ^{13}C NMR spectrum of **31a** in DMSO-d_6 (150 MHz)

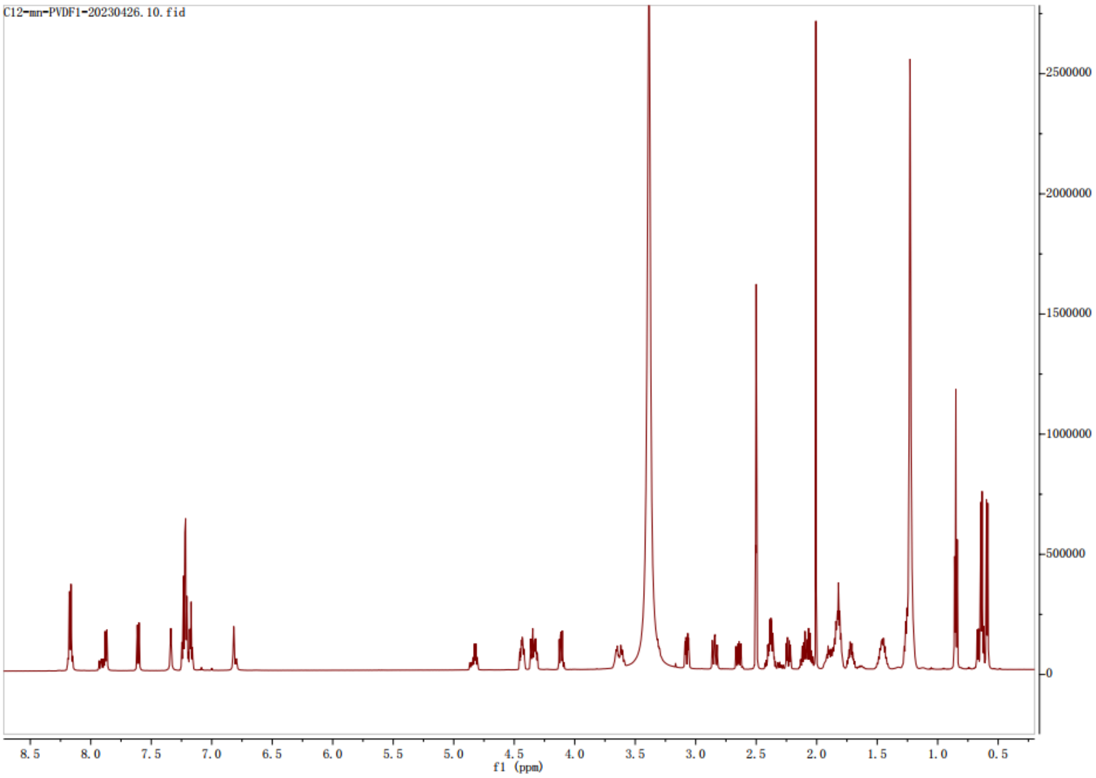

Supplementary Fig. 107. ^{1}H NMR spectrum of **13** in DMSO-d_6 (600 MHz)

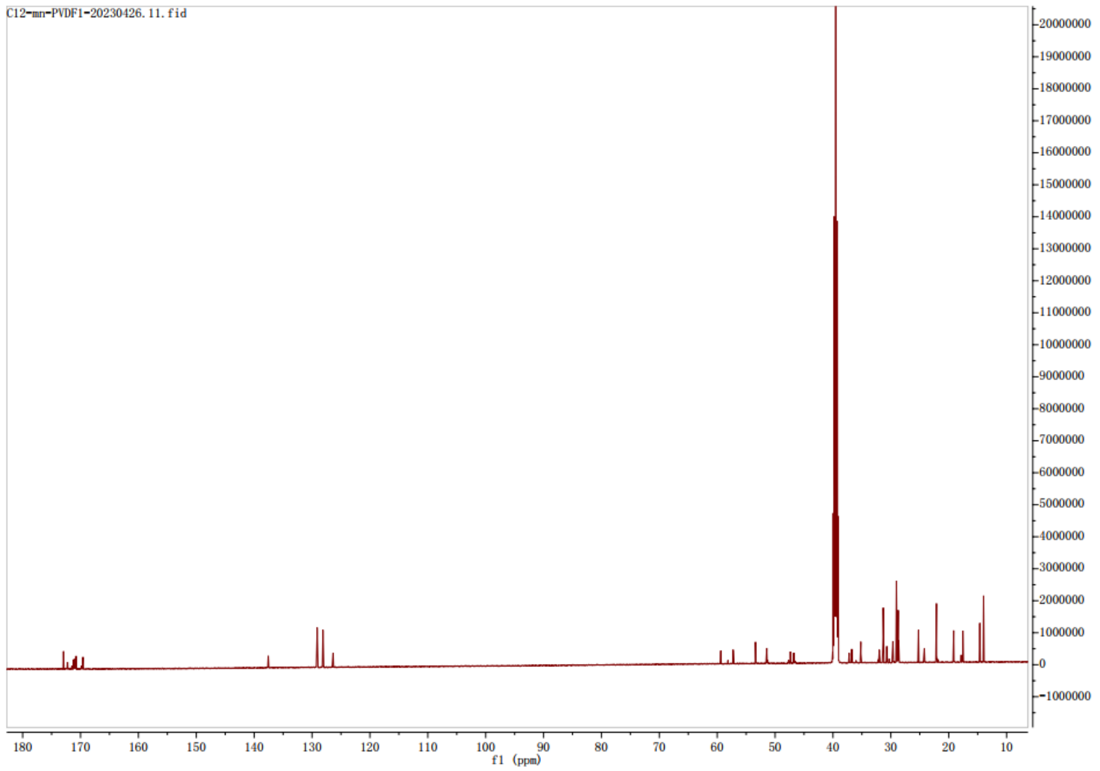

Supplementary Fig. 108. ^{13}C NMR spectrum of **13** in DMSO-d_6 (150 MHz)

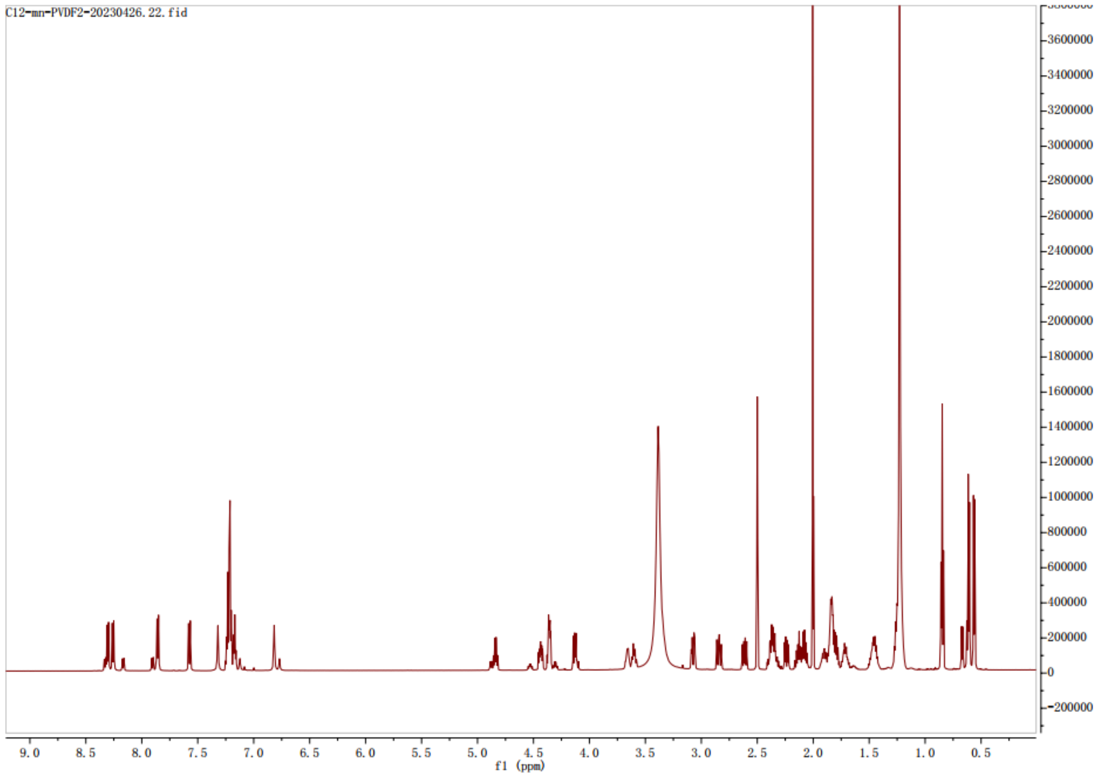

Supplementary Fig. 109. ^{1}H NMR spectrum of **14** in DMSO-d_6 (600 MHz)

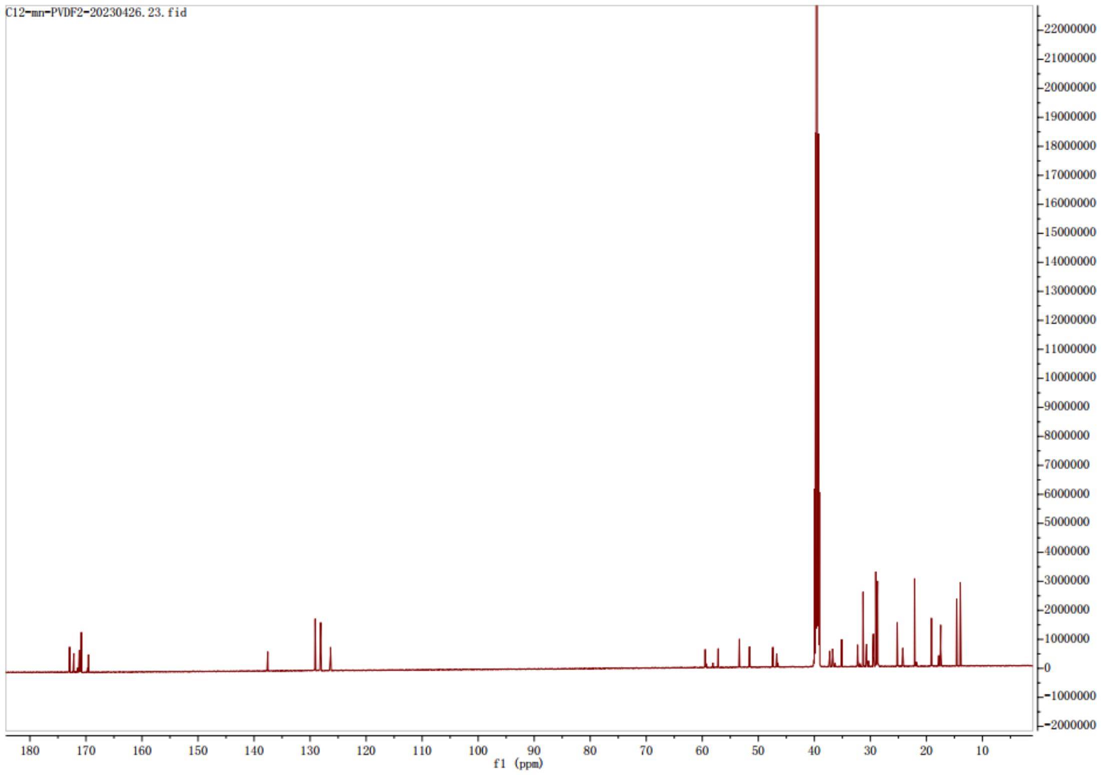

Supplementary Fig. 110. ^{13}C NMR spectrum of **14** in DMSO-d_6 (150 MHz)

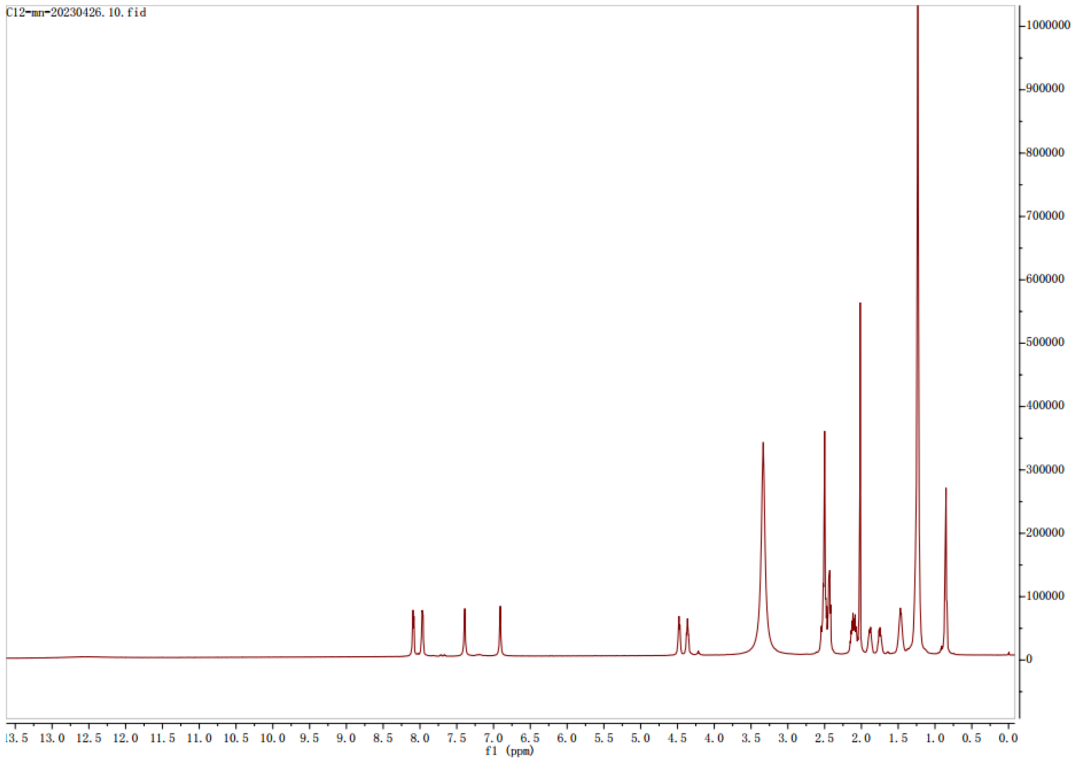

Supplementary Fig. 111. ^{1}H NMR spectrum of **15** in DMSO-d_6 (600 MHz)

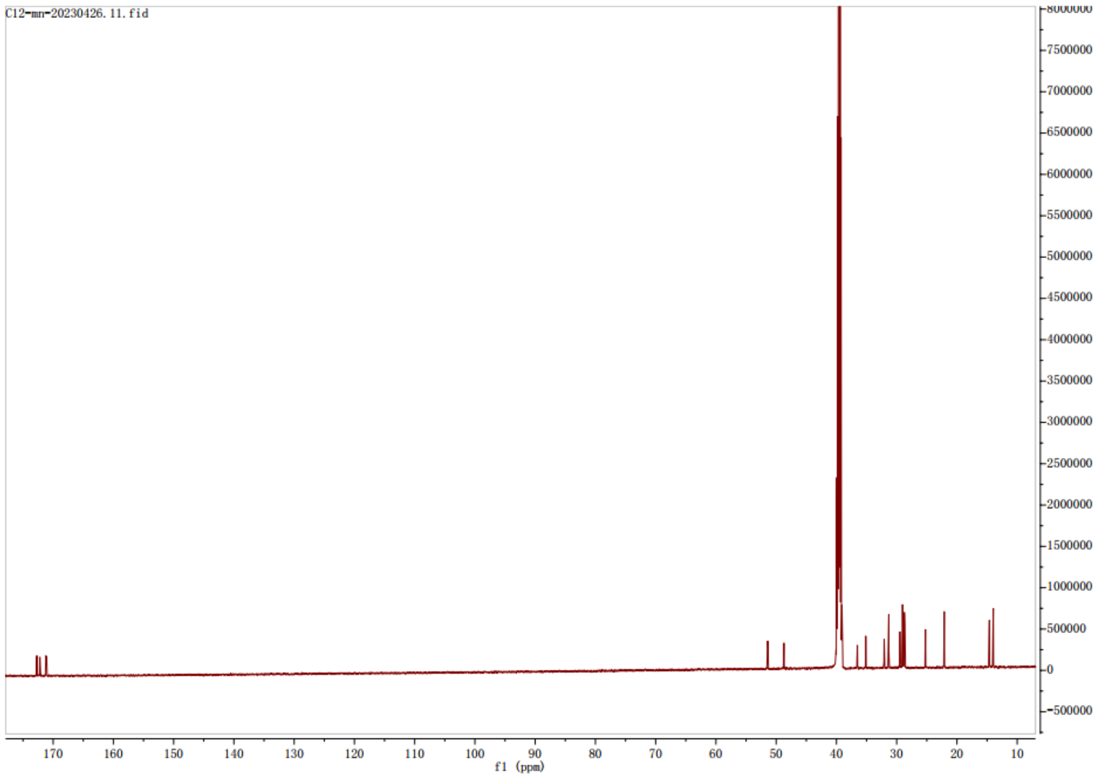

Supplementary Fig. 112. ^{13}C NMR spectrum of **15** in DMSO-d_6 (150 MHz)

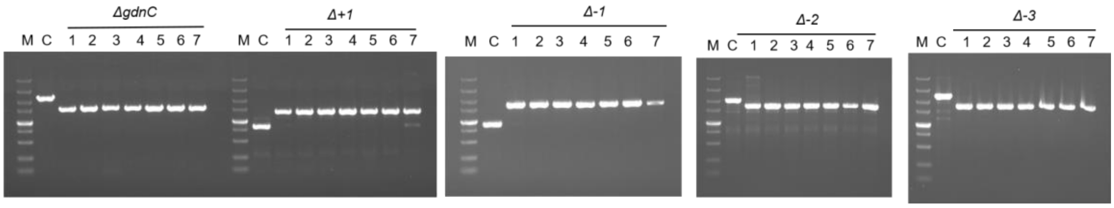

The uncropped scans of gels in Supplementary Figure 2b

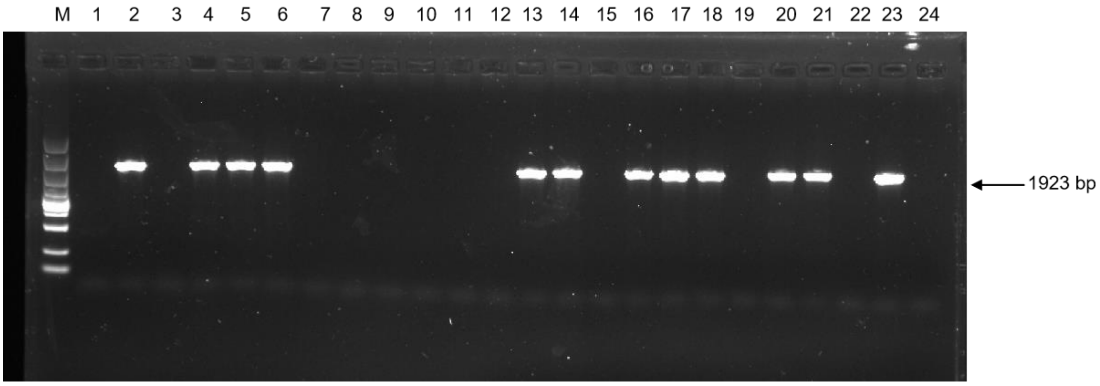

The uncropped scan of gels in Supplementary Figure 4d

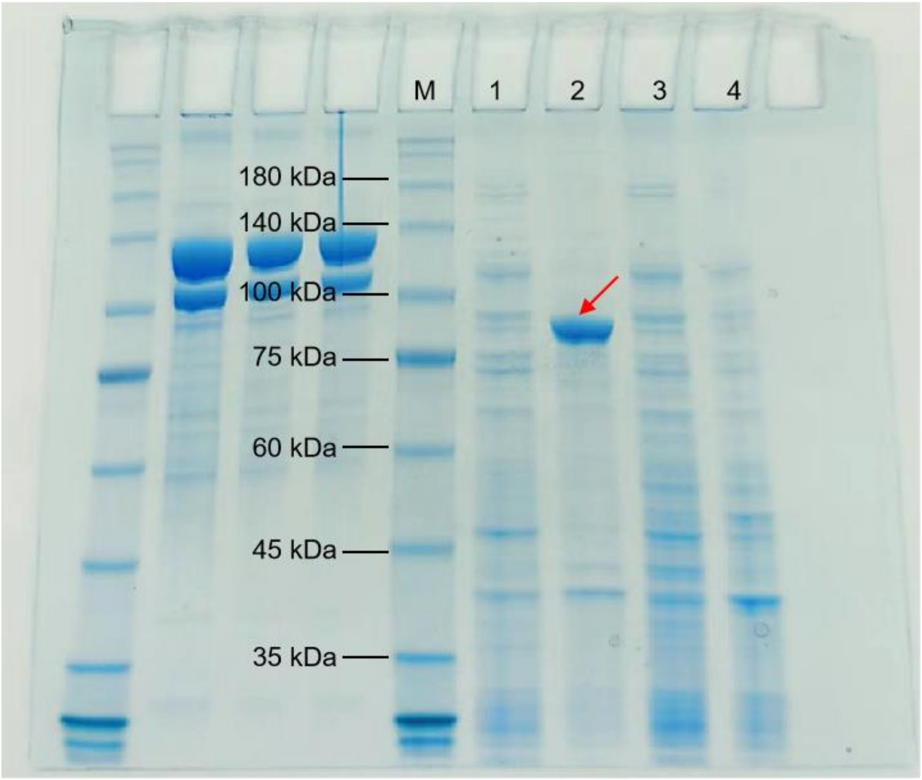

The uncropped scan of gels in Supplementary Figure 5a

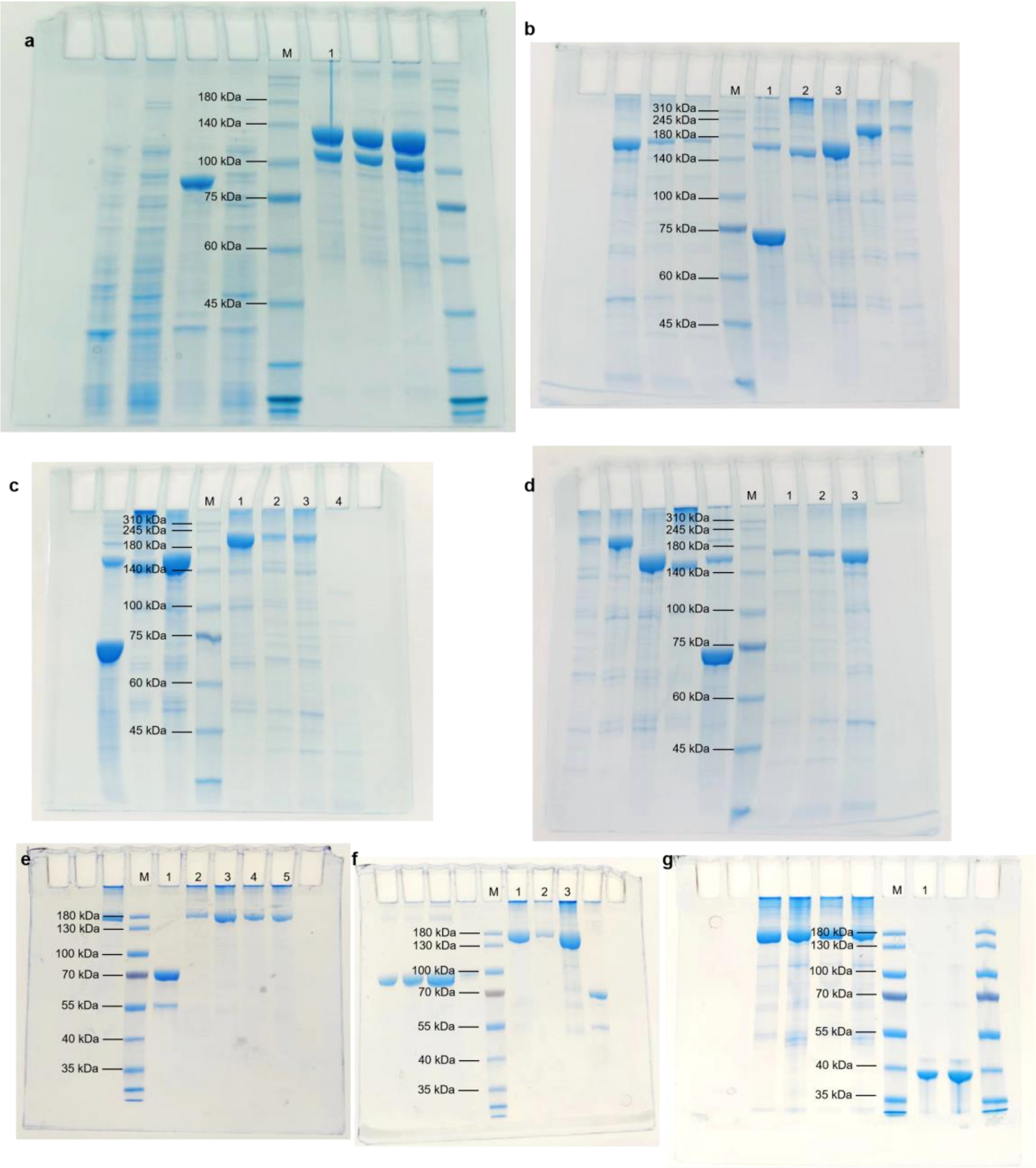

The uncropped scans of gels in Supplementary Figure 7

Supplementary References

(1) Jumper, J., Evans, R., Pritzel, A., Green, T., Figurnov, M., Ronneberger, O., Tunyasuvunakool, K., Bates, R., Žídek, A., Potapenko, A., Bridgland, A., Meyer, C., Kohl, S. A. A., Ballard, A. J., Cowie, A., Romera-Paredes, B., Nikolov, S., Jain, R., Adler, J., Back, T., Petersen, S., Reiman, D., Clancy, E., Zielinski, M., Steinegger, M., Pacholska, M., Berghammer, T., Bodenstein, S., Silver, D., Vinyals, O., Senior, A. W., Kavukcuoglu, K., Kohli, P. & Hassabis, D. Highly accurate protein structure prediction with AlphaFold. *Nature*, **596**, 583-589 (2021).

(2) Drake, E. J. & Gulick, A. M. Structural characterization and high-throughput screening of inhibitors of PvdQ, an NTN hydrolase involved in pyoverdine synthesis. *ACS Chem. Biol.*, **6**, 1277-1286 (2011).

(3) Bokhove, M., Jimenez, P. N., Quax, W. J. & Dijkstra, B. W. The quorum-quenching N-acyl homoserine lactone acylase PvdQ is an Ntn-hydrolase with an unusual substrate-binding pocket. *Proc. Natl. Acad. Sci. U S A.*, **107**, 686-691 (2010).

(4) Clevenger, K. D., Mascarenhas, R., Catlin, D., Wu, R., Kelleher, N. L., Drake, E. J., Gulick, A. M., Liu, D. L. & Fast, W. Substrate trapping in the siderophore tailoring enzyme PvdQ. *ACS Chem. Biol.*, **12**, 643-647 (2017).

(5) Clevenger, K. D., Wu, R., Er, J. A. V., Liu, D. L. & Fast, W. Rational design of a transition state analogue with picomolar affinity for *Pseudomonas aeruginosa* PvdQ, a siderophore biosynthetic enzyme. *ACS Chem. Biol.*, **8**, 2192-2200 (2013).

(6) Fu, C. Z., Keller, L., Bauer A., Brönstrup, M., Froidbise, A., Hammann, P., Herrmann, J., Mondesert, G., Kurz, M., Schiell, M., Schummer, D., Toti, L., Wink, J. & Müller, R. Biosynthetic studies of telomycin reveal new lipopeptides with enhanced activity. *J. Am. Chem. Soc.*, **137**, 7692-7705 (2015).

(7) Baars, O., Zhang, X. N., Gibson, M. I., Stone, A. T., Morel, F. M. M. & Seyedsayamdost, M. R. Crochelins: siderophores with an unprecedented iron-chelating moiety from the nitrogen-fixing bacterium *Azotobacter chroococcum*. *Angew. Chem. Int. Ed. Engl.*, **57**, 536-541 (2018).

(8) Gauglitz, J. M., Iinishi, A., Ito, Y. & Butler, A. Microbial tailoring of acyl peptidic siderophores. *Biochemistry*, **53**, 2624-2631 (2014).

(9) Laursen, B. S., Sørensen, H. P., Mortensen, K. K. & Sperling-Petersen, H. U. Initiation of protein synthesis in bacteria. *Microb. Mol. Biol. Rev.*, **69**, 101-123 (2005).

(10) Schoenafinger, G., Schracke, N., Linne, U. & Marahiel, M. A. Formylation domain: an essential modifying enzyme for the nonribosomal biosynthesis of linear gramicidin. *J. Am. Chem. Soc*., **128**, 7406-7407 (2006).

(11) Rouhiainen, L., Paulin, L., Suomalainen, S., Hyytiäinen, H., Buikema, W., Haselkorn, R. & Sivonen, K. Genes encoding synthetases of cyclic depsipeptides, anabaenopeptilides, in *Anabaena* strain 90. *Mol. Microbiol.*, **37**, 156-167 (2000).

(12) Ohlendorf, B., Simon, S., Wiese, J. & Imhoff. J. F. Szentiamide, an N-formylated cyclic depsipeptide from *Xenorhabdus szentirmaii* DSM 16338T. *Nat. Prod. Commun.*, **6**, 1247-1250 (2011)

(13) Bode, E., Heinrich, A. K., Hirschmann, M., Abebew, D., Shi, Y. N., Vo, T. D., Wesche, F., Shi, Y. M., Grün, P., Simonyi, S., Keller, N., Engel, Y., Wenski, S., Bennet, R., Beyer, S., Bischoff, I., Buaya, A., Brandt, S., Cakmak, I., Çimen, H., Eckstein, S., Frank, D., Fürst, R., Gand, M., Geisslinger, G., Hazir, S., Henke, M., Heermann, R., Lecaudey, V., Schäfer, W., Schiffmann, S., Schüffler, A., Schwenk, R., Skaljac, M., Thines, E., Thines, M., Ulshöfer, T., Vilcinskas, A., Wichelhaus, T. A. & Bode, H. B. Promoter activation in Δ*hfq* mutants as an efficient tool for specialized metabolite production enabling direct bioactivity testing. *Angew. Chem. Int. Ed. Engl.*, **58**, 18957-18963 (2019).
