# Supplementary material for: Biosynthesis and engineering of the nonribosomal peptides with a C-terminal putrescine
Source: Nat Commun. 2023 Oct 19;14:6619. doi: 10.1038/s41467-023-42387-z (PMC10587159; doi:10.1038/s41467-023-42387-z)
Supplement: Supplementary file 3 — Reporting Summary [file 41467_2023_42387_MOESM3_ESM.pdf]

nature portfolio

Corresponding author(s): Xiaoying Bian

Last updated by author(s): Sep 18, 2023

Reporting Summary

Nature Portfolio wishes to improve the reproducibility of the work that we publish. This form provides structure for consistency and transparency in reporting. For further information on Nature Portfolio policies, see our Editorial Policies and the Editorial Policy Checklist.

Statistics

For all statistical analyses, confirm that the following items are present in the figure legend, table legend, main text, or Methods section.

n/a	Confirmed	
☐	☒	The exact sample size (*n*) for each experimental group/condition, given as a discrete number and unit of measurement
☐	☒	A statement on whether measurements were taken from distinct samples or whether the same sample was measured repeatedly
☐	☒	The statistical test(s) used AND whether they are one- or two-sided<br>*Only common tests should be described solely by name; describe more complex techniques in the Methods section.*
☒	☐	A description of all covariates tested
☒	☐	A description of any assumptions or corrections, such as tests of normality and adjustment for multiple comparisons
☐	☒	A full description of the statistical parameters including central tendency (e.g. means) or other basic estimates (e.g. regression coefficient) AND variation (e.g. standard deviation) or associated estimates of uncertainty (e.g. confidence intervals)
☐	☒	For null hypothesis testing, the test statistic (e.g. *F*, *t*, *r*) with confidence intervals, effect sizes, degrees of freedom and *P* value noted<br>*Give P values as exact values whenever suitable.*
☒	☐	For Bayesian analysis, information on the choice of priors and Markov chain Monte Carlo settings
☒	☐	For hierarchical and complex designs, identification of the appropriate level for tests and full reporting of outcomes
☒	☐	Estimates of effect sizes (e.g. Cohen's *d*, Pearson's *r*), indicating how they were calculated

Our web collection on statistics for biologists contains articles on many of the points above.

Software and code

Policy information about availability of computer code

Data collection	UPLC/MS or UPLC/HRMS data were collected from Ultimate 3000 UPLC (Thermo Fisher) connected to Bruker amaZon SL ESI - Ion Trap Mass Spectrometer or Bruker Impact HD Mass Spectrometer; NMR data were collected from Bruker Avance III-600/500 spectrometer; Ligands were docking with protein by Autodock; The data of anti-tumor and anti-inflammatory activity tests in vitro were collected by microplate reader (Infinite M200, Tecan).
Data analysis	DataAnalysis (v4.2); Microsoft Word & Excel & PowerPoint (2019); Alphafold2; PyMOL (v2.6); AutoDock (v4.2.6)

For manuscripts utilizing custom algorithms or software that are central to the research but not yet described in published literature, software must be made available to editors and reviewers. We strongly encourage code deposition in a community repository (e.g. GitHub). See the Nature Portfolio guidelines for submitting code & software for further information.

Data

Policy information about availability of data

All manuscripts must include a data availability statement. This statement should provide the following information, where applicable:

- Accession codes, unique identifiers, or web links for publicly available datasets
- A description of any restrictions on data availability
- For clinical datasets or third party data, please ensure that the statement adheres to our policy

Data supporting the findings of this work are available within the paper and its Supplementary Information files. A reporting summary for this Article is available as a

Supplementary Information file. The following public databases were utilized for the analysis of the protein structures: 5UBK [https://doi.org/10.2210/pdb5UBK/pdb], 7C1S [https://doi.org/10.2210/pdb7C1S/pdb], 4ZXI [https://doi.org/10.2210/pdb4ZXI/pdb], 3L91 [https://doi.org/10.2210/pdb3L91/pdb], 1JMK [https://doi.org/10.2210/pdb1JMK/pdb]. Source data are provided with this paper.

Human research participants

Policy information about studies involving human research participants and Sex and Gender in Research.

Reporting on sex and gender	n/a
Population characteristics	n/a
Recruitment	n/a
Ethics oversight	n/a

Note that full information on the approval of the study protocol must also be provided in the manuscript.

Field-specific reporting

Please select the one below that is the best fit for your research. If you are not sure, read the appropriate sections before making your selection.

☒ Life sciences ☐ Behavioural & social sciences ☐ Ecological, evolutionary & environmental sciences

For a reference copy of the document with all sections, see nature.com/documents/nr-reporting-summary-flat.pdf

Life sciences study design

All studies must disclose on these points even when the disclosure is negative.

Sample size	Sample size was always base on experimental feasibility and sample availability. Sample size was determined as generation of triple independent samples for comparisons between groups that is sufficient to perform statistical tests.
Data exclusions	No data were excluded.
Replication	The experimental findings in all figures were reproduced successfully. The quantifications of compounds in vivo or in vitro experiments were performed independently and in triplicates or quantifications stated in figure legends.
Randomization	Randomization was not necessary, all subsequent tests of quantification were performed in triplicates/quadruplicate.
Blinding	Not applicable, data were automatic readouts of the instruments in this work and no animal or clinical experiments were involved.

Reporting for specific materials, systems and methods

We require information from authors about some types of materials, experimental systems and methods used in many studies. Here, indicate whether each material, system or method listed is relevant to your study. If you are not sure if a list item applies to your research, read the appropriate section before selecting a response.

Materials & experimental systems

n/a	Involved in the study	
☒	☐	Antibodies
☐	☒	Eukaryotic cell lines
☒	☐	Palaeontology and archaeology
☒	☐	Animals and other organisms
☒	☐	Clinical data
☒	☐	Dual use research of concern

Methods

n/a	Involved in the study	
☒	☐	ChIP-seq
☒	☐	Flow cytometry
☒	☐	MRI-based neuroimaging

Eukaryotic cell lines

Policy information about cell lines and Sex and Gender in Research

Cell line source(s)	Human acute lymphoblastic leukemia cells Kasumi and K562 used in cytotoxicity assay was obtained from ATCC. Human

Cell line source(s)	breast cancer cells MDA-MB-231 and MCF7, human lung carcinoma cell line A549, human hepatoma cell HepG-2, human gastric cancer cell SGC-7901, human colon cancer cell HCT-116, human ovarian cancer cell line SKOV3, and human normal lung epithelial cells line 2B used in cytotoxicity assay were obtained from Beyotime Biotechnology. Macrophage RAW246.7 used in anti-inflammatory assay was obtained from Shanghai Institutes for Biological Science.
Authentication	The ATCC and Beyotime Biotechnology used STR genotyping for cell line authentication including Shanghai Institutes for Biological Science. Additional authentication in the laboratory was performed regularly based on morphology and gene/protein expression.
Mycoplasma contamination	Cell lines were periodically tested for mycoplasma contamination and confirmed negative.
Commonly misidentified lines (See ICLAC register)	No commonly misidentified cell lines were used.
