# Supplementary material for: Biosynthesis and engineering of the nonribosomal peptides with a C-terminal putrescine
Source: Nat Commun. 2023 Oct 19;14:6619. doi: 10.1038/s41467-023-42387-z (PMC10587159; doi:10.1038/s41467-023-42387-z)
Supplement: Supplementary file 4 — Description of Additional Supplementary Files [file 41467_2023_42387_MOESM4_ESM.pdf]

Description of Additional Supplementary Files

Source Data

Description: The bioactivity of A_1 domain to various amino acids, and the function study of C-terminal NRPS protein through comparing the yields of target compounds *in vivo* and *in vitro*. The growth characteristics of DSM 7029 and its mutants, as well as dealt with different compounds.

Supplementary Data 1

Description: Bacteria strains, plasmids and primers used in this study.
